# Supplementary material for: The current burden of Japanese encephalitis and the estimated impacts of vaccination: Combining estimates of the spatial distribution and transmission intensity of a zoonotic pathogen
Source: PLoS Negl Trop Dis. 2021 Oct 13;15(10):e0009385. doi: 10.1371/journal.pntd.0009385 (PMC8544850; doi:10.1371/journal.pntd.0009385)
Supplement: S1 Table — (PDF) [file pntd.0009385.s001.pdf]

Table S1. Summary information and results for studies of age-specific Japanese Encephalitis (JE) incidence used to estimate the annual force of infection (FOI). Results are the median posterior FOI estimates with 95% credible interval. Vaccination coverage was assumed to be 0 for studies without a vaccination data source.

Country	Study period	FOI (95% CrI)	Incidence data	Vaccination data
Bangladesh	2007-2008	0.148 (0.087 - 0.195)	[23]	
Cambodia	2006-2008	0.067 (0.026 - 0.107)	[28]	
Cambodia	2010-2013	0.096 (0.064 - 0.129)	[6]	
China	2004-2014	0.083 (0.041 - 0.128)	[12]	2000-2014: [30]
China	2000-2005	0.193 (0.130 - 0.244)	[29]	2000-2005: [30]
India	2008-2012	0.164 (0.044 - 0.325)	[26]	2006-2012: [30]
India	2000-2002	0.056 (0.047 - 0.066)	[24]	
India	2011-2013	0.162 (0.038 - 0.279)	[8]	2006-2013: [30]
Indonesia	2001-2003	0.197 (0.116 - 0.284)	[10]	
Indonesia	2005-2006	0.366 (0.289 - 0.457)	[20]	
Japan	1982-2004	0.006 (0.000 - 0.016)	[1]	1976-2004: [25]
Laos	2001-2008	0.040 (0.021 - 0.063)	[16]	
Malaysia	2006-2013	0.050 (0.027 - 0.071)	[17]	2001-2013: [30]
Malaysia	1997-2001	0.289 (0.229 - 0.372)	[32]	
Nepal	2004-2006	0.059 (0.055 - 0.063)	[31]	
Nepal	1996-1997	0.071 (0.068 - 0.074)	[9]	
Nepal	2006	0.084 (0.055 - 0.118)	[22]	
Nepal	2007	0.161 (0.128 - 0.200)	[2]	
Nepal	2004-2006	0.125 (0.120 - 0.131)	[21]	
Philippines	2011-2014	0.178 (0.152 - 0.206)	[13]	
Philippines	2002-2004	0.044 (0.027 - 0.064)	[18]	
Philippines	1984	0.200 (0.162 - 0.245)	[14]	
South Korea	2001-2014	0.006 (0.000 - 0.031)	[11]	1963-1999: [15], [27]<br>2000-2005: [25]<br>2006-2014: [30]
Sri Lanka	2006-2010	0.020 (0.004 - 0.042)	[5]	1988-2008: [25]<br>2008-2010: Gavi
Taiwan	2000-2014	0.007 (0.000 - 0.033)	[3]	1969-2014: [7], [15]
Thailand	2003-2005	0.045 (0.013 - 0.081)	[19]	1990-2005: [19], [25]
Vietnam	1998-2007	0.105 (0.046 - 0.258)	[33]	1997-2005: [4]<br>2006-2007: [30]

References

1. Arai, S., Matsunaga, Y., Takasaki, T., Tanaka-Taya, K., Taniguchi, K., Okabe, N., Kurane, I., Japan, V.P.D.S.P. of, 2008. Japanese encephalitis: surveillance and elimination effort in Japan from 1982 to 2004. Jpn J Infect Dis 61, 333–338.
2. Bhattachan, A., Amatya, S., Sedai, T.R., Upreti, S.R., Partridge, J., 2009. Japanese Encephalitis in Hill and Mountain Districts, Nepal. Emerg Infect Dis 15, 1691–1692. https://doi.org/10.3201/eid1510.081641
3. Chang, Y.-K., Chang, H.-L., Wu, H.-S., Chen, K.-T., 2017. Epidemiological Features of Japanese Encephalitis in Taiwan from 2000 to 2014. The American Journal of Tropical Medicine and Hygiene 96, 382–388. https://doi.org/10.4269/ajtmh.16-0330
4. Choisy, M., 2017. Vaccine coverages from the WHO EPI in Vietnam [WWW Document]. URL https://github.com/choisy/epiVN (accessed 12.7.20).
5. Epidemiology unit MoH, S.L., 2012. Japanese Encephalitis: a manual for Medical Officers of Health. Ministry of Health.
6. Horwood, P.F., Duong, V., Laurent, D., Mey, C., Sothy, H., Santy, K., Richner, B., Heng, S., Hem, S., Cheval, J., Gorman, C., Dussart, P., de Jong, M.D., Kerleguer, A., Guillard, B., Murgue, B., Lecuit, M., de Lamballerie, X., Farrar, J.J., Tarantola, A., Eloit, M., Buchy, P., 2017. Aetiology of acute meningoencephalitis in Cambodian children, 2010–2013. Emerging Microbes & Infections 6, e35. https://doi.org/10.1038/emi.2017.15
7. Hsu, L.-C., Chen, Y.-J., Hsu, F.-K., Huang, J.-H., Chang, C.-M., Chou, P., Lin, I.-F., Chang, F.-Y., 2014. The Incidence of Japanese Encephalitis in Taiwan—A Population-Based Study. PLoS Negl Trop Dis 8. https://doi.org/10.1371/journal.pntd.0003030
8. Jain, P., Singh, A.K., Khan, D.N., Pandey, M., Kumar, R., Garg, R., Jain, A., 2016. Trend of Japanese encephalitis in Uttar Pradesh, India from 2011 to 2013. Epidemiology & Infection 144, 363–370. https://doi.org/10.1017/S0950268815000928
9. Joshi, D.D., 2007. Epidemiological Profile of Japanese Encephalities in Nepal 1996-1997. Journal of Institute of Medicine 22.
10. Kari, K., Liu, W., Gautama, K., Mammen, M.P., Clemens, J.D., Nisalak, A., Subrata, K., Kim, H.K., Xu, Z.-Y., 2006. A hospital-based surveillance for Japanese encephalitis in Bali, Indonesia. BMC Med 4, 8. https://doi.org/10.1186/1741-7015-4-8
11. Lee, E.J., Cha, G.-W., Ju, Y.R., Han, M.G., Lee, W.-J., Jeong, Y.E., 2016. Prevalence of Neutralizing Antibodies to Japanese Encephalitis Virus among High-Risk Age Groups in South Korea, 2010. PLOS ONE 11, e0147841. https://doi.org/10.1371/journal.pone.0147841
12. Li, X., Cui, S., Gao, X., Wang, H., Song, M., Li, M., Fu, S., Lv, Z., He, Y., Lei, W., Wang, B., Lu, X., Liang, G., 2016. The Spatio-temporal Distribution of Japanese Encephalitis Cases in Different Age Groups in Mainland China, 2004 – 2014. PLOS Neglected Tropical Diseases 10, e0004611. https://doi.org/10.1371/journal.pntd.0004611
13. Lopez, A.L., Aldaba, J.G., Jr, V.G.R., Iii, A.O.T., Sy, A.K., Espino, F.E., DeQuiroz-Castro, M., Jee, Y., Ducusin, M.J., Fox, K.K., 2015. Epidemiology of Japanese Encephalitis in the Philippines: A Systematic Review. PLOS Neglected Tropical Diseases 9, e0003630. https://doi.org/10.1371/journal.pntd.0003630
14. Luis, A.S., Hayes, C.G., Manaloto, C.R., n.d. The Neurologic Features of Japanese Encephalitis in the Philippines.
15. Monath, T.P., 2002. Japanese Encephalitis Vaccines: Current Vaccines and Future Prospects, in: Mackenzie, J.S., Barrett, A.D.T., Deubel, V. (Eds.), Japanese Encephalitis and West Nile Viruses, Current Topics in Microbiology and Immunology. Springer, Berlin, Heidelberg, pp. 105–138. https://doi.org/10.1007/978-3-642-59403-8_6
16. Moore, C.E., Blacksell, S.D., Taojaikong, T., Jarman, R.G., Gibbons, R.V., Lee, S.J., Chansamouth, V., Thongpaseuth, S., Mayxay, M., Newton, P.N., 2012. A prospective assessment of the accuracy of commercial IgM ELISAs in diagnosis of Japanese encephalitis virus infections in patients with

suspected central nervous system infections in Laos. Am. J. Trop. Med. Hyg. 87, 171–178. https://doi.org/10.4269/ajtmh.2012.11-0729
17. Mustapa, N.I., Saraswathy, T.S., Ruslan, N.A.M., Kassim, F.M., Saat, Z., 2016. Japanese Encephalitis in Malaysia: Review of laboratory data from 2006 to 2013. Southeast Asian Journal of Tropical Medicine and Public Health 47, 759–765.
18. Natividad, F.F., Daroy, M.L.G., Alonzo, M.T., Matias, R.R., Suarez, L. a. C., Inoue, S., 2006. Use of IgM-capture ELISA for confirmation of Japanese encephalitis infections in the Philippines. Southeast Asian J. Trop. Med. Public Health 37 Suppl 3, 136–139.
19. Olsen, S.J., Supawat, K., Campbell, A.P., Anantapreecha, S., Liamsuwan, S., Tunlayadechanont, S., Visudtibhan, A., Lupthikulthum, S., Dhiravibulya, K., Viriyavejakul, A., Vasiknanonte, P., Rajborirug, K., Watanaveeradej, V., Nabangchang, C., Laven, J., Kosoy, O., Panella, A., Ellis, C., Henchaichon, S., Khetsuriani, N., Powers, A.M., Dowell, S.F., Fischer, M., 2010. Japanese encephalitis virus remains an important cause of encephalitis in Thailand. International Journal of Infectious Diseases 14, e888–e892. https://doi.org/10.1016/j.ijid.2010.03.022
20. Ompusunggu, S., Hills, S.L., Maha, M.S., Moniaga, V.A., Susilarini, N.K., Widjaya, A., Sasmito, A., Suwandono, A., Sedyaningsih, E.R., Jacobson, J.A., 2008. Confirmation of Japanese encephalitis as an endemic human disease through sentinel surveillance in Indonesia. Am. J. Trop. Med. Hyg. 79, 963–970.
21. Pant, S.D., 2009. Epidemiology of Japanese encephalitis in Nepal. Journal of Nepal Paediatric Society 29, 35–37.
22. Partridge, J., Ghimire, P., Sedai, T., Bista, M.B., Banerjee, M., 2007. Endemic Japanese Encephalitis in the Kathmandu Valley, Nepal. The American Journal of Tropical Medicine and Hygiene 77, 1146–1149. https://doi.org/10.4269/ajtmh.2007.77.1146
23. Paul, R.C., Rahman, M., Gurley, E.S., Hossain, M.J., Diorditsa, S., Hasan, A.M., Banu, S.S., Alamgir, A.S.M., Rahman, M.A., Sandhu, H., Fischer, M., Luby, S.P., 2011. A Novel Low-Cost Approach to Estimate the Incidence of Japanese Encephalitis in the Catchment Area of Three Hospitals in Bangladesh. The American Journal of Tropical Medicine and Hygiene 85, 379–385. https://doi.org/10.4269/ajtmh.2011.10-0706
24. Phukan, A.C., Borah, P.K., Mahanta, J., 2004. Japanese encephalitis in Assam, northeast India. Southeast Asian J. Trop. Med. Public Health 35, 618–622.
25. Quan, T.M., Thao, T.T.N., Duy, N.M., Nhat, T.M., Clapham, H., 2020. Estimates of the global burden of Japanese encephalitis and the impact of vaccination from 2000-2015. eLife 9, e51027. https://doi.org/10.7554/eLife.51027
26. Ranjan, P., Gore, M., Selvaraju, S., Kushwaha, K.P., Srivastava, D.K., Murhekar, M., 2014. Changes in acute encephalitis syndrome incidence after introduction of Japanese encephalitis vaccine in a region of India. Journal of Infection 69, 200–202. https://doi.org/10.1016/j.jinf.2014.03.013
27. Sohn, Y.M., 2000. Japanese Encephalitis Immunization in South Korea: Past, Present, and Future. Emerging Infectious Diseases 6, 8.
28. Touch, S., Hills, S., Sokhal, B., Samnang, C., Sovann, L., Khieu, V., Soeung, S.C., Toda, K., Robinson, J., Grundy, J., 2009. Epidemiology and burden of disease from Japanese encephalitis in Cambodia: results from two years of sentinel surveillance. Tropical Medicine & International Health 14, 1365–1373. https://doi.org/10.1111/j.1365-3156.2009.02380.x
29. Wang, H., Li, Y., Liang, X., Liang, G., 2009. Japanese encephalitis in mainland china. Jpn. J. Infect. Dis. 62, 331–336.
30. WHO, 2020. WHO World Health Organization: Immunization, Vaccines And Biologicals. Vaccine preventable diseases Vaccines monitoring system 2020 Global Summary Reference Time Series: JAPENC [WWW Document]. URL https://apps.who.int/immunization_monitoring/globalsummary/timeseries/tscoveragejapenc.html (accessed 1.5.21).
31. Wierzba, T.F., Ghimire, P., Malla, S., Banerjee, M.K., Shrestha, S., Khanal, B., Sedai, T.R., Gibbons, R.V., 2008. Laboratory-based Japanese Encephalitis Surveillance in Nepal and the Implications for a

National Immunization Strategy. The American Journal of Tropical Medicine and Hygiene 78, 1002–1006. https://doi.org/10.4269/ajtmh.2008.78.1002
32. Wong, S.C., Ooi, M.H., Abdullah, A.R., Wong, S.Y., Krishnan, S., Tio, P.H., Pek, P.C., Lai, B.F., Mohan, A., Muhi, J., Kiyu, A., Arif, M.T., Cardosa, M.J., 2008. A decade of Japanese encephalitis surveillance in Sarawak, Malaysia: 1997–2006. Tropical Medicine & International Health 13, 52–55. https://doi.org/10.1111/j.1365-3156.2007.01967.x
33. Yen, N.T., Duffy, M.R., Hong, N.M., Hien, N.T., Fischer, M., Hills, S.L., 2010. Surveillance for Japanese Encephalitis in Vietnam, 1998–2007. The American Journal of Tropical Medicine and Hygiene 83, 816–819. https://doi.org/10.4269/ajtmh.2010.10-0262
