# Supplementary material for: The current burden of Japanese encephalitis and the estimated impacts of vaccination: Combining estimates of the spatial distribution and transmission intensity of a zoonotic pathogen
Source: PLoS Negl Trop Dis. 2021 Oct 13;15(10):e0009385. doi: 10.1371/journal.pntd.0009385 (PMC8544850; doi:10.1371/journal.pntd.0009385)
Supplement: S2 Table — (PDF) [file pntd.0009385.s002.pdf]

Table S2. Summary of vaccination coverage data used to estimate the burden of Japanese encephalitis (JE) from 2010-2019 for each country. Countries without details were assumed to have a vaccination coverage of 0.

Country	Start	End	Source
Bangladesh			
Bhutan			
Brunei			
Cambodia	2013	2019	[10], [12], [14]
China	2008	2019	[14]
East Timor			
India	2006	2019	[10], [14]
Indonesia	2018	2019	[3], [14]
Japan	1976	2006	[10]
	2007	2019	[14]
Laos	2015	2016	[5]
Malaysia	2001	2019	[14]
Myanmar	2017	2019	[13], [14]
Nepal	2005	2019	[6], [7], [14]
North Korea	2009	2014	[4]
Pakistan			
Papua New Guinea			
Philippines			
Singapore			
South Korea	1963	1999	[8], [11]
	2000	2005	[10]
	2006	2019	[14]
Sri Lanka	1988	2008	[10]
	2009	2019	[14]
Taiwan	1968	2019	[2], [8]
Thailand	1990	2015	[9], [10]
	2016	2019	[14]
Vietnam	1997	2005	[1]
	2006	2019	[14]

References

1. Choisy, M., 2017. Vaccine coverages from the WHO EPI in Vietnam [WWW Document]. URL https://github.com/choisy/epiVN (accessed 12.7.20).
2. Hsu, L.-C., Chen, Y.-J., Hsu, F.-K., Huang, J.-H., Chang, C.-M., Chou, P., Lin, I.-F., Chang, F.-Y., 2014. The Incidence of Japanese Encephalitis in Taiwan—A Population-Based Study. PLoS Negl Trop Dis 8. https://doi.org/10.1371/journal.pntd.0003030
3. Im, J., Balasubramanian, R., Yastini, N.W., Suwarba, I.G.N., Andayani, A.R., Bura, V., Jeon, H.J., Clemens, J.D., Marks, F., 2018. Protecting children against Japanese encephalitis in Bali, Indonesia. The Lancet 391, 2500–2501. https://doi.org/10.1016/S0140-6736(18)31191-7
4. Marks, F., Nyambat, B., Xu, Z.-Y., von Kalckreuth, V., Kilgore, P.E., Seo, H.J., Du, Y., Park, S.E., Im, J., Konings, F., Meyer, C.G., Wierzba, T.F., Clemens, J.D., 2015. Vaccine introduction in the Democratic People's Republic of Korea. Vaccine 33, 2297–2300. https://doi.org/10.1016/j.vaccine.2015.03.005
5. Ministry of Health, Laos. 2016. Japanese encephalitis SIA 2015 technical report.
6. Ministry of Health, Nepal. 2016. Japanese encephalitis vaccination campaign, Nepal. Technical Report. WHO-UNICEF Joint Reporting.
7. Mithila Public Health Services, Nepal. 2016. Coverage survey of Japanese encephalitis vaccine mass vaccination campaign in selected districts of Nepal.
8. Monath, T.P., 2002. Japanese Encephalitis Vaccines: Current Vaccines and Future Prospects, in: Mackenzie, J.S., Barrett, A.D.T., Deubel, V. (Eds.), Japanese Encephalitis and West Nile Viruses, Current Topics in Microbiology and Immunology. Springer, Berlin, Heidelberg, pp. 105–138. https://doi.org/10.1007/978-3-642-59403-8_6
9. Olsen, S.J., Supawat, K., Campbell, A.P., Anantapreecha, S., Liamsuwan, S., Tunlayadechanont, S., Visudtibhan, A., Lupthikulthum, S., Dhiravibulya, K., Viriyavejakul, A., Vasiknanonte, P., Rajborirug, K., Watanaveeradej, V., Nabangchang, C., Laven, J., Kosoy, O., Panella, A., Ellis, C., Henchaichon, S., Khetsuriani, N., Powers, A.M., Dowell, S.F., Fischer, M., 2010. Japanese encephalitis virus remains an important cause of encephalitis in Thailand. International Journal of Infectious Diseases 14, e888–e892. https://doi.org/10.1016/j.ijid.2010.03.022
10. Quan, T.M., Thao, T.T.N., Duy, N.M., Nhat, T.M., Clapham, H., 2020. Estimates of the global burden of Japanese encephalitis and the impact of vaccination from 2000-2015. eLife 9, e51027. https://doi.org/10.7554/eLife.51027
11. Sohn, Y.M., 2000. Japanese Encephalitis Immunization in South Korea: Past, Present, and Future. Emerging Infectious Diseases 6, 8.
12. WHO, 2016. Global Immunization News. March, 2016. URL: https://www.who.int/immunization/GIN_March_2016.pdf (accessed 4.6.21)
13. WHO, 2018. Japanese encephalitis vaccination campaign achievement [WWW Document]. URL: https://www.who.int/myanmar/our-work/japanese-encephalitis-vaccination-campaign-achievement (accessed 4.6.21).
14. WHO, 2020. WHO World Health Organization: Immunization, Vaccines And Biologicals. Vaccine preventable diseases Vaccines monitoring system 2020 Global Summary Reference Time Series: JAPENC [WWW Document]. URL: https://apps.who.int/immunization_monitoring/globalsummary/timeseries/tscoveragejapenc.html (accessed 1.5.21).
