# Supplementary material for: The current burden of Japanese encephalitis and the estimated impacts of vaccination: Combining estimates of the spatial distribution and transmission intensity of a zoonotic pathogen
Source: PLoS Negl Trop Dis. 2021 Oct 13;15(10):e0009385. doi: 10.1371/journal.pntd.0009385 (PMC8544850; doi:10.1371/journal.pntd.0009385)
Supplement: S3 Table — (PDF) [file pntd.0009385.s003.pdf]

Table S3. Mean annual number of JE cases (C) and deaths (D) per country from 2010 to 2019 under the vaccination and no vaccination scenarios. Numbers are presented for three different estimates of the at-risk population size - baseline, low, and high. See Methods for description of how these population sizes were estimated.

		Vaccination			No Vaccination		
Country		Baseline (95% CI)	Low (95% CI)	High (95% CI)	Baseline (95% CI)	Low (95% CI)	High (95% CI)
Bangladesh	C	10207 (3187 - 33585)	10152 (3176 - 33411)	12000 (3755 - 39511)	10207 (3193 - 33477)	10152 (3184 - 33391)	12001 (3764 - 39333)
	D	3711 (398 - 14267)	3691 (399 - 14277)	4363 (472 - 16732)	3711 (397 - 14170)	3691 (402 - 14242)	4365 (471 - 16765)
Bhutan	C	10 (1 - 32)	2 (0 - 7)	23 (5 - 73)	10 (1 - 32)	2 (0 - 7)	23 (5 - 76)
	D	4 (0 - 14)	1 (0 - 4)	8 (0 - 31)	4 (0 - 15)	1 (0 - 4)	8 (0 - 32)
Brunei	C	13 (2 - 44)	5 (0 - 18)	18 (4 - 59)	13 (2 - 43)	5 (0 - 17)	18 (4 - 61)
	D	5 (0 - 19)	2 (0 - 8)	7 (0 - 26)	5 (0 - 19)	2 (0 - 8)	7 (0 - 27)
Cambodia	C	509 (89 - 1714)	472 (83 - 1594)	721 (129 - 2414)	705 (221 - 2360)	653 (200 - 2178)	998 (311 - 3373)
	D	185 (13 - 758)	172 (12 - 711)	262 (19 - 1075)	257 (26 - 957)	238 (24 - 894)	363 (38 - 1362)
China	C	13958 (1959 - 48634)	13855 (1940 - 48066)	15796 (2220 - 54883)	34432 (10769 - 114096)	34180 (10697 - 113704)	38978 (12178 - 129234)
	D	5027 (329 - 20587)	4991 (325 - 20400)	5690 (371 - 23185)	12517 (1363 - 47941)	12423 (1331 - 47539)	14169 (1534 - 54947)
India	C	25470 (7864 - 82413)	13974 (4296 - 45199)	43783 (13511 - 141331)	30021 (9263 - 100390)	16471 (5083 - 55121)	51621 (15985 - 172686)
	D	9265 (1007 - 35177)	5082 (549 - 19278)	15926 (1720 - 60441)	10923 (1181 - 42815)	5992 (642 - 23454)	18782 (2031 - 73583)
Indonesia	C	7081 (2204 - 23423)	4941 (1544 - 16338)	13402 (4170 - 44423)	7098 (2209 - 23433)	4952 (1539 - 16330)	13432 (4182 - 44362)
	D	2575 (279 - 9925)	1797 (194 - 6940)	4876 (527 - 18768)	2582 (282 - 9865)	1802 (193 - 6933)	4887 (536 - 18801)
Japan	C	887 (239 - 2931)	855 (229 - 2823)	1088 (291 - 3610)	1270 (332 - 4229)	1224 (318 - 4052)	1557 (408 - 5173)
	D	323 (32 - 1251)	311 (31 - 1197)	396 (40 - 1528)	463 (47 - 1782)	446 (44 - 1705)	567 (58 - 2207)
Laos	C	167 (47 - 529)	147 (42 - 460)	241 (69 - 763)	172 (49 - 543)	151 (43 - 475)	248 (72 - 775)
	D	61 (6 - 229)	53 (5 - 201)	88 (8 - 329)	63 (5 - 235)	55 (5 - 206)	90 (9 - 337)
Malaysia	C	201 (21 - 804)	116 (12 - 470)	264 (29 - 1051)	1029 (322 - 3375)	594 (184 - 1962)	1349 (422 - 4449)
	D	74 (3 - 370)	43 (1 - 215)	97 (4 - 479)	374 (38 - 1429)	216 (22 - 833)	491 (52 - 1888)
Myanmar	C	1396 (210 - 4689)	1261 (189 - 4250)	1953 (293 - 6477)	1722 (517 - 5831)	1555 (466 - 5237)	2411 (729 - 8192)
	D	507 (35 - 2013)	457 (31 - 1803)	709 (50 - 2798)	625 (62 - 2306)	564 (57 - 2081)	874 (88 - 3231)
Nepal	C	768 (196 - 2460)	436 (112 - 1404)	947 (244 - 3031)	1266 (398 - 4187)	719 (224 - 2368)	1562 (494 - 5149)
	D	279 (27 - 1054)	159 (14 - 599)	345 (33 - 1294)	461 (49 - 1735)	262 (27 - 989)	568 (60 - 2155)
North Korea	C	412 (117 - 1300)	406 (116 - 1285)	449 (128 - 1428)	578 (174 - 1927)	570 (172 - 1905)	631 (190 - 2117)
	D	150 (15 - 571)	148 (14 - 570)	164 (16 - 626)	210 (21 - 822)	207 (22 - 805)	230 (24 - 888)
Pakistan	C	1360 (347 - 4476)	530 (135 - 1748)	7393 (1899 - 24421)	1359 (345 - 4466)	530 (134 - 1755)	7393 (1906 - 24352)
	D	495 (47 - 1970)	193 (17 - 760)	2691 (258 - 10633)	495 (47 - 1957)	193 (18 - 776)	2692 (262 - 10615)
Papua New	C	154 (36 - 513)	1 (0 - 5)	335 (83 - 1148)	154 (36 - 516)	1 (0 - 5)	335 (81 - 1128)
Guinea	D	56 (5 - 219)	0 (0 - 3)	122 (11 - 478)	56 (5 - 217)	0 (0 - 3)	122 (11 - 478)
Philippines	C	3899 (1199 - 12450)	3715 (1148 - 11788)	5290 (1630 - 16832)	3900 (1211 - 12438)	3715 (1145 - 11830)	5292 (1628 - 16794)
	D	1422 (149 - 5508)	1355 (140 - 5244)	1930 (201 - 7463)	1423 (147 - 5533)	1355 (139 - 5278)	1930 (200 - 7501)
Singapore	C	68 (18 - 215)	65 (17 - 207)	106 (29 - 334)	68 (18 - 213)	65 (17 - 205)	106 (29 - 337)
	D	25 (2 - 98)	24 (2 - 96)	39 (3 - 151)	25 (2 - 99)	24 (2 - 94)	39 (3 - 153)
South Korea	C	207 (56 - 675)	189 (51 - 615)	242 (66 - 776)	917 (282 - 2974)	839 (260 - 2760)	1072 (331 - 3526)

	D	75	(7 - 286)	69	(6 - 260)	88	(8 - 331)	334	(35 - 1271)	305	(32 - 1151)	389	(41 - 1466)
Sri Lanka	C	34	(8 - 105)	12	(2 - 39)	152	(44 - 482)	129	(38 - 427)	46	(13 - 148)	582	(180 - 1921)
	D	12	(0 - 47)	4	(0 - 18)	55	(5 - 208)	47	(4 - 183)	17	(1 - 65)	212	(21 - 822)
Taiwan	C	19	(4 - 60)	19	(4 - 60)	44	(11 - 140)	217	(66 - 706)	215	(66 - 701)	504	(156 - 1662)
	D	7	(0 - 27)	7	(0 - 27)	16	(1 - 62)	79	(8 - 303)	78	(8 - 303)	183	(19 - 713)
Thailand	C	356	(88 - 1181)	331	(82 - 1096)	704	(175 - 2339)	1165	(363 - 3843)	1082	(337 - 3565)	2301	(723 - 7589)
	D	130	(11 - 537)	121	(11 - 502)	257	(23 - 1064)	424	(44 - 1624)	393	(41 - 1525)	837	(91 - 3231)
Timor-Leste	C	7	(1 - 22)	5	(0 - 17)	37	(8 - 118)	7	(0 - 22)	5	(0 - 17)	37	(8 - 119)
	D	2	(0 - 11)	2	(0 - 8)	13	(0 - 54)	2	(0 - 10)	2	(0 - 8)	14	(0 - 53)
Vietnam	C	1726	(408 - 5622)	1706	(406 - 5537)	2171	(520 - 7048)	3950	(1241 - 12994)	3905	(1222 - 12860)	4966	(1554 - 16410)
	D	628	(58 - 2423)	621	(55 - 2395)	790	(71 - 3050)	1437	(155 - 5505)	1420	(153 - 5497)	1805	(197 - 6975)
