# Supplementary material for: The current burden of Japanese encephalitis and the estimated impacts of vaccination: Combining estimates of the spatial distribution and transmission intensity of a zoonotic pathogen
Source: PLoS Negl Trop Dis. 2021 Oct 13;15(10):e0009385. doi: 10.1371/journal.pntd.0009385 (PMC8544850; doi:10.1371/journal.pntd.0009385)
Supplement: S4 Table — (PDF) [file pntd.0009385.s004.pdf]

Table S4. Locations of documented Japanese encephalitis (JE) occurrence from 2000 to 2019. Latitude and longitude were estimated from the location name provided using Google Maps.

Country	ISO_L1	ISO_L2	Location	Lat	Long	Study period	Reference
HKG	HK-9		Tin Shui Wai, Yuen Long District	22.461	114.003	5/10/2017 - 5/10/2017	[3]
IND	IN-TG	Karimnagar	Uppal	18.182	79.488	7/1/2003 - 7/31/2003	[4]
IND	IN-TG	Warangal	Medipathi	18.054	79.452	7/1/2003 - 7/31/2003	[4]
IND	IN-HR	Ambala	Machonda	30.306	76.833	9/15/2003 - 11/30/2003	[12]
IND	IN-HR	Ambala	Hari Nagar	30.353	76.820	9/15/2003 - 11/30/2003	[12]
IND	IN-HR	Ambala	Dadupur	30.220	77.006	9/15/2003 - 11/30/2003	[12]
IND	IN-HR	Ambala	Dulyana	30.282	77.091	9/15/2003 - 11/30/2003	[12]
IND	IN-HR	Ambala	Manglai	30.309	76.908	9/15/2003 - 11/30/2003	[12]
IND	IN-HR	Ambala	Baldev Nagar	30.397	76.801	9/15/2003 - 11/30/2003	[12]
IND	IN-HR	Ambala	Dukhedi	30.282	76.881	9/15/2003 - 11/30/2003	[12]
IND	IN-HR	Ambala	Manmohan Nagar	30.397	76.779	9/15/2003 - 11/30/2003	[12]
IND	IN-HR	Ambala	Durana	30.231	76.801	9/15/2003 - 11/30/2003	[12]
IND	IN-HR	Ambala	Bullana	30.321	76.727	9/15/2003 - 11/30/2003	[12]
IND	IN-HR	Ambala	Mirzapur	30.278	76.774	9/15/2003 - 11/30/2003	[12]
IND	IN-AS	Dibrugarh	Barbaruah	27.402	94.881	1/1/2009 - 12/31/2010	[2]
IND	IN-AS	Dibrugarh	Lahoal	27.452	95.002	1/1/2009 - 12/31/2010	[2]
IND	IN-KL	Alapphuza	Muhamma	9.602	76.350	5/1/2011 - 5/31/2011	[16]
IND	IN-KL	Alapphuza	Pollathai	9.573	76.307	5/1/2011 - 5/31/2011	[16]
IND	IN-DL		JJ Colony, Bawana	28.807	77.053	9/15/2011 - 9/15/2011	[13]
IND	IN-KL	Alappuzha	Thycattussery	9.773	76.345	9/1/2012 - 10/31/2012	[1]
IND	IN-KL	Alappuzha	Pattanakkad	9.729	76.316	9/1/2012 - 10/31/2012	[1]
IND	IN-KL	Alappuzha	Kanjikuzhi	9.622	76.335	9/1/2012 - 10/31/2012	[1]
IND	IN-KL	Alappuzha	Aryad	9.545	76.347	9/1/2012 - 10/31/2012	[1]
IND	IN-KL	Alappuzha	Ambalapuzha	9.380	76.361	9/1/2012 - 10/31/2012	[1]
IND	IN-KL	Alappuzha	Alappuzha municipality	9.503	76.341	9/1/2012 - 10/31/2012	[1]
IND	IN-KL	Alappuzha	Chambakulam	9.407	76.404	9/1/2012 - 10/31/2012	[1]
IND	IN-KL	Alappuzha	Veliyanad	9.449	76.466	9/1/2012 - 10/31/2012	[1]
IND	IN-KL	Alappuzha	Chengannur	9.320	76.611	9/1/2012 - 10/31/2012	[1]
IND	IN-KL	Alappuzha	Haripad	9.284	76.448	9/1/2012 - 10/31/2012	[1]
IND	IN-KL	Alappuzha	Mavelikkara	9.244	76.538	9/1/2012 - 10/31/2012	[1]
IND	IN-KL	Alappuzha	Bharanikkavu	9.176	76.542	9/1/2012 - 10/31/2012	[1]
IND	IN-KL	Alappuzha	Muthukulam	9.215	76.458	9/1/2012 - 10/31/2012	[1]
IND	IN-OR	Malkangiri	Uskapalli	18.252	82.017	9/16/2012 - 11/2/2012	[5]
IND	IN-OR	Malkangiri	Charkiguda and Pradhaniguda	18.329	81.802	9/16/2012 - 11/2/2012	[5]
IND	IN-TN	Thanjavur	Kovilur	10.588	79.249	2/1/2011 - 10/31/2013	[14]
IND	IN-TN	Thanjavur	Naduvakottai	10.430	79.261	2/1/2011 - 10/31/2013	[14]

IND	IN-TN	Thanjavur	Kakkaraikottai	10.601	79.216	2/1/2011 - 10/31/2013	[14]
IND	IN-TN	Thanjavur	Urantharayankudikadu	10.585	79.269	2/1/2011 - 10/31/2013	[14]
IND	IN-TN	Thanjavur	Thiruvidaimaruthur	10.997	79.454	2/1/2011 - 10/31/2013	[14]
IND	IN-TN	Thanjavur	Muthur	10.907	79.457	2/1/2011 - 10/31/2013	[14]
IND	IN-WB	Koch Bihar	Koch Bihar	26.344	89.451	6/1/2014 - 9/10/2014	[7]
IND	IN-WB	Alipurduar	Alipurduar	26.489	89.535	6/1/2014 - 9/10/2014	[7]
IND	IN-WB	Koch Bihar	Dinhata	26.131	89.466	6/1/2014 - 9/10/2014	[7]
IND	IN-WB	Koch Bihar	Bakshikhamar	26.035	89.531	6/1/2014 - 9/10/2014	[7]
IND	IN-WB	Koch Bihar	Tufanganj	26.318	89.658	6/1/2014 - 9/10/2014	[7]
IND	IN-WB	Alipurduar	Simlabari	26.599	89.691	6/1/2014 - 9/10/2014	[7]
IND	IN-WB	Koch Bihar	Sitalkuchi	26.169	89.181	6/1/2014 - 9/10/2014	[7]
IND	IN-WB	Koch Bihar	Nishiganj	26.314	89.302	6/1/2014 - 9/10/2014	[7]
IND	IN-WB	Koch Bihar	Mathabhanga	26.343	89.213	6/1/2014 - 9/10/2014	[7]
IND	IN-WB	Koch Bihar	Balasi	26.427	89.182	6/1/2014 - 9/10/2014	[7]
IND	IN-WB	Alipurduar	Silbarihat	26.498	89.344	6/1/2014 - 9/10/2014	[7]
IND	IN-WB	Japaiguri	Salbari	26.544	89.107	6/1/2014 - 9/10/2014	[7]
IND	IN-WB	Koch Bihar	Daribas	26.302	88.870	6/1/2014 - 9/10/2014	[7]
IND	IN-WB	Koch Bihar	Haldibari	26.333	88.780	6/1/2014 - 9/10/2014	[7]
IND	IN-WB	Japaiguri	Jalpaiguri	26.522	88.718	6/1/2014 - 9/10/2014	[7]
IND	IN-WB	Japaiguri	Maynaguri	26.575	88.822	6/1/2014 - 9/10/2014	[7]
IND	IN-WB	Japaiguri	Gairkata	26.704	89.024	6/1/2014 - 9/10/2014	[7]
IND	IN-WB	Japaiguri	Banarhat	26.792	89.030	6/1/2014 - 9/10/2014	[7]
IND	IN-WB	Japaiguri	Nagrakata	26.882	88.909	6/1/2014 - 9/10/2014	[7]
IND	IN-WB	Japaiguri	Mal Bazar	26.862	88.751	6/1/2014 - 9/10/2014	[7]
IND	IN-WB	Japaiguri	Fatakpur	26.567	88.537	6/1/2014 - 9/10/2014	[7]
IND	IN-WB	Darjiling	Darjeeling	27.041	88.266	6/1/2014 - 9/10/2014	[7]
IND	IN-WB	Darjiling	Khoribari	26.554	88.191	6/1/2014 - 9/10/2014	[7]
IND	IN-WB	Darjiling	Bagdogra	26.698	88.315	6/1/2014 - 9/10/2014	[7]
IND	IN-WB	Darjiling	Siliguri	26.728	88.394	6/1/2014 - 9/10/2014	[7]
IND	IN-WB	Darjiling	Belgachi	26.755	88.233	6/1/2014 - 9/10/2014	[7]
IND	IN-WB	Darjiling	Dudhia	26.820	88.245	6/1/2014 - 9/10/2014	[7]
IND	IN-WB	Uttar Dinajpur	Bhagabati	26.379	88.299	6/1/2014 - 9/10/2014	[7]
IND	IN-WB	Uttar Dinajpur	Lalbazar	26.397	88.432	6/1/2014 - 9/10/2014	[7]
IND	IN-WB	Uttar Dinajpur	Islampur	26.262	88.194	6/1/2014 - 9/10/2014	[7]
IND	IN-WB	Uttar Dinajpur	Raskora	26.002	88.043	6/1/2014 - 9/10/2014	[7]
IND	IN-WB	Uttar Dinajpur	Raiganj	25.611	88.134	6/1/2014 - 9/10/2014	[7]
IND	IN-WB	Uttar Dinajpur	Bangalbari	25.621	88.226	6/1/2014 - 9/10/2014	[7]
IND	IN-WB	Uttar Dinajpur	Kaliyaganj	25.641	88.331	6/1/2014 - 9/10/2014	[7]

IND	IN-WB	Dakshin Dinajpur	Balurghat	25.230	88.787	6/1/2014 - 9/10/2014	[7]
IND	IN-WB	Dakshin Dinajpur	Gangarampur	25.408	88.523	6/1/2014 - 9/10/2014	[7]
IND	IN-WB	Uttar Dinajpur	Mahindra	25.434	88.267	6/1/2014 - 9/10/2014	[7]
JPN	JP-34	Hatsukaichi	Otake	34.299	132.222	8/1/1999 - 10/31/2002	[10]
JPN	JP-34	Fukuyama	Fukuyama	34.492	133.334	8/1/1999 - 10/31/2002	[10]
JPN	JP-34	Shobara	Kuchiwacho Nagata	34.891	132.923	8/1/1999 - 10/31/2002	[10]
JPN	JP-34	Miyoshi	Mirasakacho Nika	34.788	132.971	8/1/1999 - 10/31/2002	[10]
KOR	KR-41		Pyeongtaek	36.992	127.113	9/25/2008 - 9/25/2008	[15]
KOR	KR-28		Namdomg	37.453	126.730	9/25/2008 - 9/25/2008	[15]
KOR	KR-47		Goryeong	35.733	128.274	10/13/2008 - 10/13/2008	[15]
KOR	KR-41		Yangju	37.783	127.043	9/20/2010 - 9/20/2010	[15]
KOR	KR-41		Yeoju	37.288	127.633	9/27/2010 - 9/27/2010	[15]
KOR	KR-42		Hwacheon	38.113	127.698	10/21/2010 - 10/21/2010	[15]
KOR	KR-41		Pyeongtaek	36.992	127.113	8/29/2012 - 8/29/2012	[15]
KOR	KR-41		Iansan	37.315	126.823	8/19/2013 - 8/19/2013	[15]
KOR	KR-41		Hwaseong	37.199	126.827	9/11/2014 - 9/11/2014	[15]
KOR	KR-27		Dalseong	35.780	128.428	9/22/2014 - 9/22/2014	[15]
KOR	KR-41		Suwon	37.265	127.028	9/8/2015 - 9/8/2015	[15]
KOR	KR-11		Yongsan	37.533	126.985	10/20/2016 - 10/20/2016	[15]
KOR	KR-41		Paju	37.758	126.778	10/29/2016 - 10/29/2016	[15]
KOR	KR-41	Dongducheon	Camp Casey Dongducheon	37.921	127.058	7/1/2008 - 10/31/2011	[6]
LAO	LA-VT		Vientiane	18.001	102.542	1/1/2006 - 3/31/2006	[17]
LAO	LA-VT		Vientiane	17.985	102.551	1/1/2006 - 3/31/2006	[17]
LAO	LA-VT		Vientiane	17.974	102.600	1/1/2006 - 3/31/2006	[17]
LAO	LA-VT		Vientiane	17.959	102.616	1/1/2006 - 3/31/2006	[17]
LAO	LA-VT		Vientiane	18.009	102.611	1/1/2006 - 3/31/2006	[17]
LAO	LA-VT		Vientiane	18.001	102.617	1/1/2006 - 3/31/2006	[17]
LAO	LA-VT		Vientiane	18.023	102.610	1/1/2006 - 3/31/2006	[17]
LAO	LA-VT		Vientiane	17.901	102.634	1/1/2006 - 3/31/2006	[17]
LAO	LA-VT		Vientiane	17.900	102.640	1/1/2006 - 3/31/2006	[17]
LAO	LA-KH		Thalang	17.844	105.053	5/1/2007 - 2/28/2008	[8]
LAO	LA-KH		SopPhene	17.836	105.049	5/1/2007 - 2/28/2008	[8]
LAO	LA-KH		Sophia	17.808	105.053	5/1/2007 - 2/28/2008	[8]
LAO	LA-KH		NonBouakham	17.820	105.048	5/1/2007 - 2/28/2008	[8]
LAO	LA-KH		NakaNeua	17.760	105.113	5/1/2007 - 2/28/2008	[8]
LAO	LA-KH		NakaiTai	17.773	105.089	5/1/2007 - 2/28/2008	[8]
LAO	LA-KH		Namnian	17.790	105.066	5/1/2007 - 2/28/2008	[8]
LAO	LA-KH		Oudomsouk	17.708	105.147	5/1/2007 - 2/28/2008	[8]
LAO	LA-KH		Phonphanpaek	17.703	105.170	5/1/2007 - 2/28/2008	[8]
MYS	MY-07	Timur Laut	George Town	5.416	100.319	1/1/2014 - 12/31/2014	[9]

MYS	MY-07	Seberang Perai Utara	Bagan Jermal	5.415	100.374	1/1/2014 - 12/31/2014	[9]
MYS	MY-07	Seberang Perai Utara	Tasek Gelugor	5.478	100.490	1/1/2014 - 12/31/2014	[9]
PNG	PG-MBA	Alotau	Alatau	-10.313	150.462	1/1/2000 - 12/31/2001	[11]

References

1. Balakrishnan, Anukumar, Romy Jose Thekkekare, Gajanan Sapkal, and Babasaheb V. Tandale. "Seroprevalence of Japanese Encephalitis Virus & West Nile Virus in Alappuzha District, Kerala." *The Indian Journal of Medical Research* 146, no. Suppl 1 (July 2017): S70–76. https://doi.org/10.4103/ijmr.IJMR_1638_15.

2. Borah, J., P. Dutta, S. A. Khan, and J. Mahanta. "Epidemiological Concordance of Japanese Encephalitis Virus Infection among Mosquito Vectors, Amplifying Hosts and Humans in India." *Epidemiology & Infection* 141, no. 1 (2013): 74–80.

3. Cheng, Vincent CC, Siddharth Sridhar, Shuk-Ching Wong, Sally CY Wong, Jasper FW Chan, Cyril CY Yip, Chi-Hung Chau, Timmy WK Au, Yu-Yan Hwang, and Carol SW Yau. "Japanese Encephalitis Virus Transmitted via Blood Transfusion, Hong Kong, China." *Emerging Infectious Diseases* 24, no. 1 (2018): 49.

4. Das, Bina Pani, Shiv Lal, and V. K. Saxena. "Outdoor Resting Preference of *Culex Tritaeniorhynchus*, the Vector of Japanese Encephalitis in Warangal and Karim Nagar Districts, Andhra Pradesh." *Journal of Vector Borne Diseases* 41, no. 1/2 (2004): 32.

5. Dwibedi, Bhagirathi, Namita Mohapatra, Sushil Kumar Rathore, Maheswar Panda, Satya Sundar Pati, Jyotsnamayee Sabat, Bandana Thakur, Sailendra Panda, and Shantanu Kumar Kar. "An Outbreak of Japanese Encephalitis after Two Decades in Odisha, India." *The Indian Journal of Medical Research* 142, no. Suppl 1 (2015): S30.

6. Eick-Cost, Angelia A., Zheng Hu, Terry A. Klein, Robert J. Putnak, and Richard G. Jarman. "Seroconversion to Japanese Encephalitis Virus among US Infantry Forces in Korea." *The American Journal of Tropical Medicine and Hygiene* 93, no. 5 (2015): 1052.

7. Gurav, Yogesh K., Vijay P. Bondre, Babasaheb V. Tandale, Rekha G. Damle, Sanjay Mallick, Uday S. Ghosh, and Shankha S. Nag. "A Large Outbreak of Japanese Encephalitis Predominantly among Adults in Northern Region of West Bengal, India." *Journal of Medical Virology* 88, no. 11 (2016): 2004–11.

8. Hiscox, Alexandra, Christian H. Winter, Phengta Vongphrachanh, Thongchanh Sisouk, Virasack Somoulay, Samlane Phompida, Surinder Kaul, Pany Sananikhom, Nguyen Thi Yen, and Richard E. Paul. "Serological Investigations of Flavivirus Prevalence in Khammouane Province, Lao People's Democratic Republic, 2007–2008." *The American Journal of Tropical Medicine and Hygiene* 83, no. 5 (2010): 1166.

9. Kumar, Kiven, Siti Suri Arshad, Gayathri Thevi Selvarajah, Jalila Abu, Ooi Peck Toung, Yusuf Abba, A. R. Yasmin, Faruku Bande, Reuben Sharma, and Bee Lee Ong. "Japanese Encephalitis in Malaysia: An Overview and Timeline." *Acta Tropica* 185 (2018): 219–29.

10. Kuwayama, Masaru, Mikako Ito, Shinichi Takao, Yukie Shimazu, Shinji Fukuda, Kazuo Miyazaki, Ichiro Kurane, and Tomohiko Takasaki. "Japanese Encephalitis Virus in Meningitis Patients, Japan." *Emerging Infectious Diseases* 11, no. 3 (March 2005): 471–73. https://doi.org/10.3201/eid1103.040285.

11. Mackenzie, J. S., K. B. Chua, P. W. Daniels, B. T. Eaton, H. E. Field, R. A. Hall, K. Halpin, C. A. Johansen, P. D. Kirkland, and S. K. Lam. "Emerging Viral Diseases of Southeast Asia and the Western Pacific." *Emerging Infectious Diseases* 7, no. 3 Suppl (2001): 497.

12. Rao, P., K. Joshi, B. Mishra, R. K. Ratho, and R. Kumar. "An Outbreak of Japanese Encephalitis in Haryana." *The Journal of Communicable Diseases* 37, no. 1 (March 2005): 78–81.

13. Routray, D., J. R. Sahoo, P. Singh, A. Agarwal, S. K. Jain, and S. V. Singh. "Public Health Response to the First Case of Japanese Encephalitis in Delhi, 2011." *The Journal of Communicable Diseases* 44, no. 3 (2012): 157–61.

14. Samuel, P. Philip, V. Thenmozhi, M. Muniaraj, D. Ramesh, S. Victor Jerald Leo, T. Balaji, K. Venkatasubramani, J. Nagaraj, and R. Paramasivan. "Changing Paradigm in the Epidemiology of Japanese Encephalitis in a Non-Endemic Region." *Journal of Vector Borne Diseases* 55, no. 3 (July 1, 2018): 203. https://doi.org/10.4103/0972-9062.249130.

15. Shin, Een-Suk, Ok Park, and In-Sik Kong. "Review of the Incidence of Japanese Encephalitis in Foreign-Born and Korean Nationals Living in the Republic of Korea, 2007–2016." *Osong Public Health and Research Perspectives* 9, no. 3 (2018): 126.

16. Thenmozhi, V., R. Paramasivan, P. Philip Samuel, T. Kamaraj, T. Balaji, K. J. Dhananjeyan, K. Venkatasubramani, and B. K. Tyagi. "Dual Infection in Human by Japanese Encephalitis Virus & Chikungunya Virus in Alappuzha District, Kerala, India." *The Indian Journal of Medical Research* 138, no. 3 (2013): 362.

17. Vallée, Julie, Audrey Dubot-Pérès, Phonepaseuth Ounaphom, Chantalay Sayavong, Juliet E. Bryant, and Jean-Paul Gonzalez. "Spatial Distribution and Risk Factors of Dengue and Japanese Encephalitis Virus Infection in Urban Settings: The Case of Vientiane, Lao PDR." *Tropical Medicine & International Health* 14, no. 9 (2009): 1134–42.
