# Supplementary figures and images for: The current burden of Japanese encephalitis and the estimated impacts of vaccination: Combining estimates of the spatial distribution and transmission intensity of a zoonotic pathogen
Source: PLoS Negl Trop Dis. 2021 Oct 13;15(10):e0009385. doi: 10.1371/journal.pntd.0009385 (PMC8544850; doi:10.1371/journal.pntd.0009385)

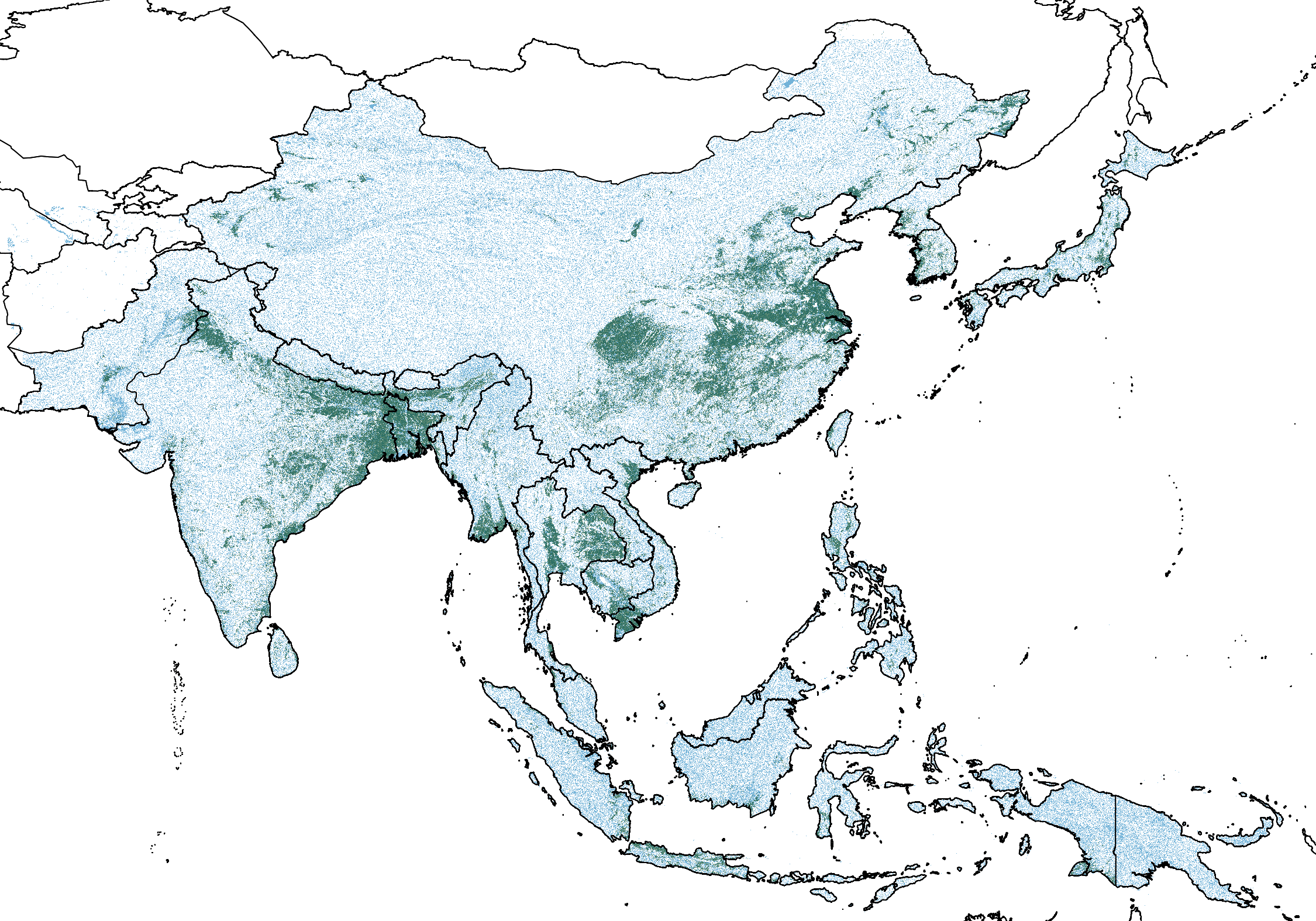

Supplement: S1 Fig — Rice cultivation map is derived from [9]. Extent of wetlands (including seasonal wetlands) based on wetlands types in tropical and sub-tropical Asia from [10]. The base map layer was generated using the geoBoundaries Comprehensive Global Administrative Zones (CGAZ) dataset available at https://github.com/wmgeolab/geoBoundaries/raw/main/releaseData/CGAZ/geoBoundariesCGAZ_ADM0.zip. (TIF) [file pntd.0009385.s005.tif]

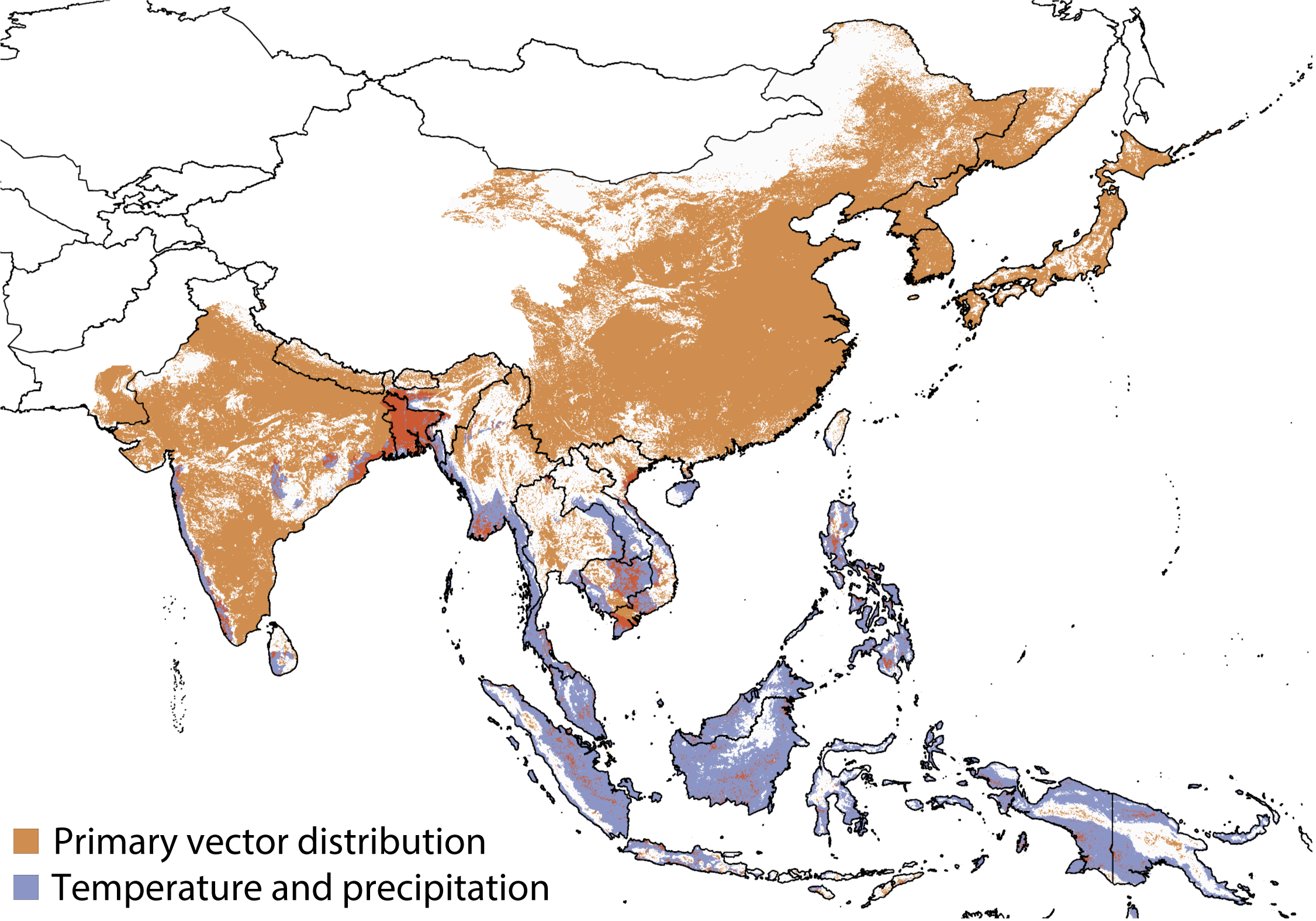

Supplement: S2 Fig — Orange represents areas where the modeled probability of Culex tritaeniorhynchus occurrence is ≥ 0.25 according to [15]. Blue represents regions with an annual mean temp of ≥ 20°C and ≥ 1500mm of annual precipitation. Darker orange areas represent regions where these two distributions overlap. The base map layer was generated using the geoBoundaries Comprehensive Global Administrative Zones (CGAZ) dataset available at https://github.com/wmgeolab/geoBoundaries/raw/main/releaseData/CGAZ/geoBoundariesCGAZ_ADM0.zip. (TIF) [file pntd.0009385.s006.tif]

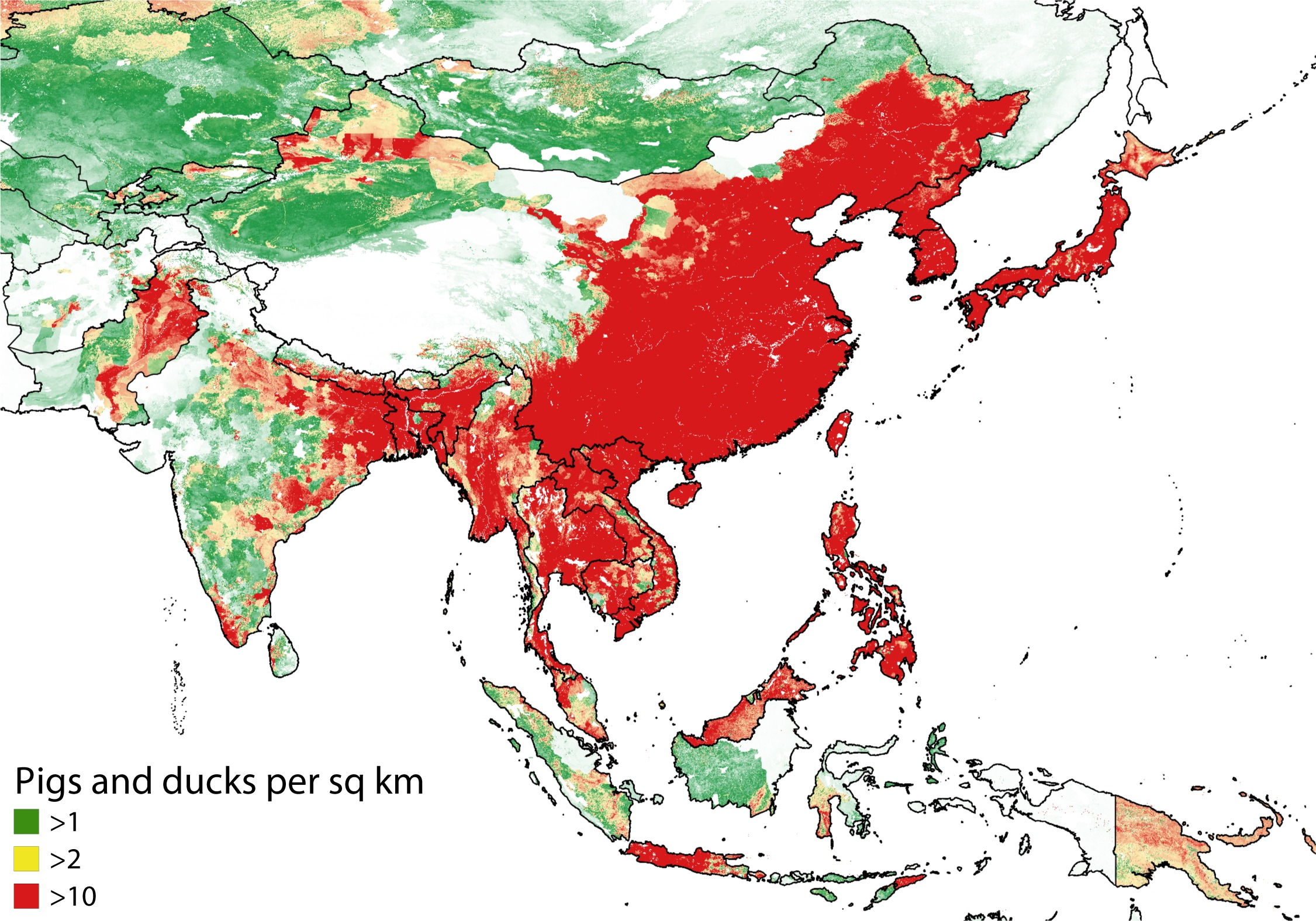

Supplement: S3 Fig — Map shows the three different thresholds used in estimating the size of the at-risk population in JE-endemic areas. The default threshold used for sustained JEV transmission was ≥ 2 per square km. Derived from [20]. The base map layer was generated using the geoBoundaries Comprehensive Global Administrative Zones (CGAZ) dataset available at https://github.com/wmgeolab/geoBoundaries/raw/main/releaseData/CGAZ/geoBoundariesCGAZ_ADM0.zip. (TIF) [file pntd.0009385.s007.tif]

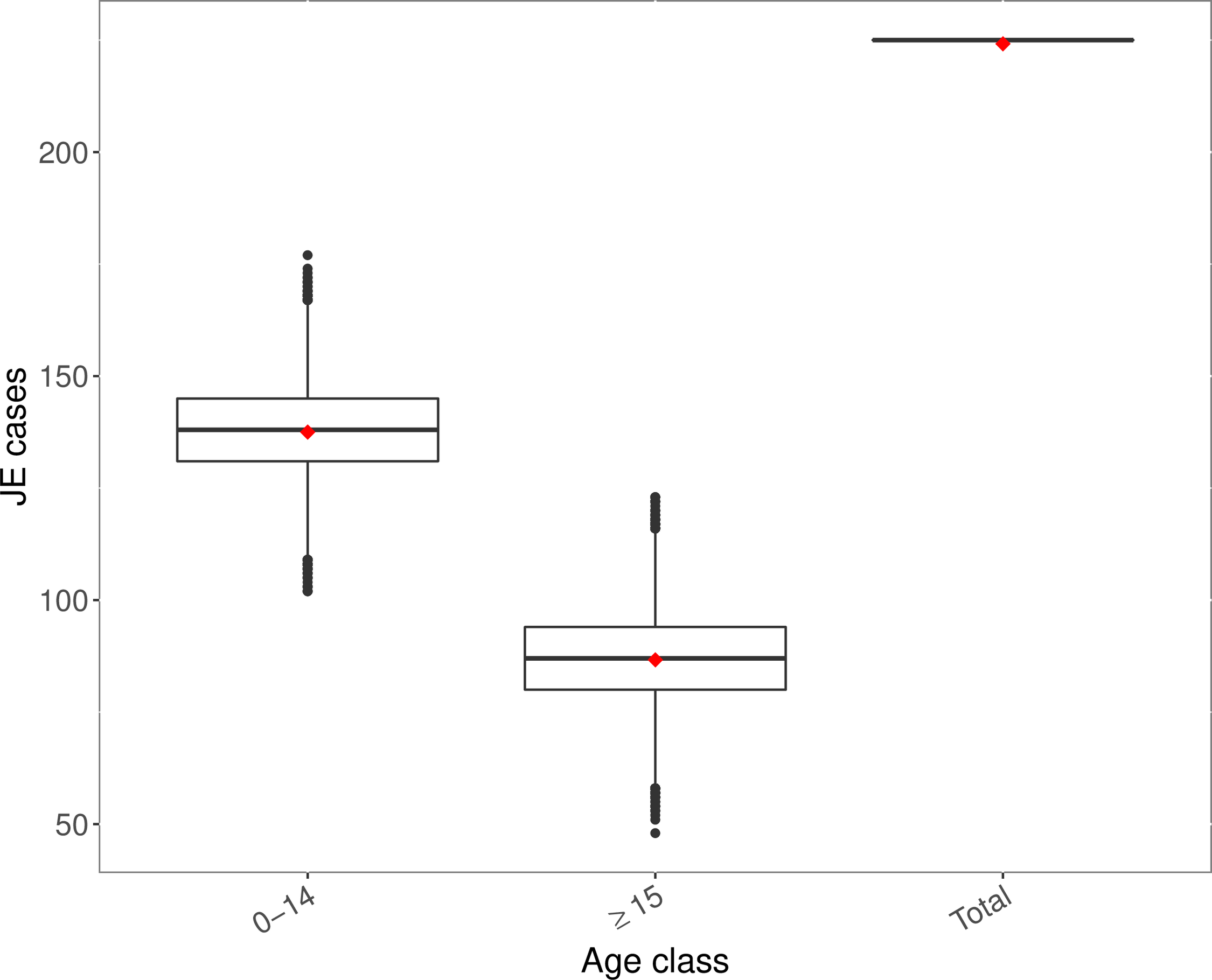

Supplement: S4 Fig — Boxplots represent predicted number of cases per age class based on draws from the joint posterior distribution of FOI and vaccination coverage (if included) estimates. Red diamonds represent the observed number of cases. (TIF) [file pntd.0009385.s008.tif]

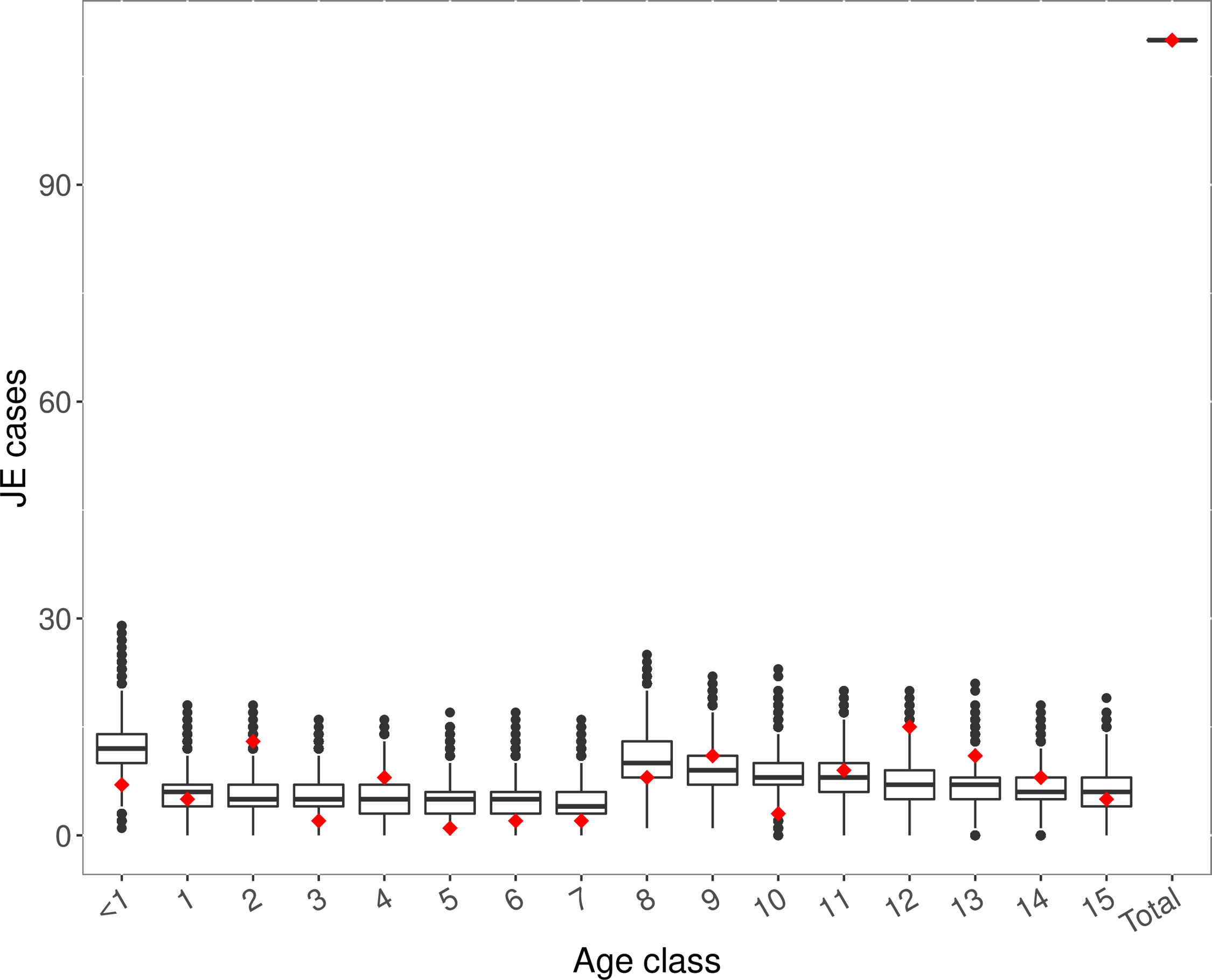

Supplement: S5 Fig — Boxplots represent predicted number of cases per age class based on draws from the joint posterior distribution of FOI and vaccination coverage (if included) estimates. Red diamonds represent the observed number of cases. (TIF) [file pntd.0009385.s009.tif]

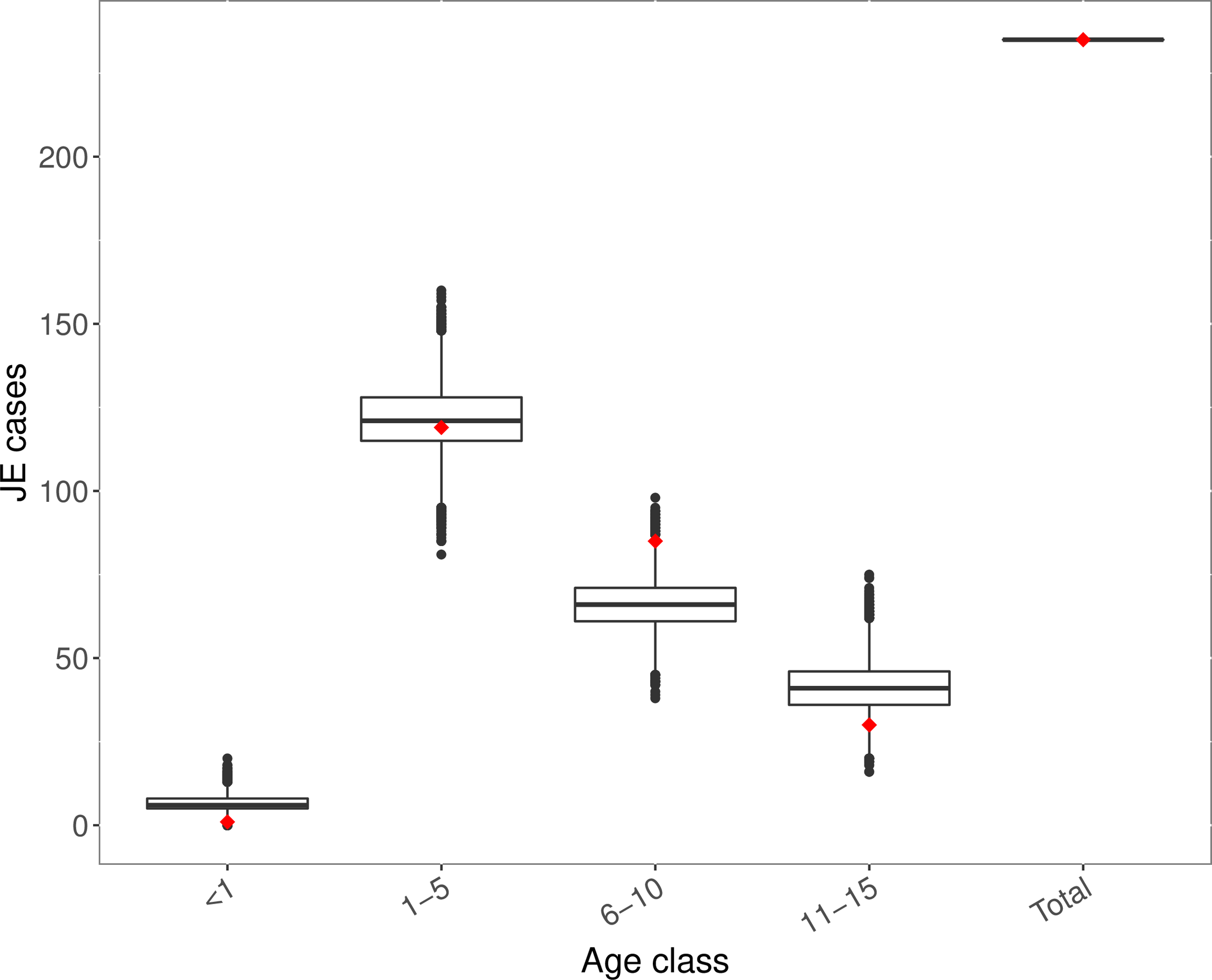

Supplement: S6 Fig — Boxplots represent predicted number of cases per age class based on draws from the joint posterior distribution of FOI and vaccination coverage (if included) estimates. Red diamonds represent the observed number of cases. (TIF) [file pntd.0009385.s010.tif]

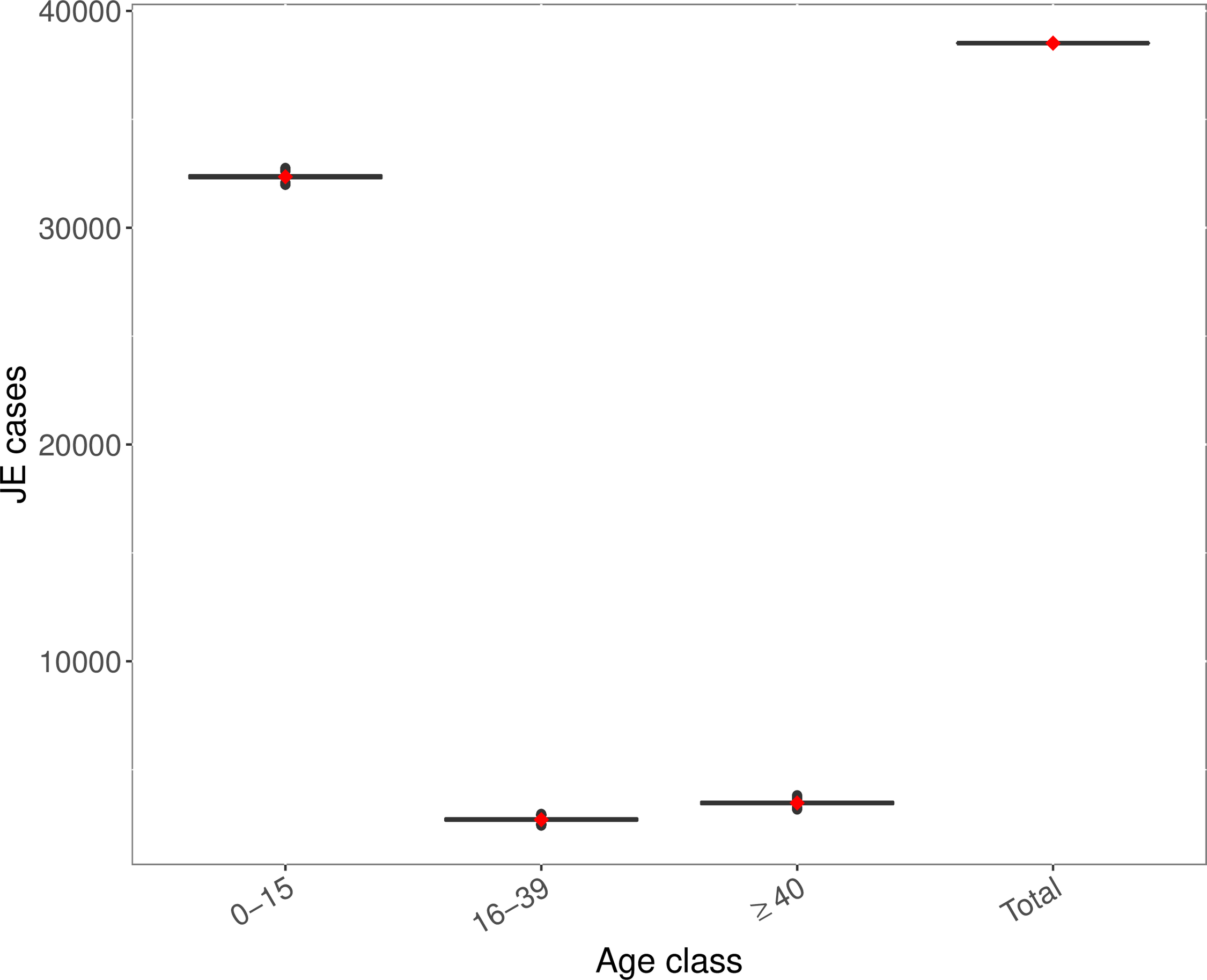

Supplement: S7 Fig — Boxplots represent predicted number of cases per age class based on draws from the joint posterior distribution of FOI and vaccination coverage (if included) estimates. Red diamonds represent the observed number of cases. (TIF) [file pntd.0009385.s011.tif]

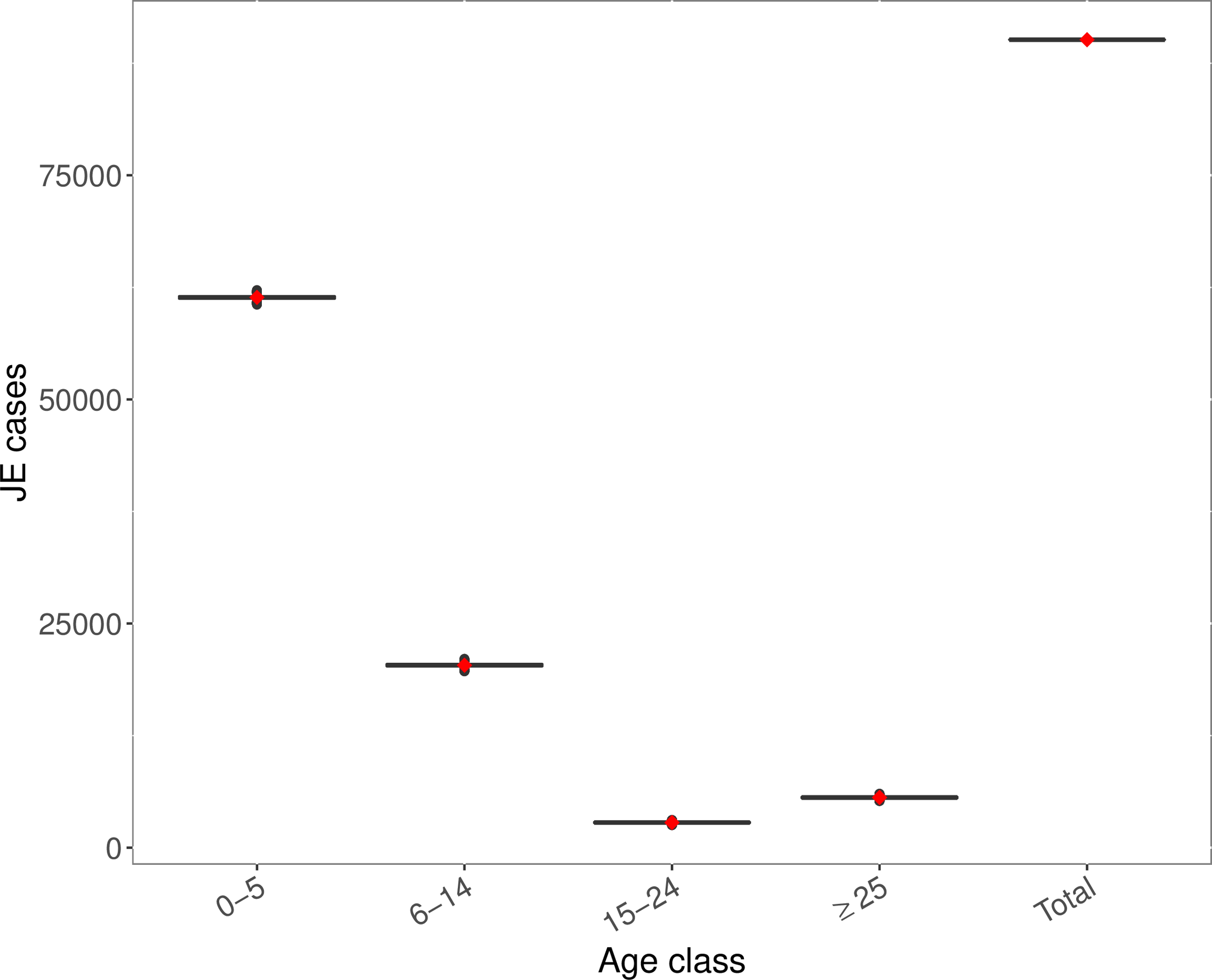

Supplement: S8 Fig — Boxplots represent predicted number of cases per age class based on draws from the joint posterior distribution of FOI and vaccination coverage (if included) estimates. Red diamonds represent the observed number of cases. (TIF) [file pntd.0009385.s012.tif]

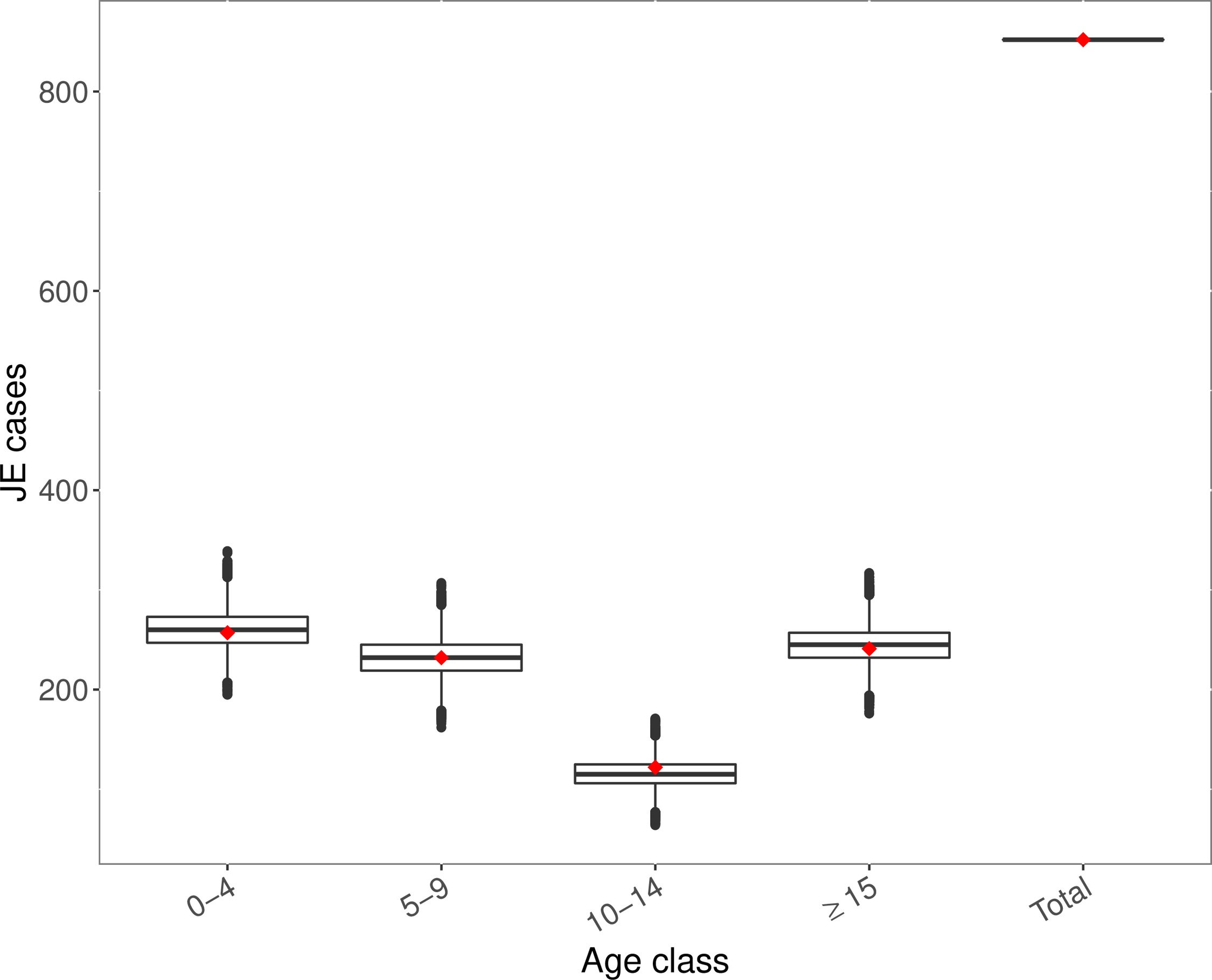

Supplement: S9 Fig — Boxplots represent predicted number of cases per age class based on draws from the joint posterior distribution of FOI and vaccination coverage (if included) estimates. Red diamonds represent the observed number of cases. (TIF) [file pntd.0009385.s013.tif]

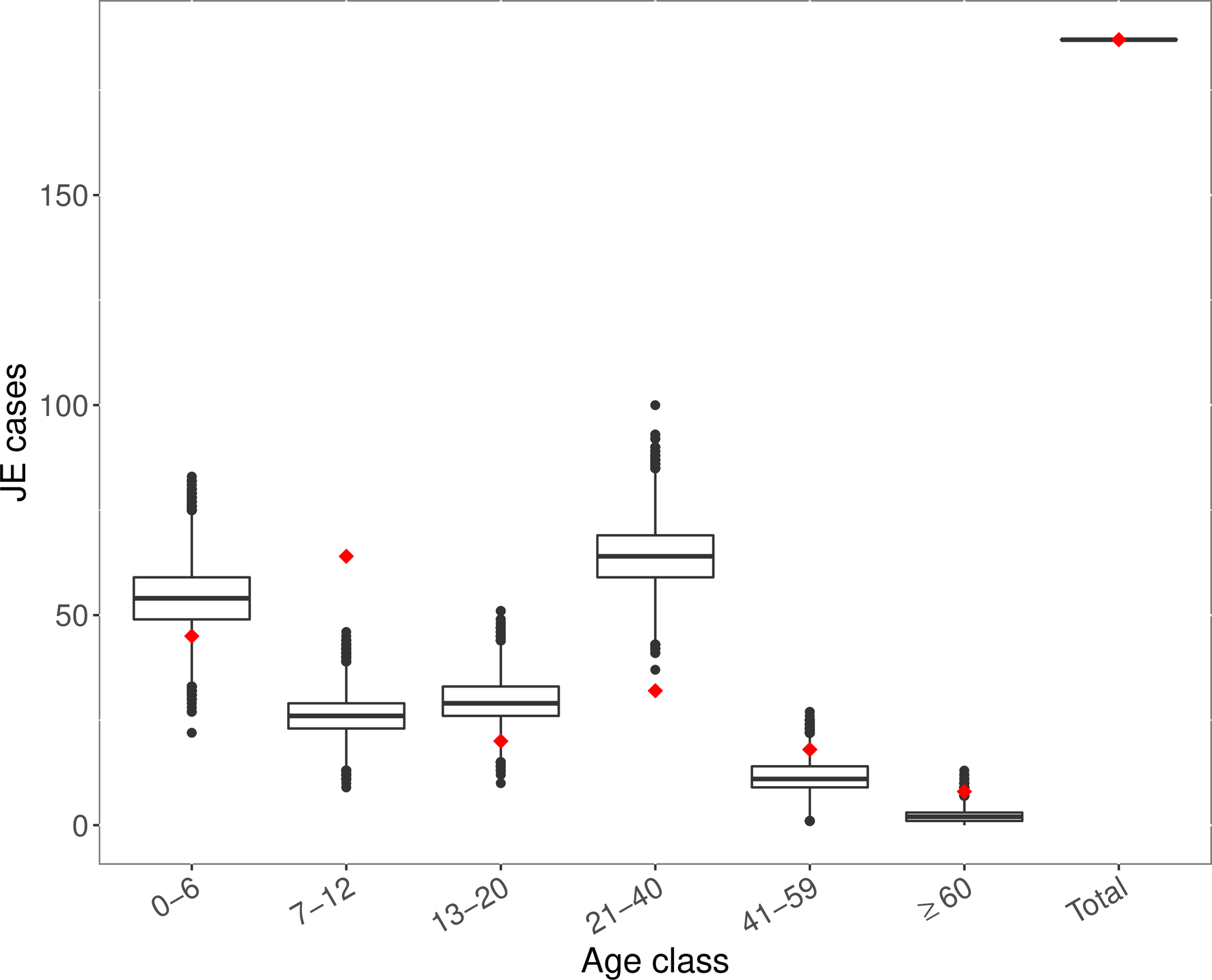

Supplement: S10 Fig — Boxplots represent predicted number of cases per age class based on draws from the joint posterior distribution of FOI and vaccination coverage (if included) estimates. Red diamonds represent the observed number of cases. (TIF) [file pntd.0009385.s014.tif]

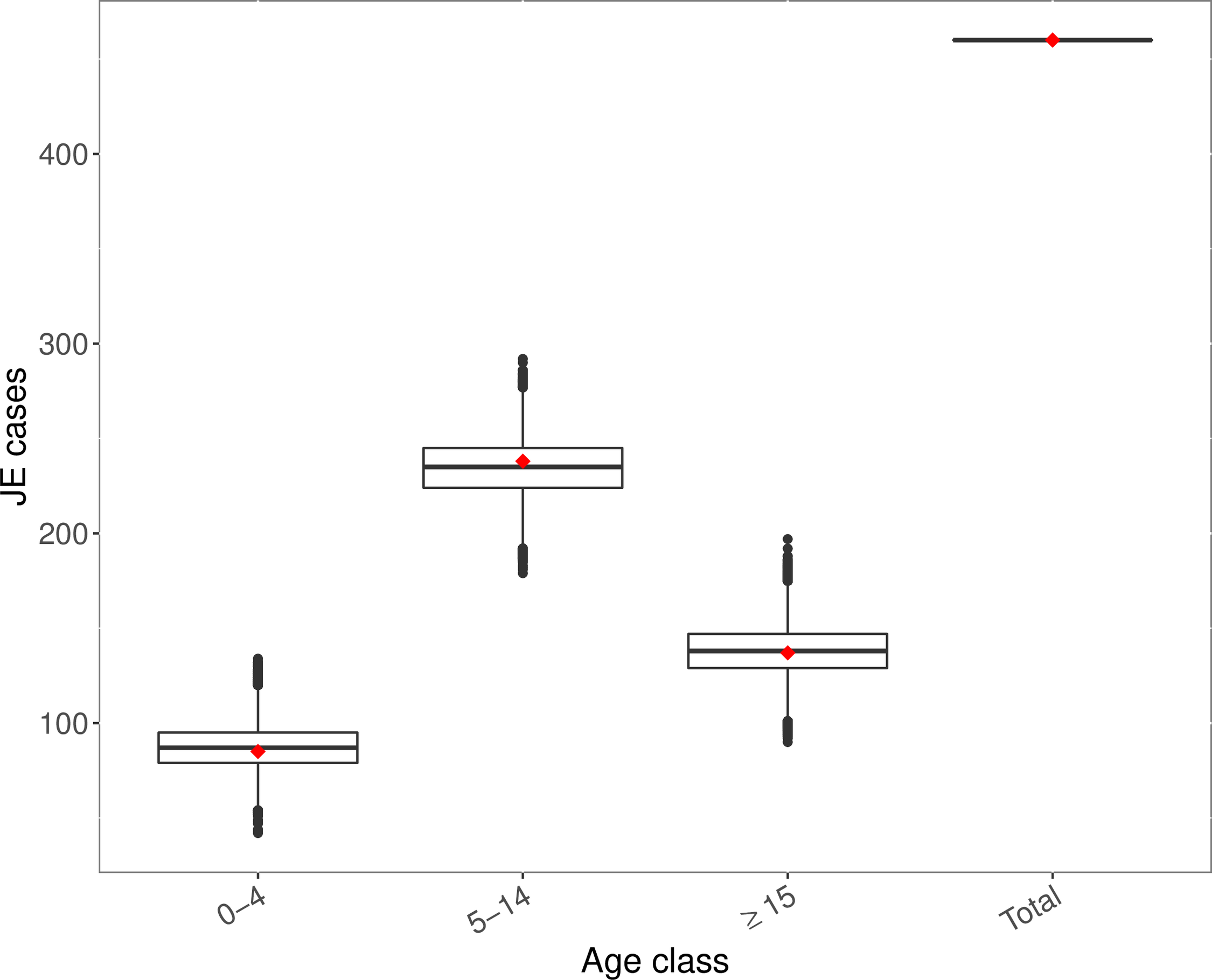

Supplement: S11 Fig — Boxplots represent predicted number of cases per age class based on draws from the joint posterior distribution of FOI and vaccination coverage (if included) estimates. Red diamonds represent the observed number of cases. (TIF) [file pntd.0009385.s015.tif]

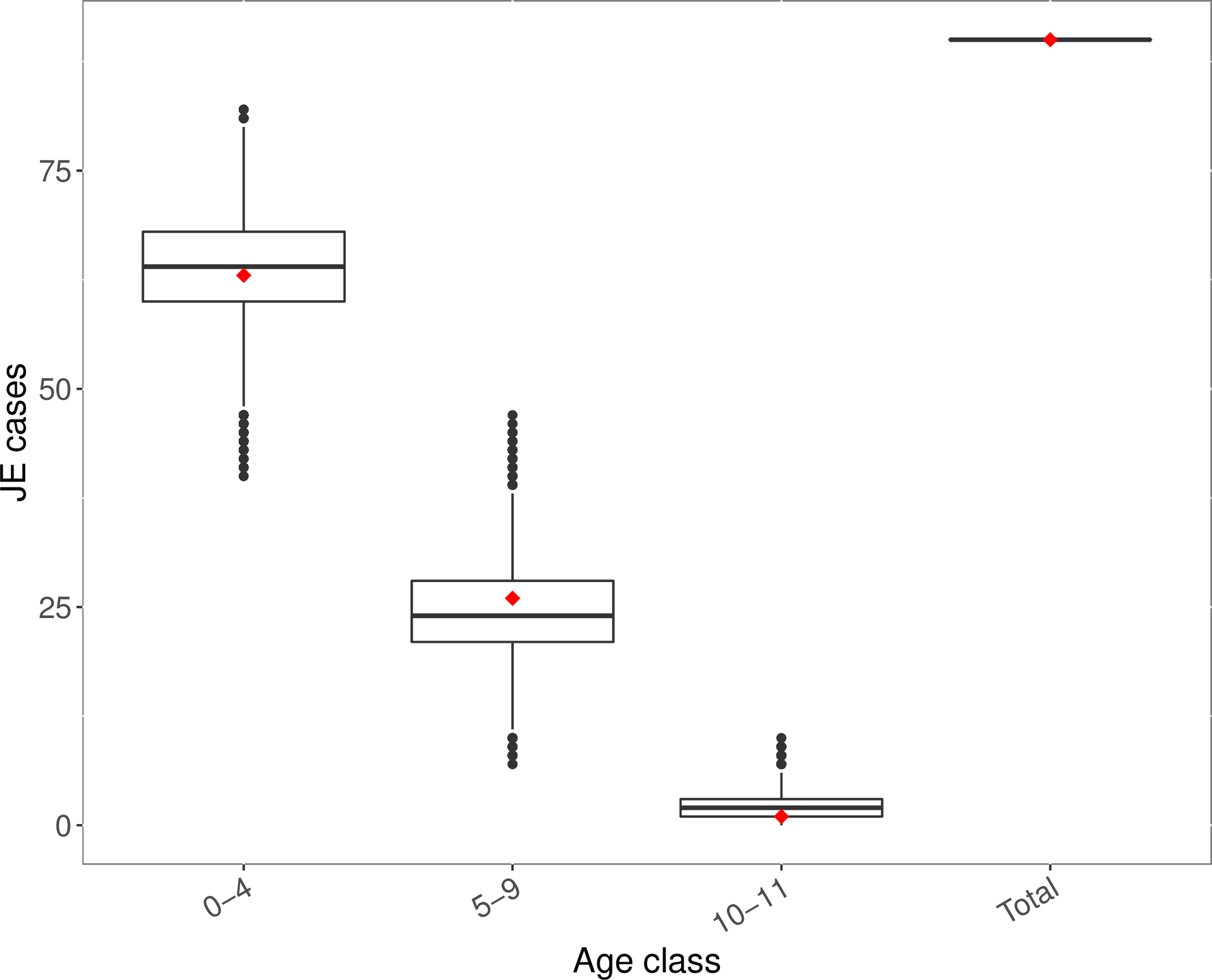

Supplement: S12 Fig — Boxplots represent predicted number of cases per age class based on draws from the joint posterior distribution of FOI and vaccination coverage (if included) estimates. Red diamonds represent the observed number of cases. (TIF) [file pntd.0009385.s016.tif]

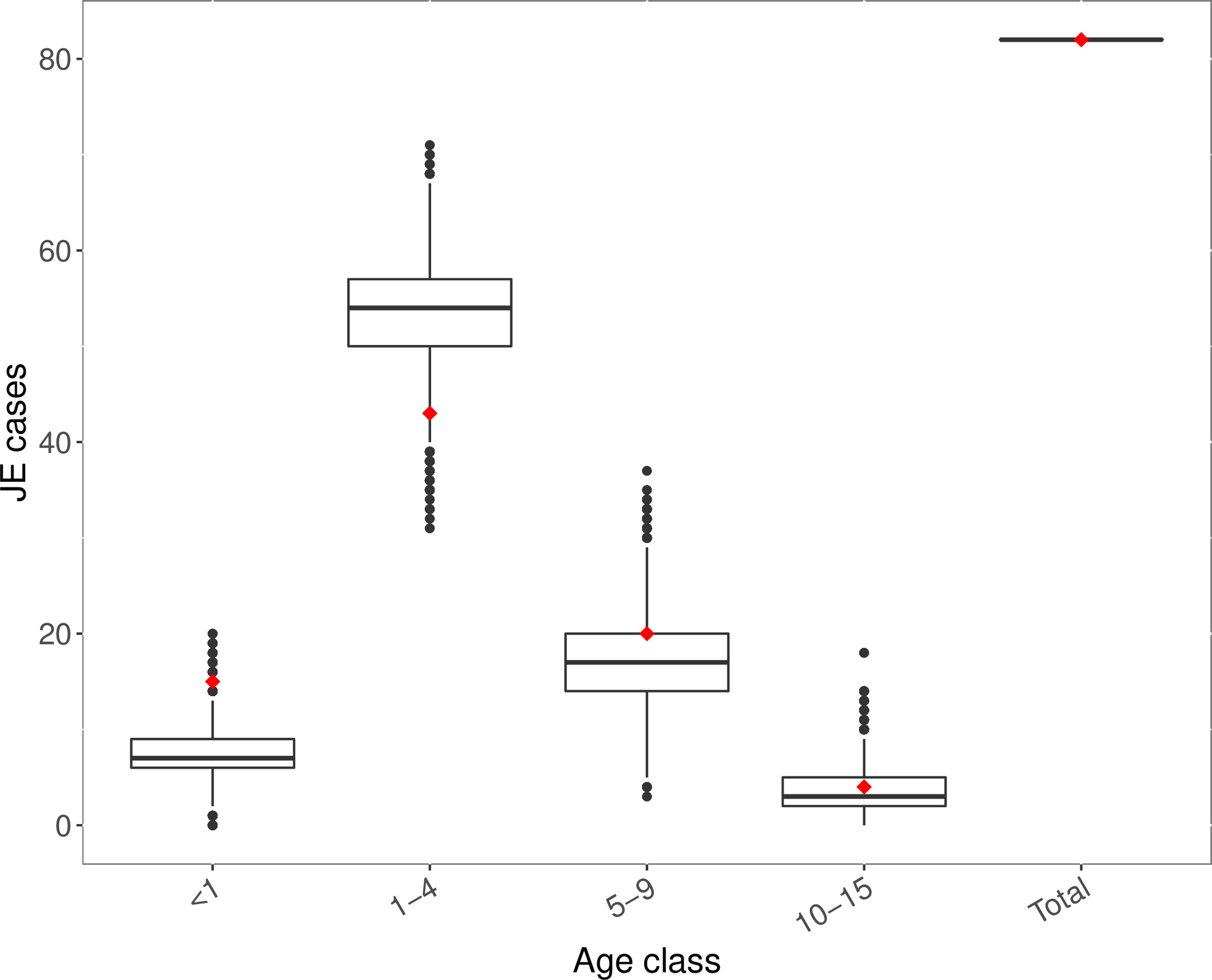

Supplement: S13 Fig — Boxplots represent predicted number of cases per age class based on draws from the joint posterior distribution of FOI and vaccination coverage (if included) estimates. Red diamonds represent the observed number of cases. (TIF) [file pntd.0009385.s017.tif]

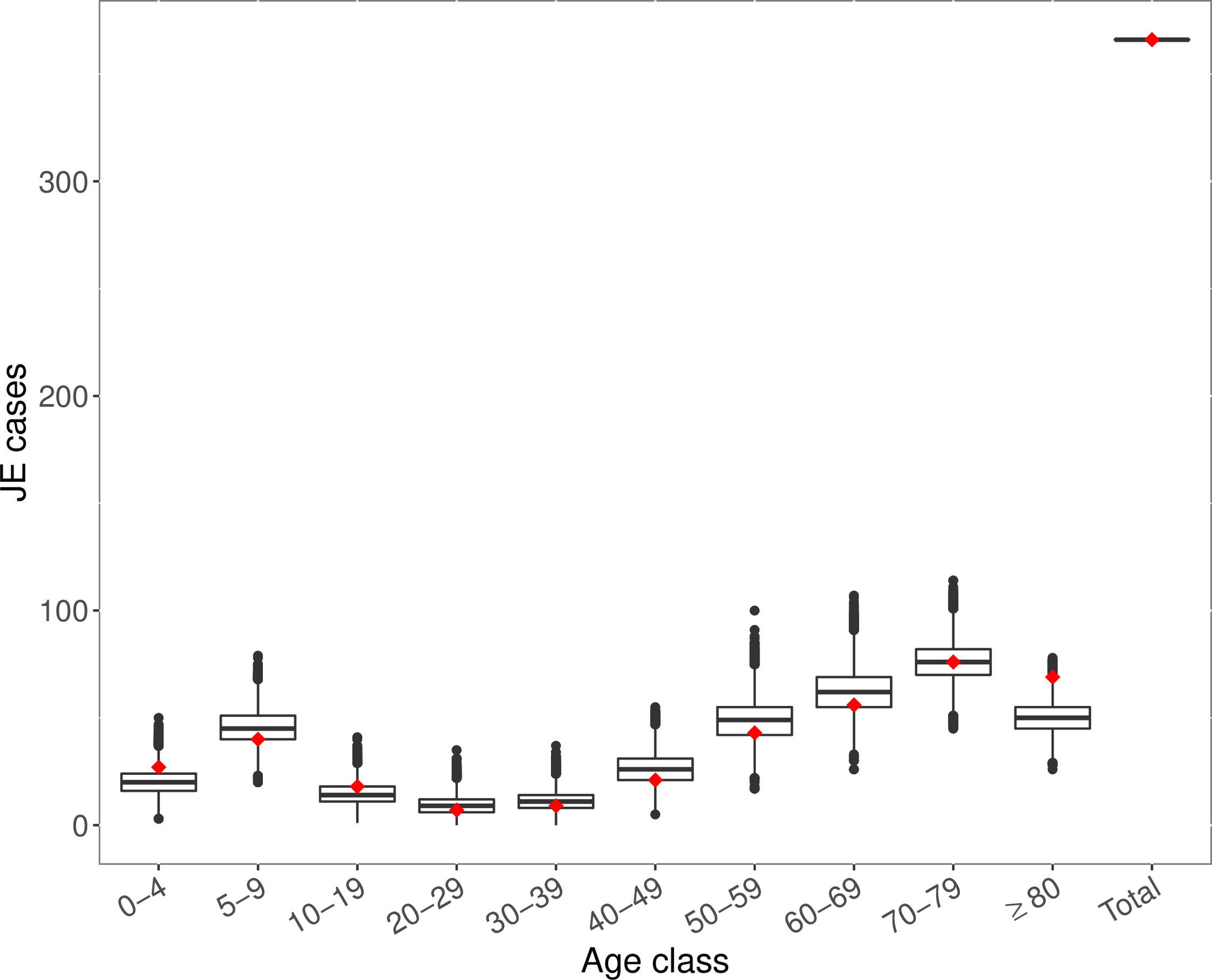

Supplement: S14 Fig — Boxplots represent predicted number of cases per age class based on draws from the joint posterior distribution of FOI and vaccination coverage (if included) estimates. Red diamonds represent the observed number of cases. (TIF) [file pntd.0009385.s018.tif]

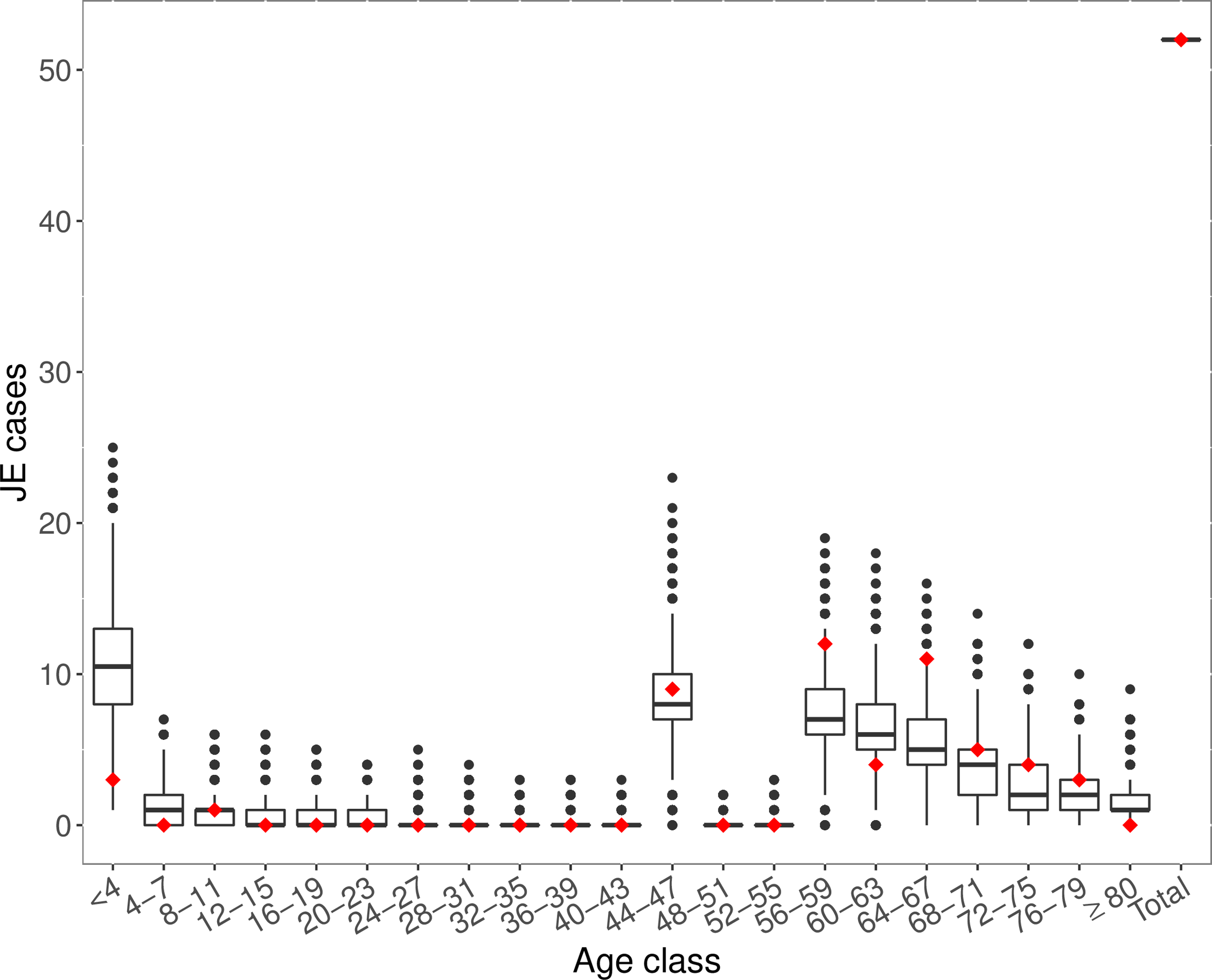

Supplement: S15 Fig — Boxplots represent predicted number of cases per age class based on draws from the joint posterior distribution of FOI and vaccination coverage (if included) estimates. Red diamonds represent the observed number of cases. (TIF) [file pntd.0009385.s019.tif]

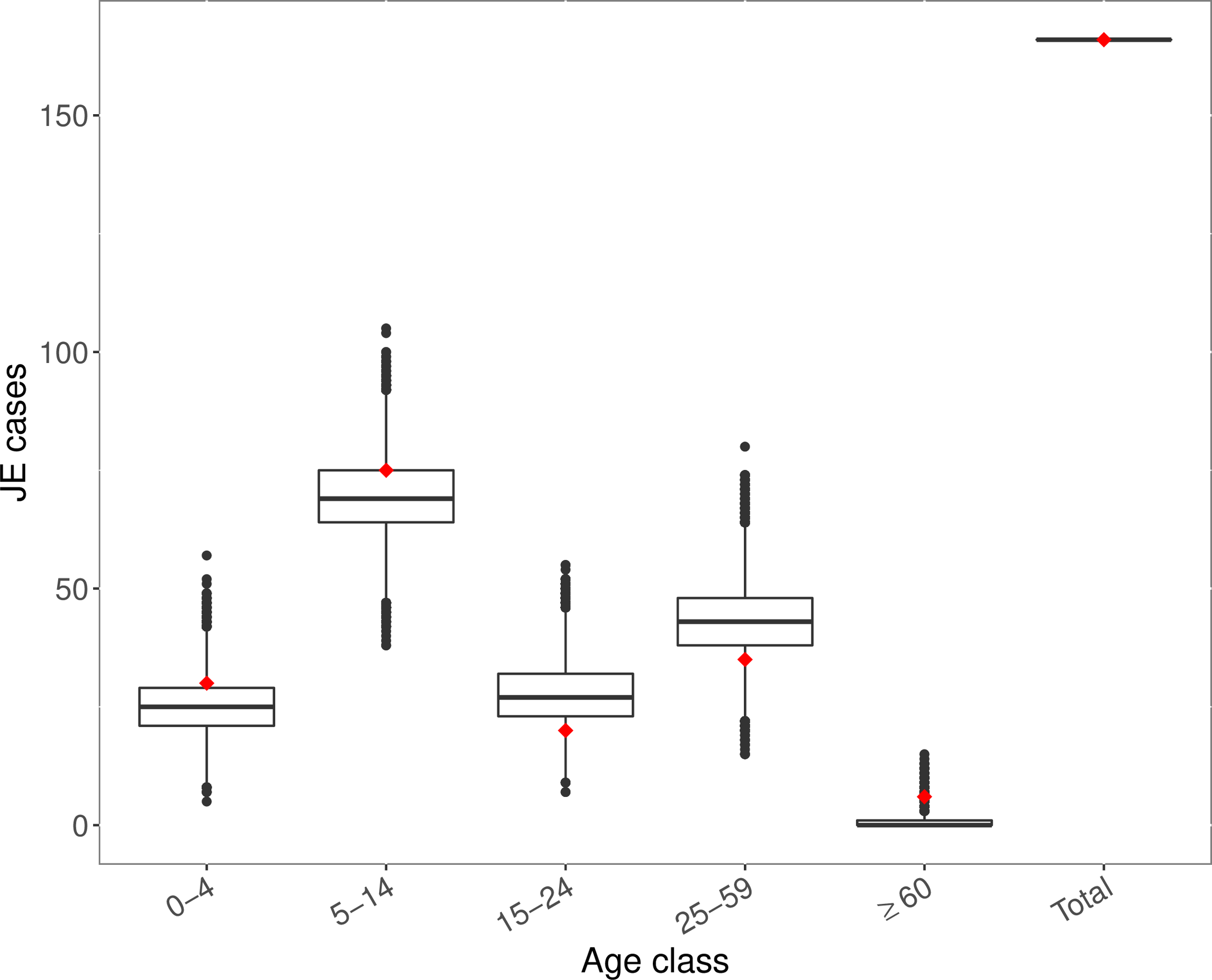

Supplement: S16 Fig — Boxplots represent predicted number of cases per age class based on draws from the joint posterior distribution of FOI and vaccination coverage (if included) estimates. Red diamonds represent the observed number of cases. (TIF) [file pntd.0009385.s020.tif]

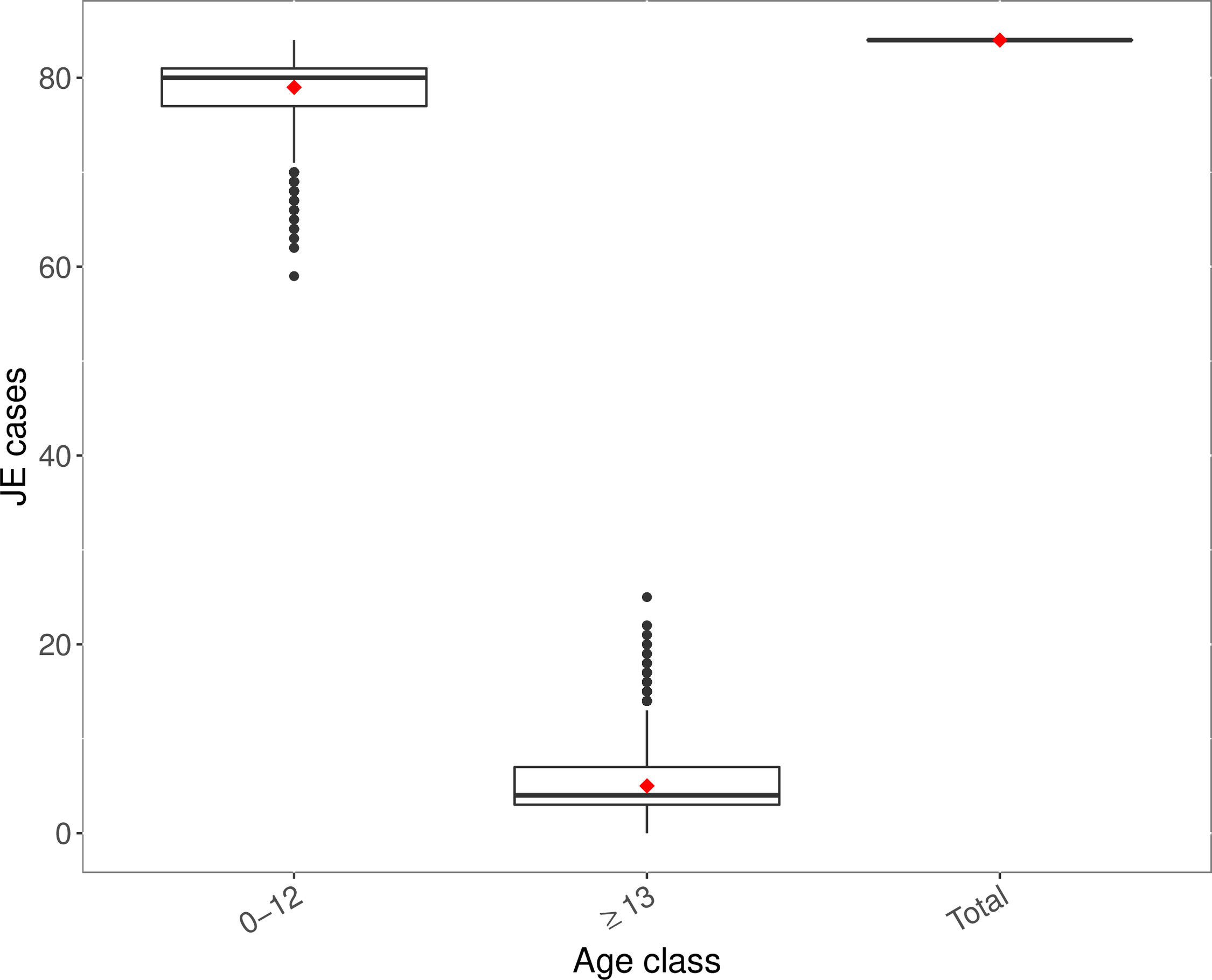

Supplement: S17 Fig — Boxplots represent predicted number of cases per age class based on draws from the joint posterior distribution of FOI and vaccination coverage (if included) estimates. Red diamonds represent the observed number of cases. (TIF) [file pntd.0009385.s021.tif]

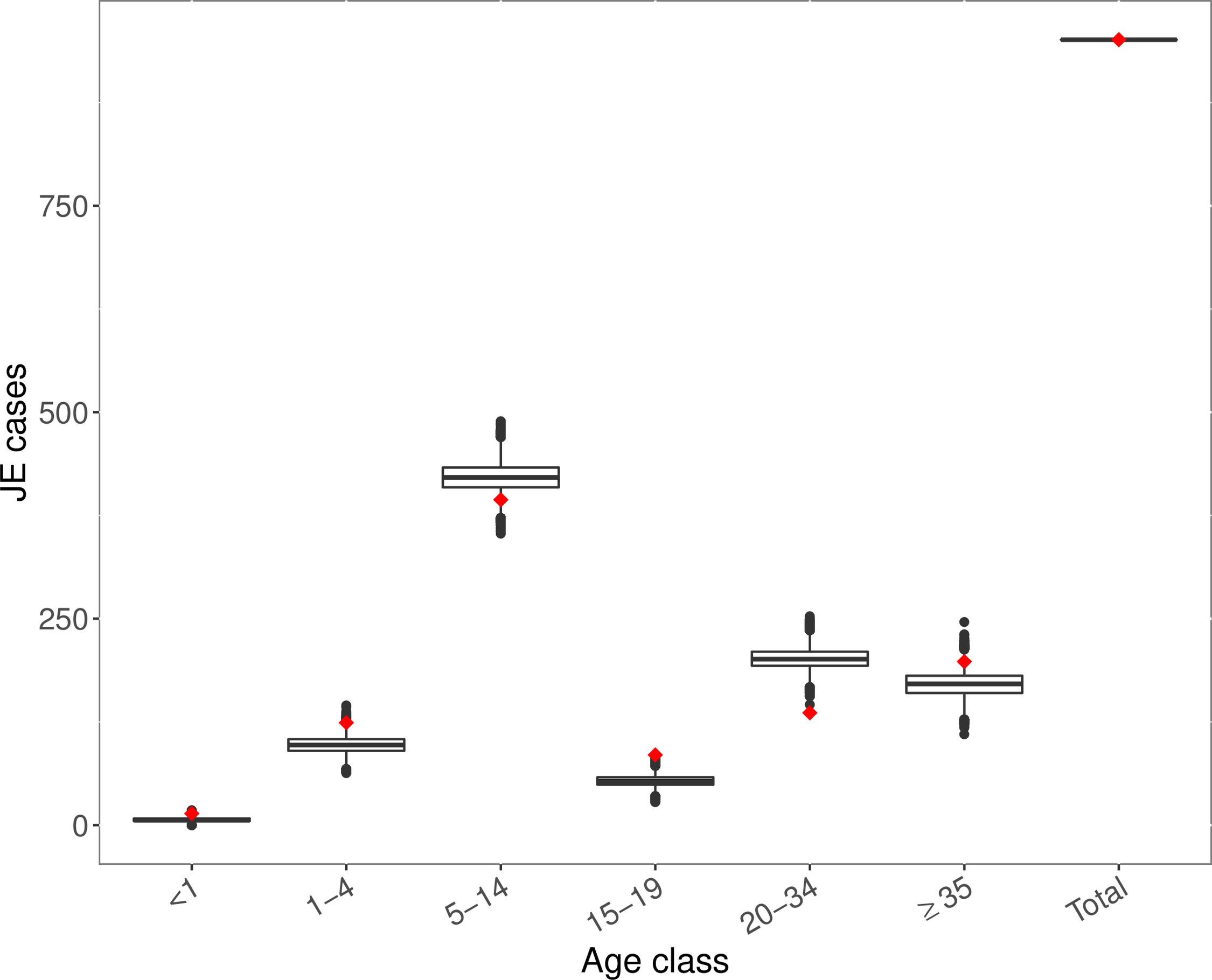

Supplement: S18 Fig — Boxplots represent predicted number of cases per age class based on draws from the joint posterior distribution of FOI and vaccination coverage (if included) estimates. Red diamonds represent the observed number of cases. (TIF) [file pntd.0009385.s022.tif]

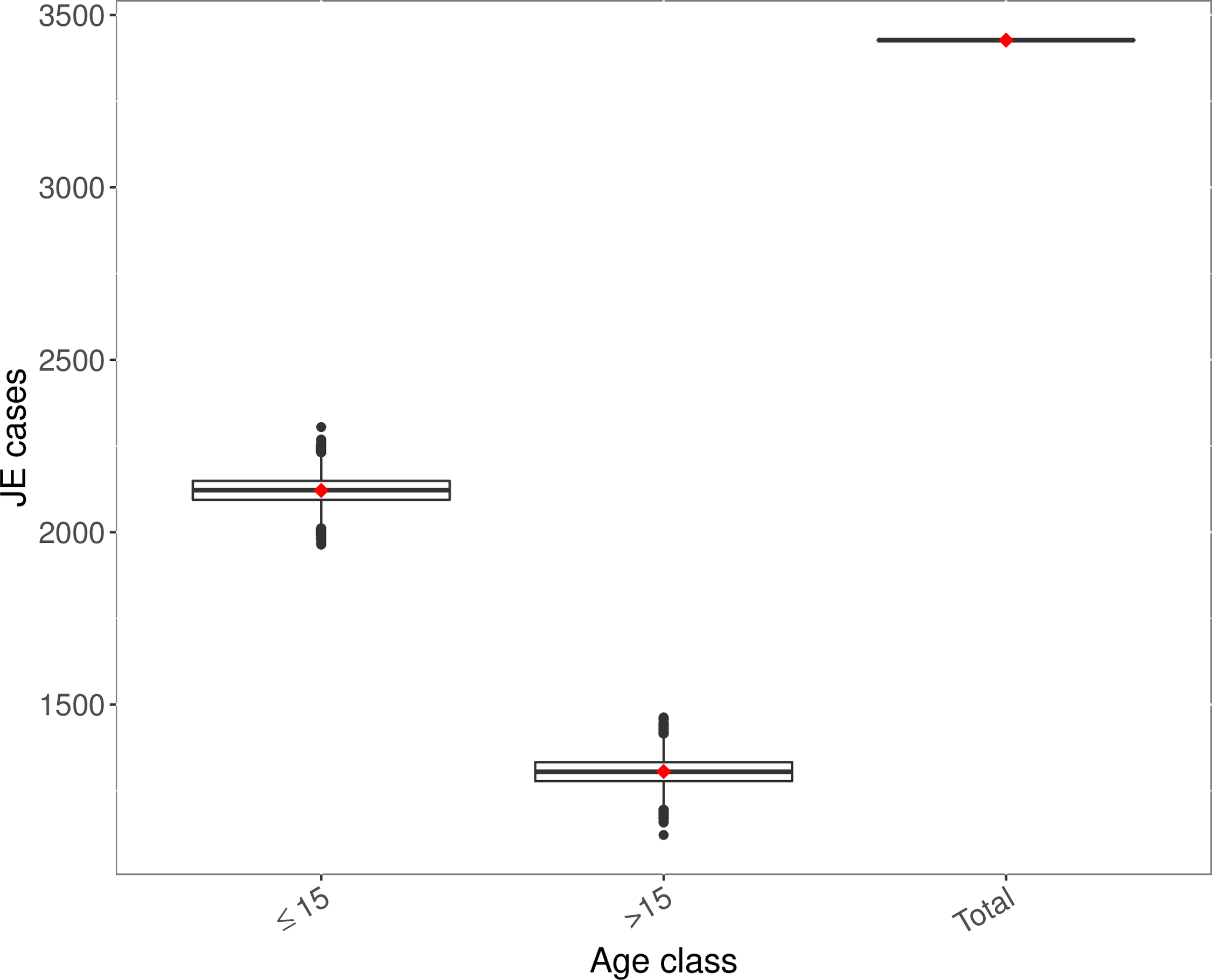

Supplement: S19 Fig — Boxplots represent predicted number of cases per age class based on draws from the joint posterior distribution of FOI and vaccination coverage (if included) estimates. Red diamonds represent the observed number of cases. (TIF) [file pntd.0009385.s023.tif]

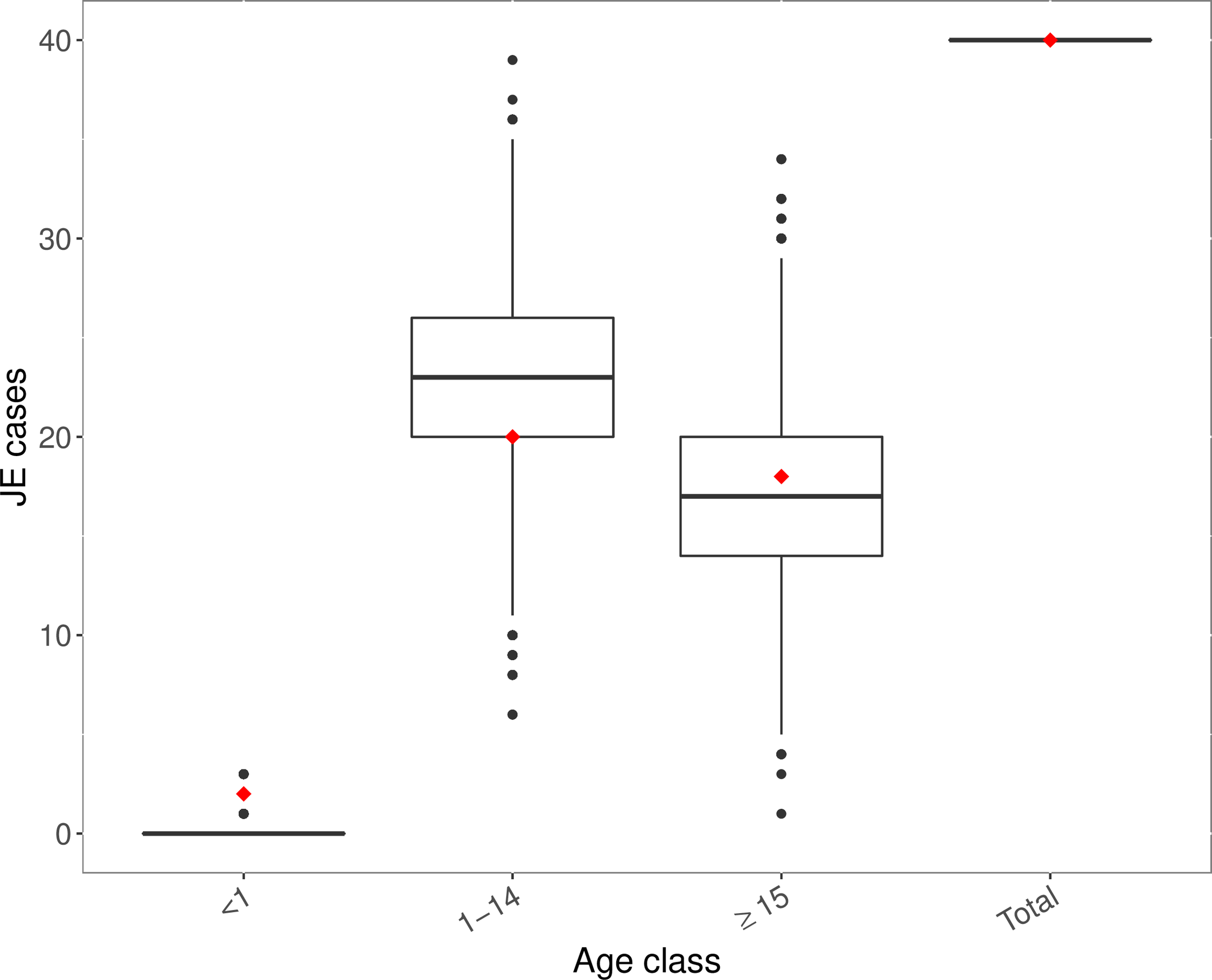

Supplement: S20 Fig — Boxplots represent predicted number of cases per age class based on draws from the joint posterior distribution of FOI and vaccination coverage (if included) estimates. Red diamonds represent the observed number of cases. (TIF) [file pntd.0009385.s024.tif]

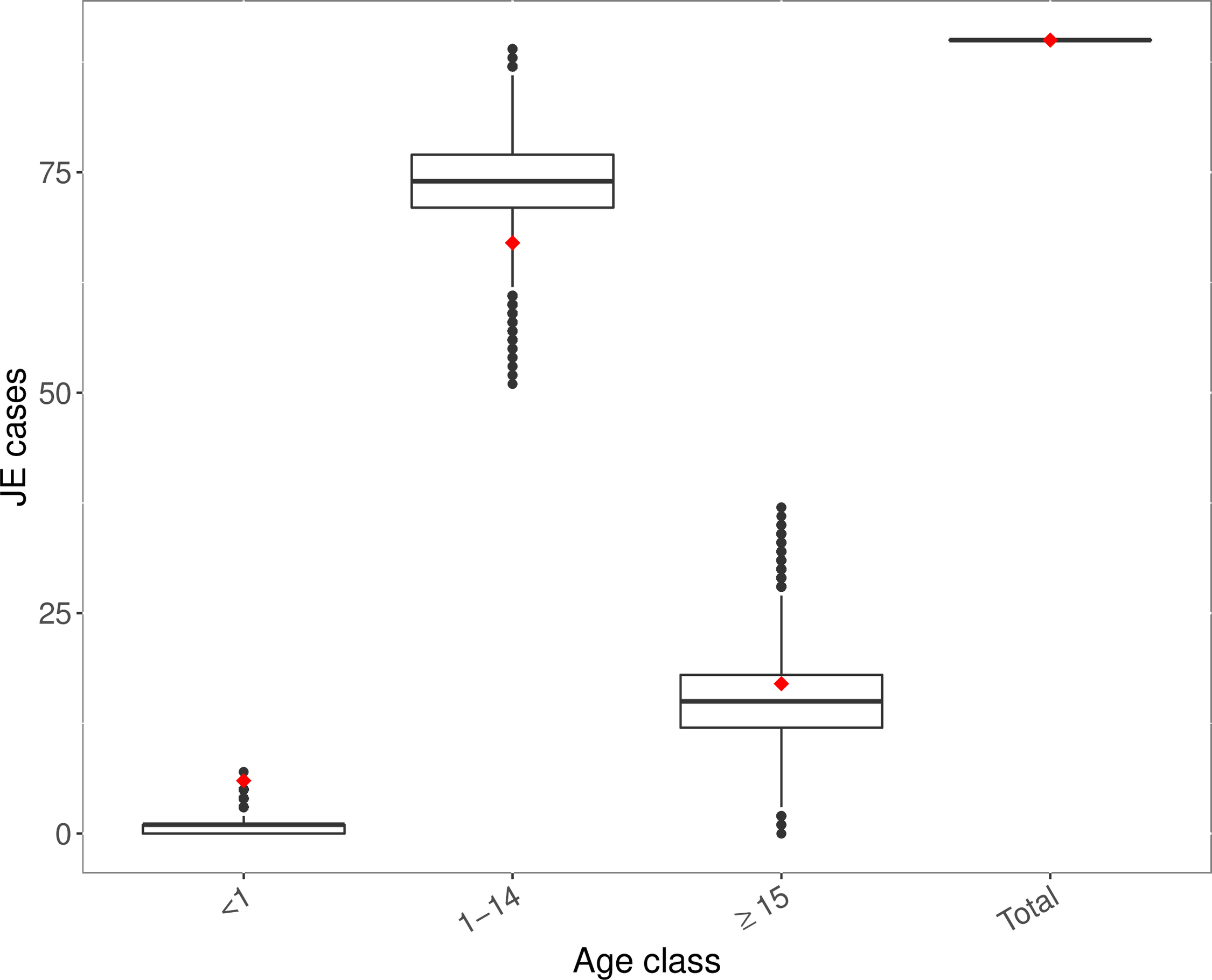

Supplement: S21 Fig — Boxplots represent predicted number of cases per age class based on draws from the joint posterior distribution of FOI and vaccination coverage (if included) estimates. Red diamonds represent the observed number of cases. (TIF) [file pntd.0009385.s025.tif]

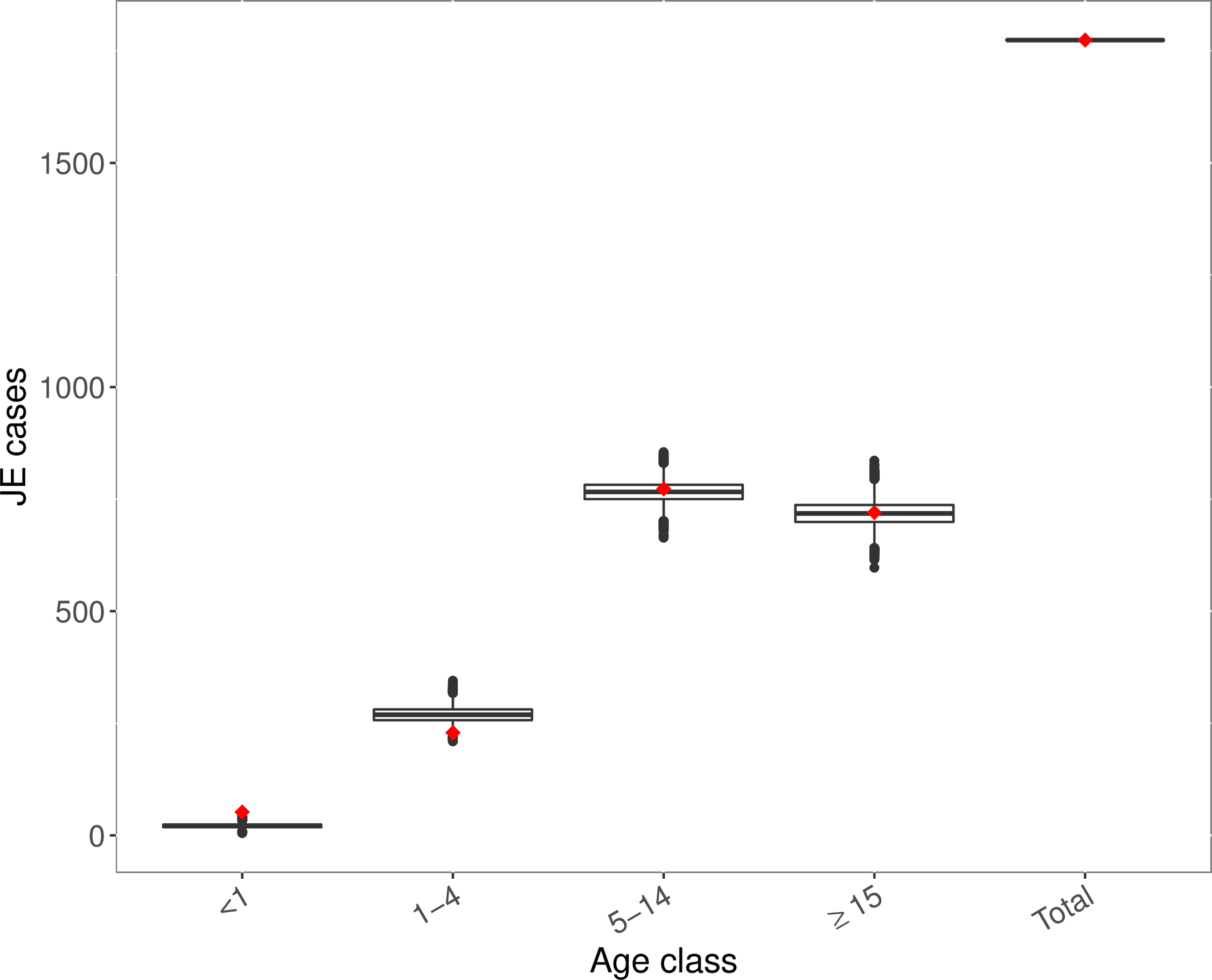

Supplement: S22 Fig — Boxplots represent predicted number of cases per age class based on draws from the joint posterior distribution of FOI and vaccination coverage (if included) estimates. Red diamonds represent the observed number of cases. (TIF) [file pntd.0009385.s026.tif]

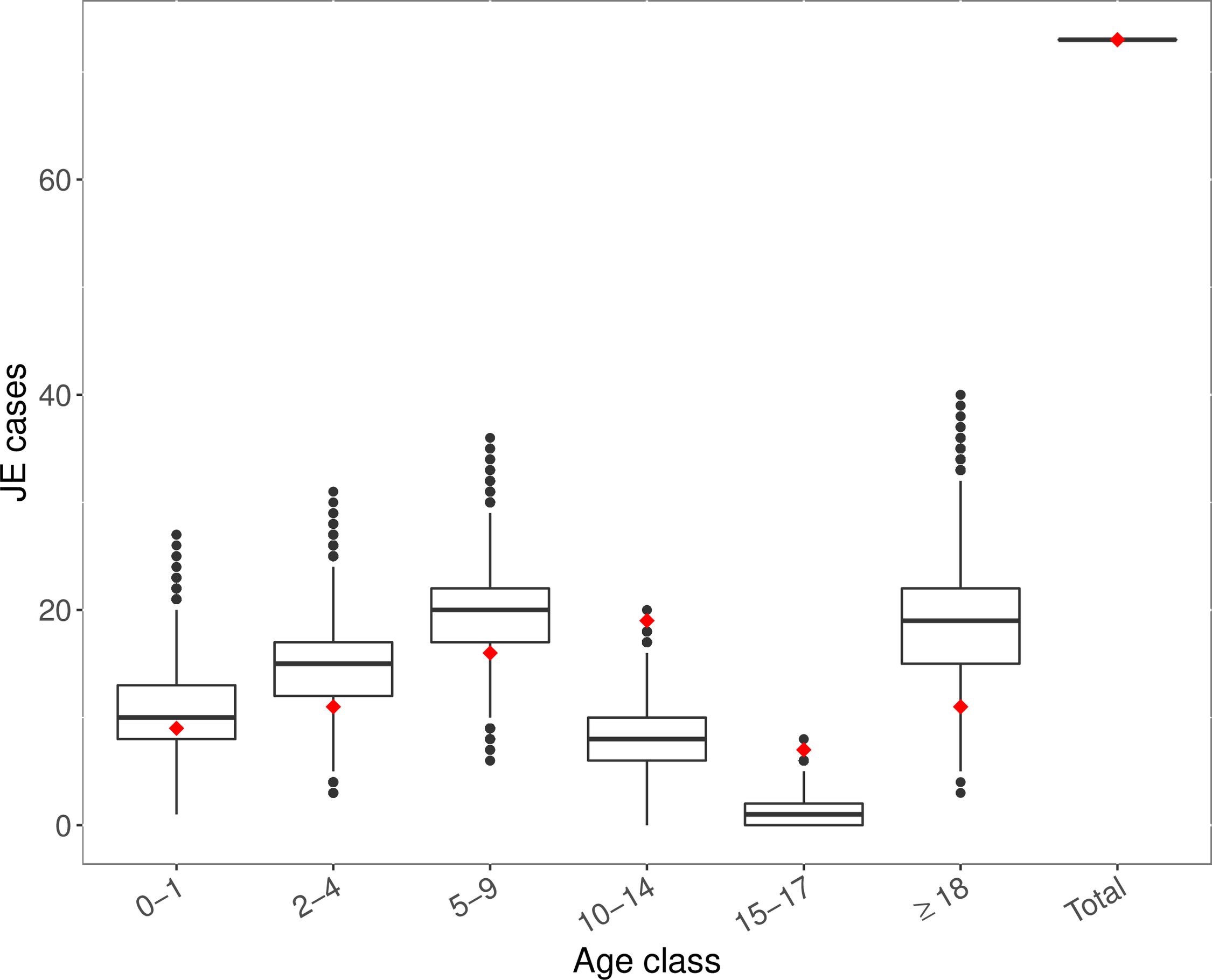

Supplement: S23 Fig — Boxplots represent predicted number of cases per age class based on draws from the joint posterior distribution of FOI and vaccination coverage (if included) estimates. Red diamonds represent the observed number of cases. (TIF) [file pntd.0009385.s027.tif]

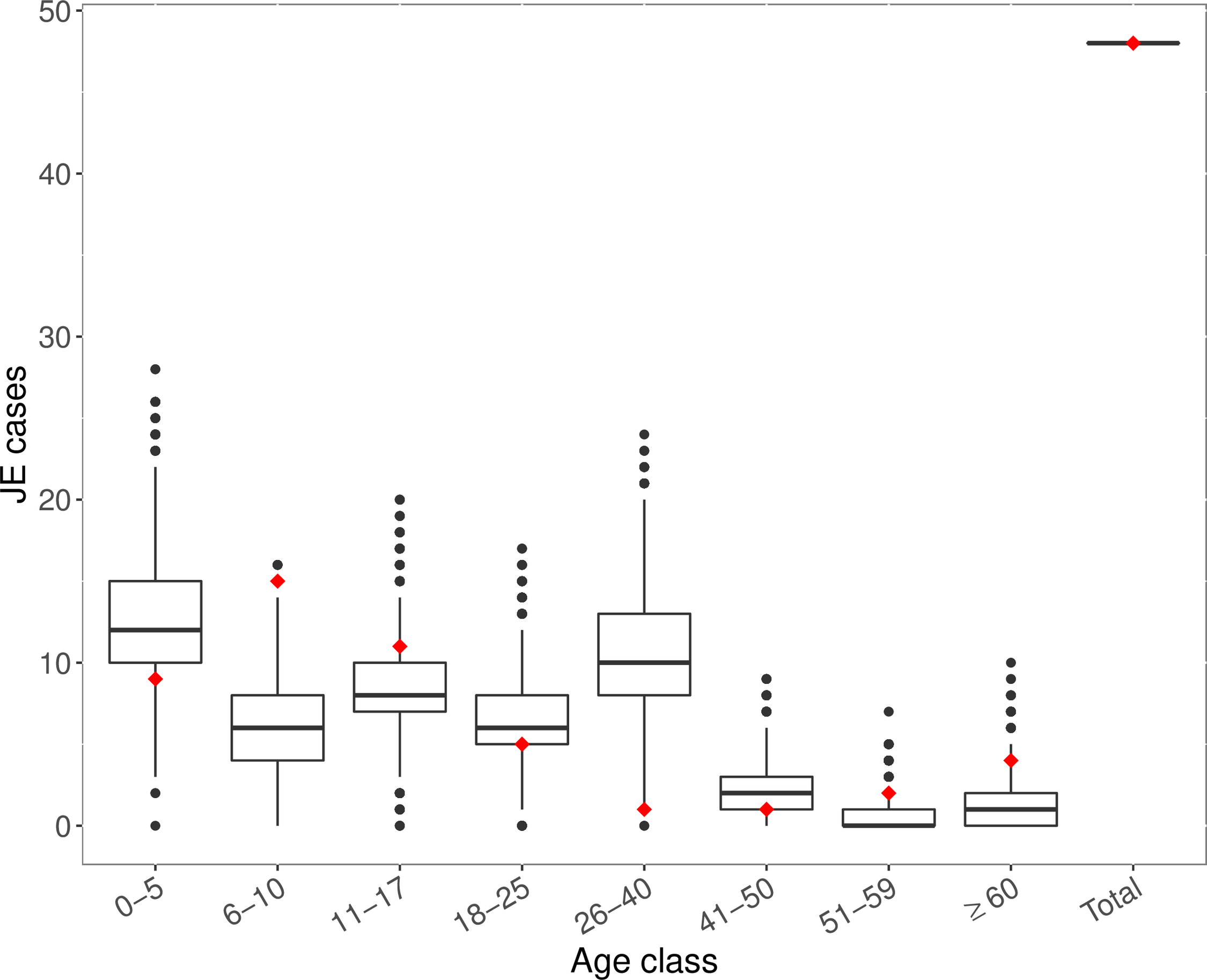

Supplement: S24 Fig — Boxplots represent predicted number of cases per age class based on draws from the joint posterior distribution of FOI and vaccination coverage (if included) estimates. Red diamonds represent the observed number of cases. (TIF) [file pntd.0009385.s028.tif]

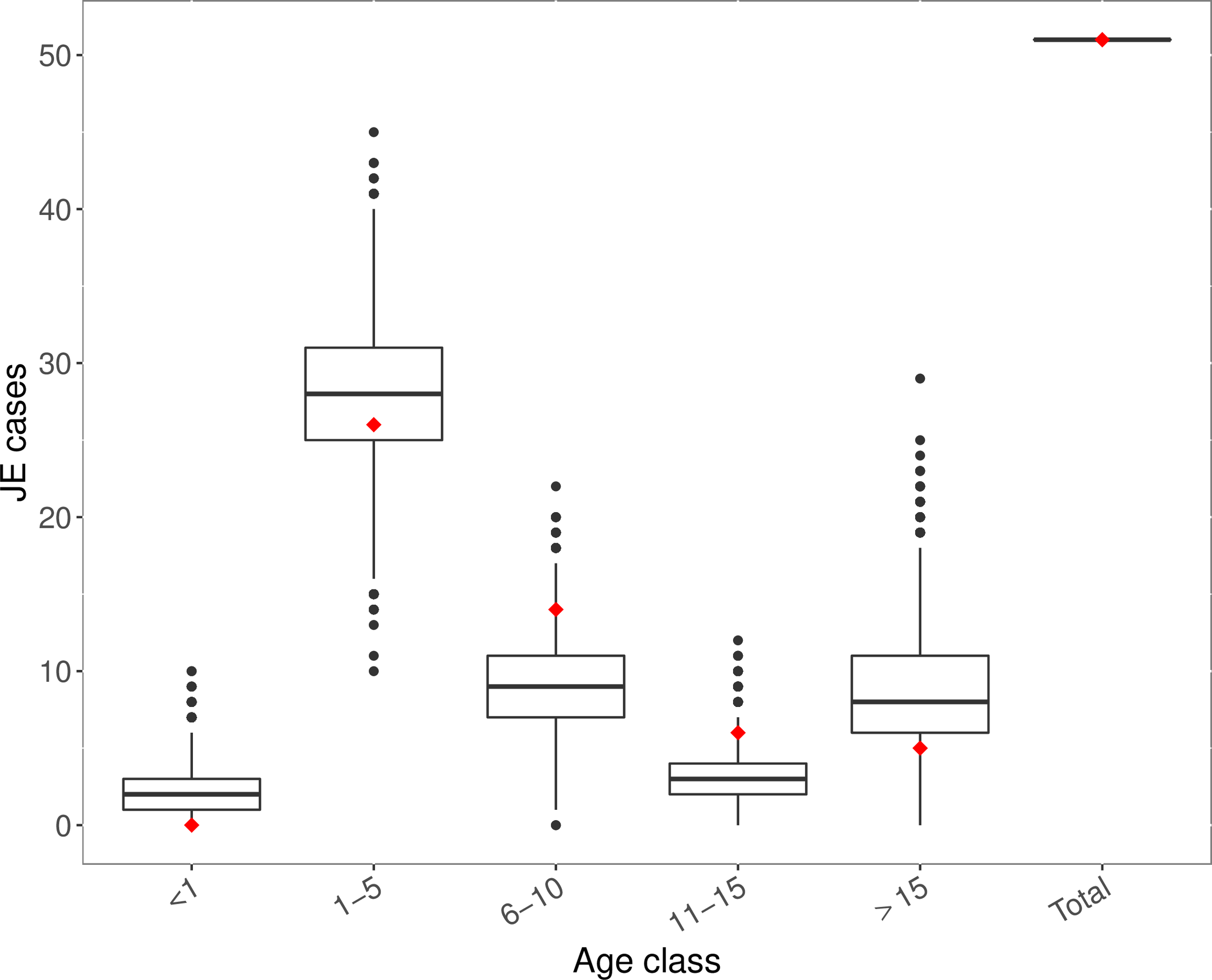

Supplement: S25 Fig — Boxplots represent predicted number of cases per age class based on draws from the joint posterior distribution of FOI and vaccination coverage (if included) estimates. Red diamonds represent the observed number of cases. (TIF) [file pntd.0009385.s029.tif]

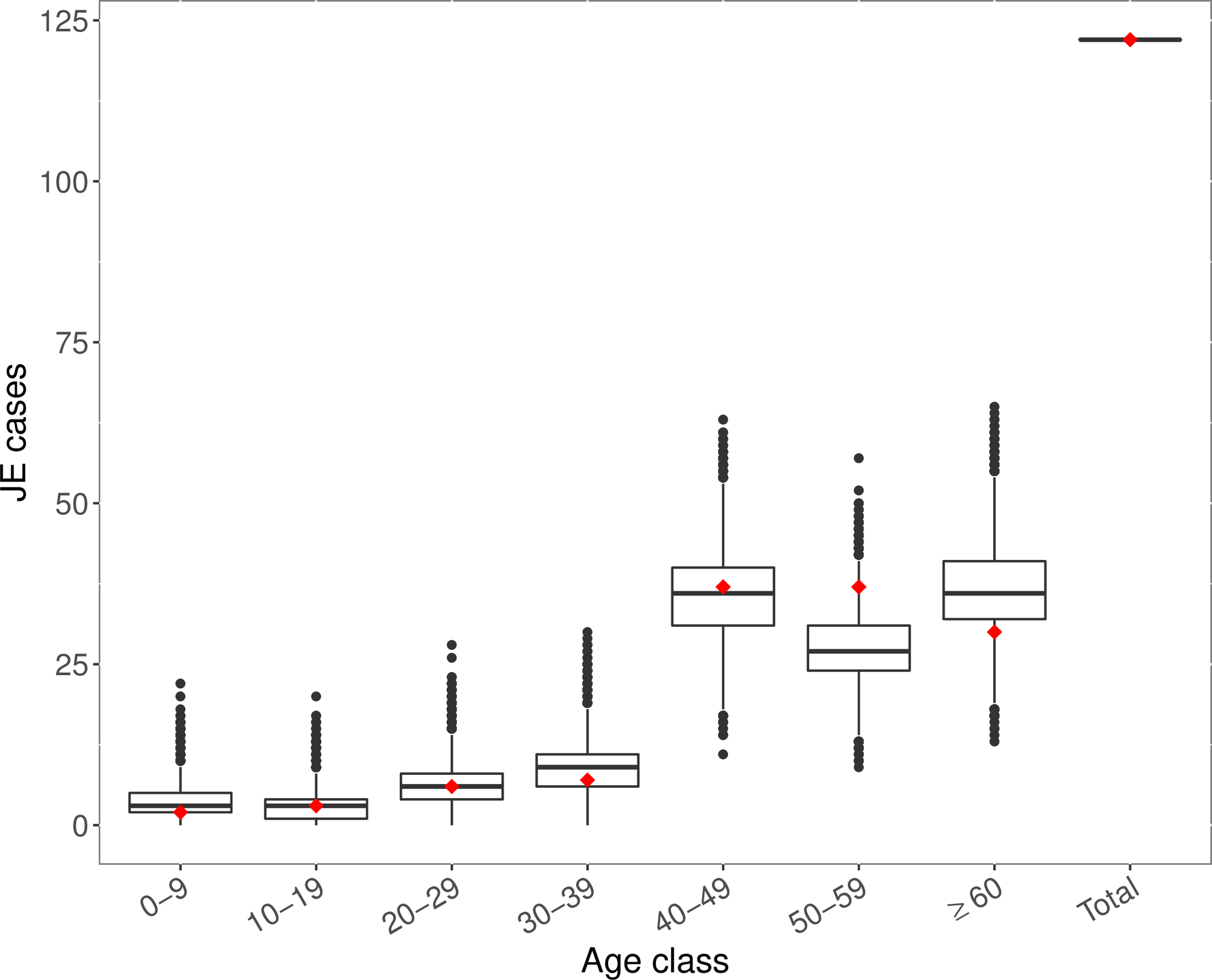

Supplement: S26 Fig — Boxplots represent predicted number of cases per age class based on draws from the joint posterior distribution of FOI and vaccination coverage (if included) estimates. Red diamonds represent the observed number of cases. (TIF) [file pntd.0009385.s030.tif]

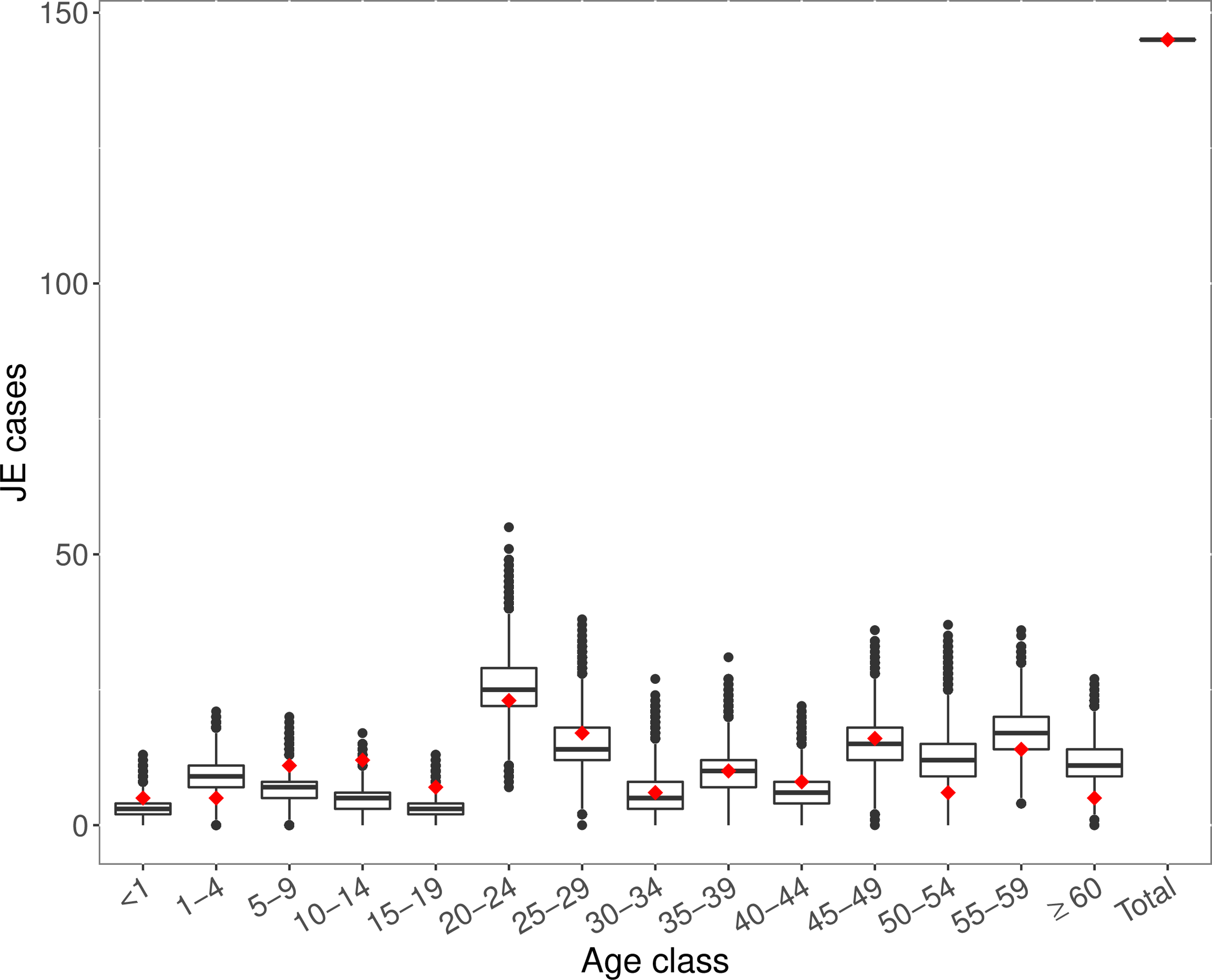

Supplement: S27 Fig — Boxplots represent predicted number of cases per age class based on draws from the joint posterior distribution of FOI and vaccination coverage (if included) estimates. Red diamonds represent the observed number of cases. (TIF) [file pntd.0009385.s031.tif]

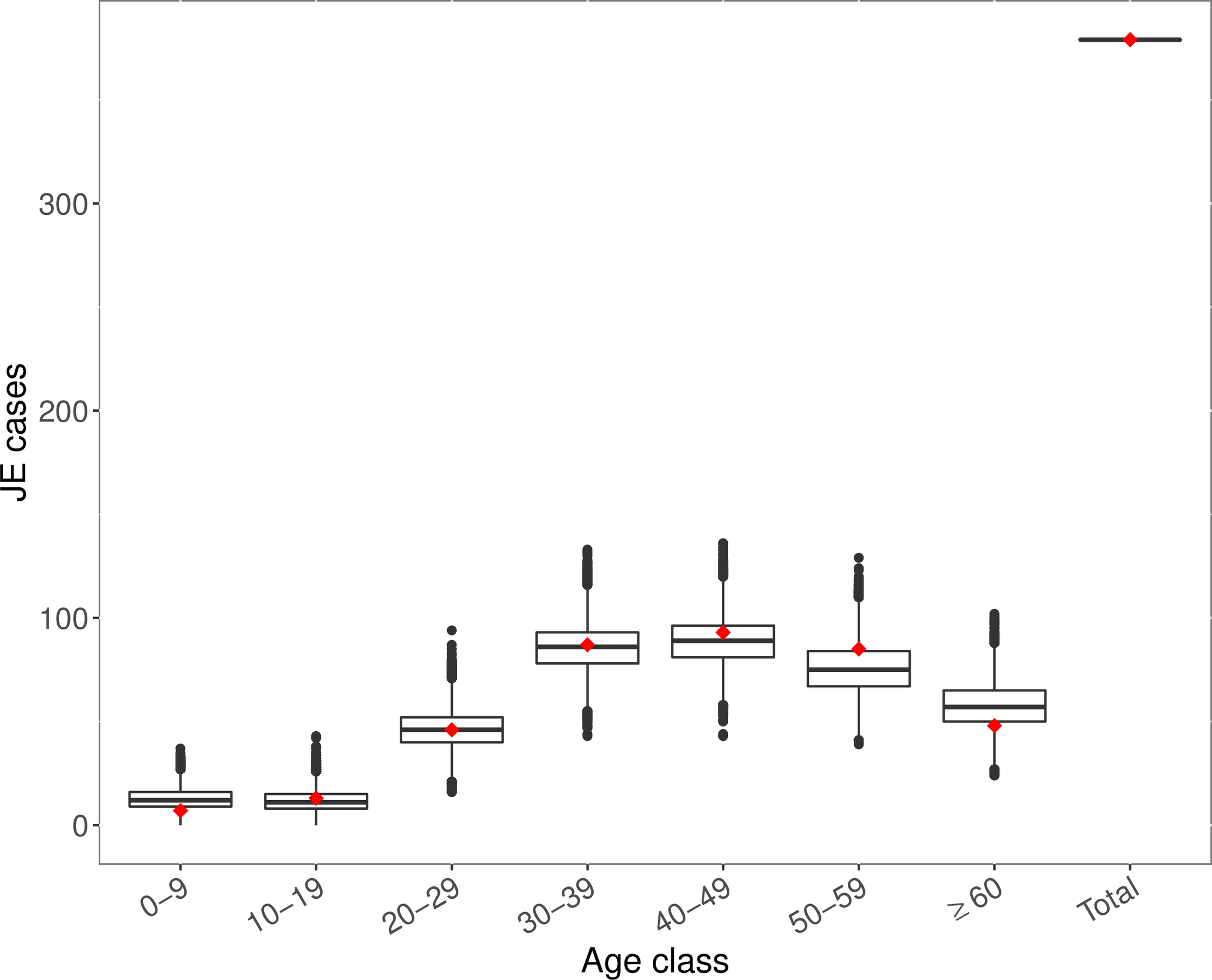

Supplement: S28 Fig — Boxplots represent predicted number of cases per age class based on draws from the joint posterior distribution of FOI and vaccination coverage (if included) estimates. Red diamonds represent the observed number of cases. (TIF) [file pntd.0009385.s032.tif]

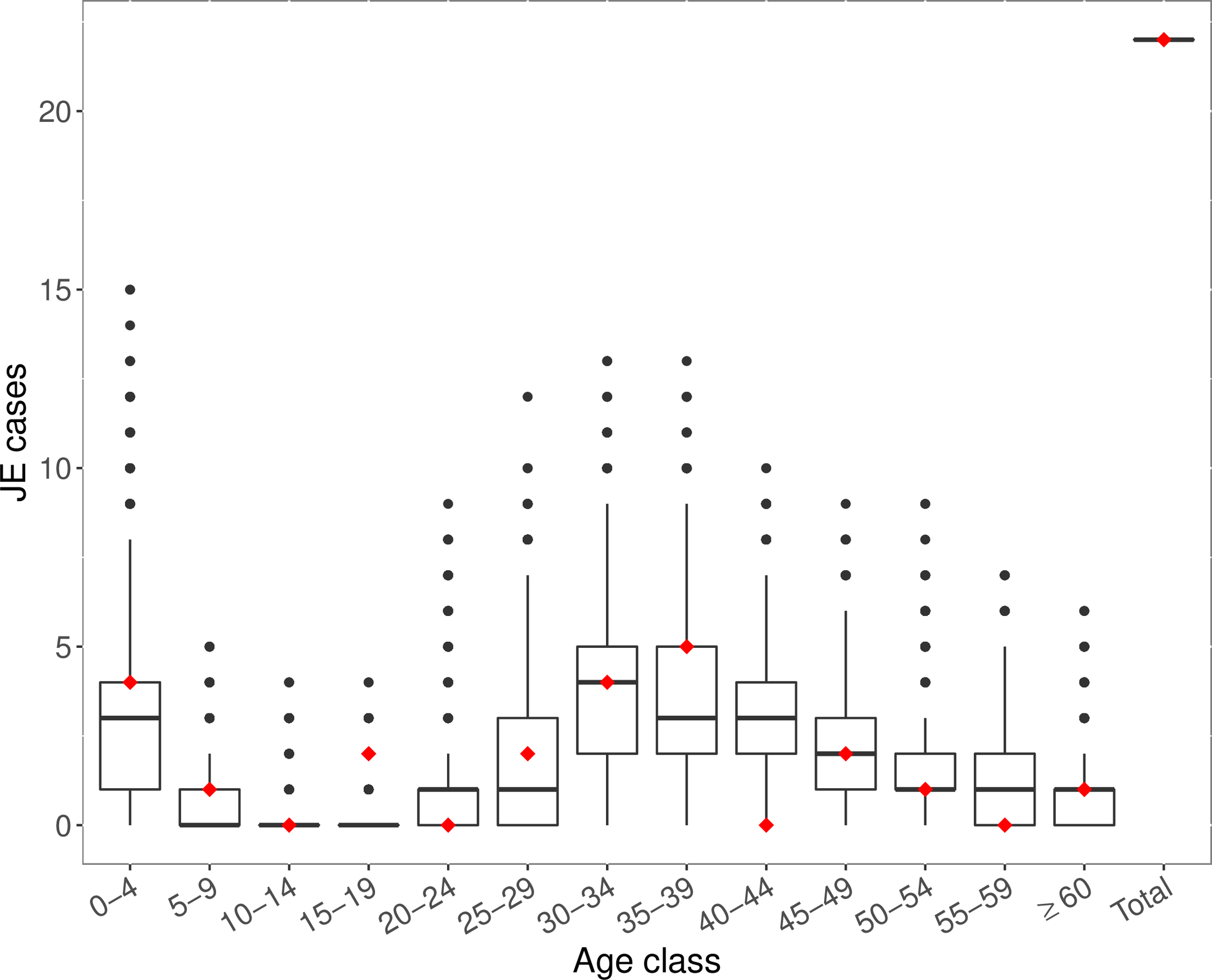

Supplement: S29 Fig — Boxplots represent predicted number of cases per age class based on draws from the joint posterior distribution of FOI and vaccination coverage (if included) estimates. Red diamonds represent the observed number of cases. (TIF) [file pntd.0009385.s033.tif]

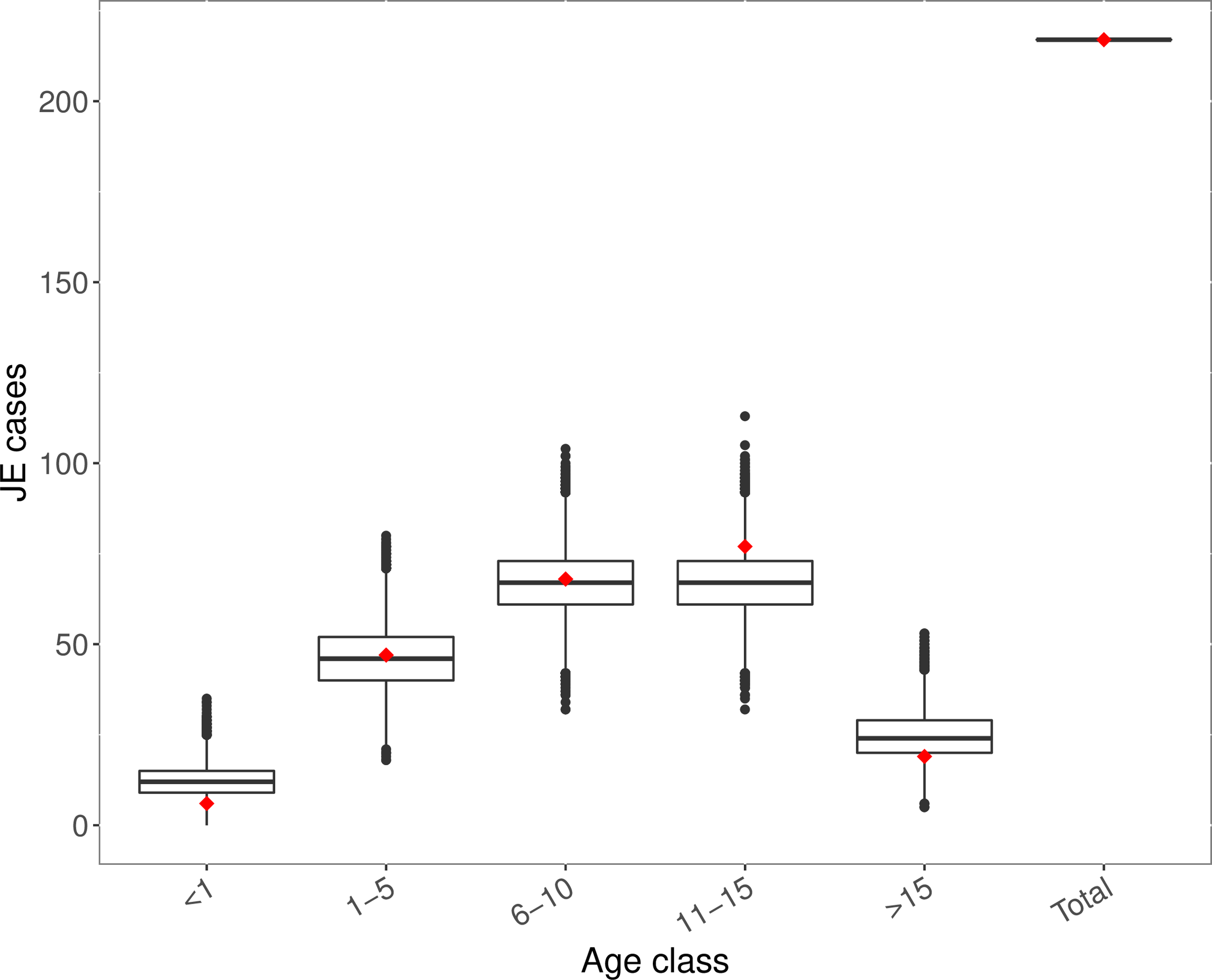

Supplement: S30 Fig — Boxplots represent predicted number of cases per age class based on draws from the joint posterior distribution of FOI and vaccination coverage (if included) estimates. Red diamonds represent the observed number of cases. (TIF) [file pntd.0009385.s034.tif]

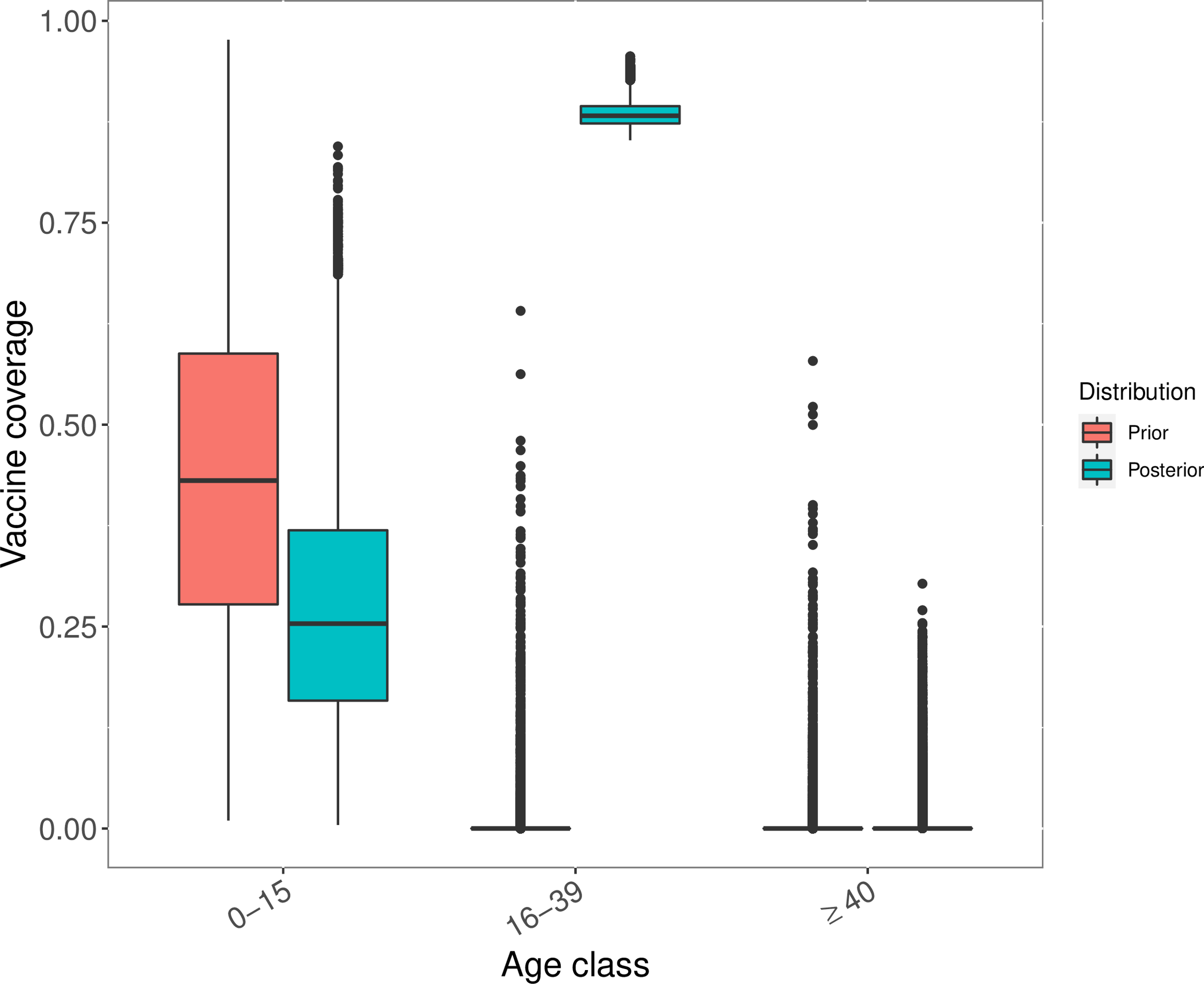

Supplement: S31 Fig — Vaccination coverage data used to generate prior distributions was taken from reference(s) listed in S1 Table. (TIF) [file pntd.0009385.s035.tif]

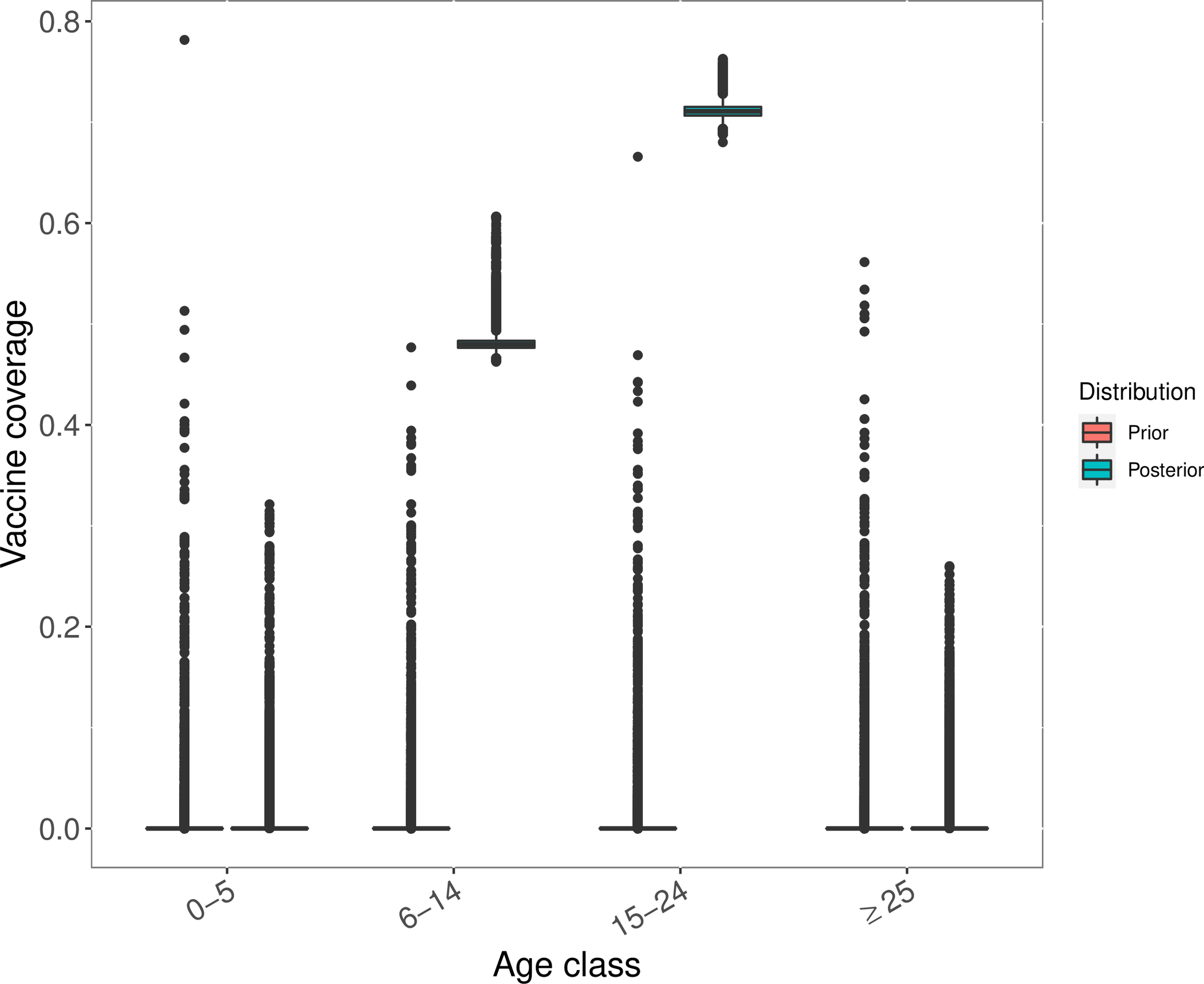

Supplement: S32 Fig — Vaccination coverage data used to generate prior distributions was taken from reference(s) listed in S1 Table. (TIF) [file pntd.0009385.s036.tif]

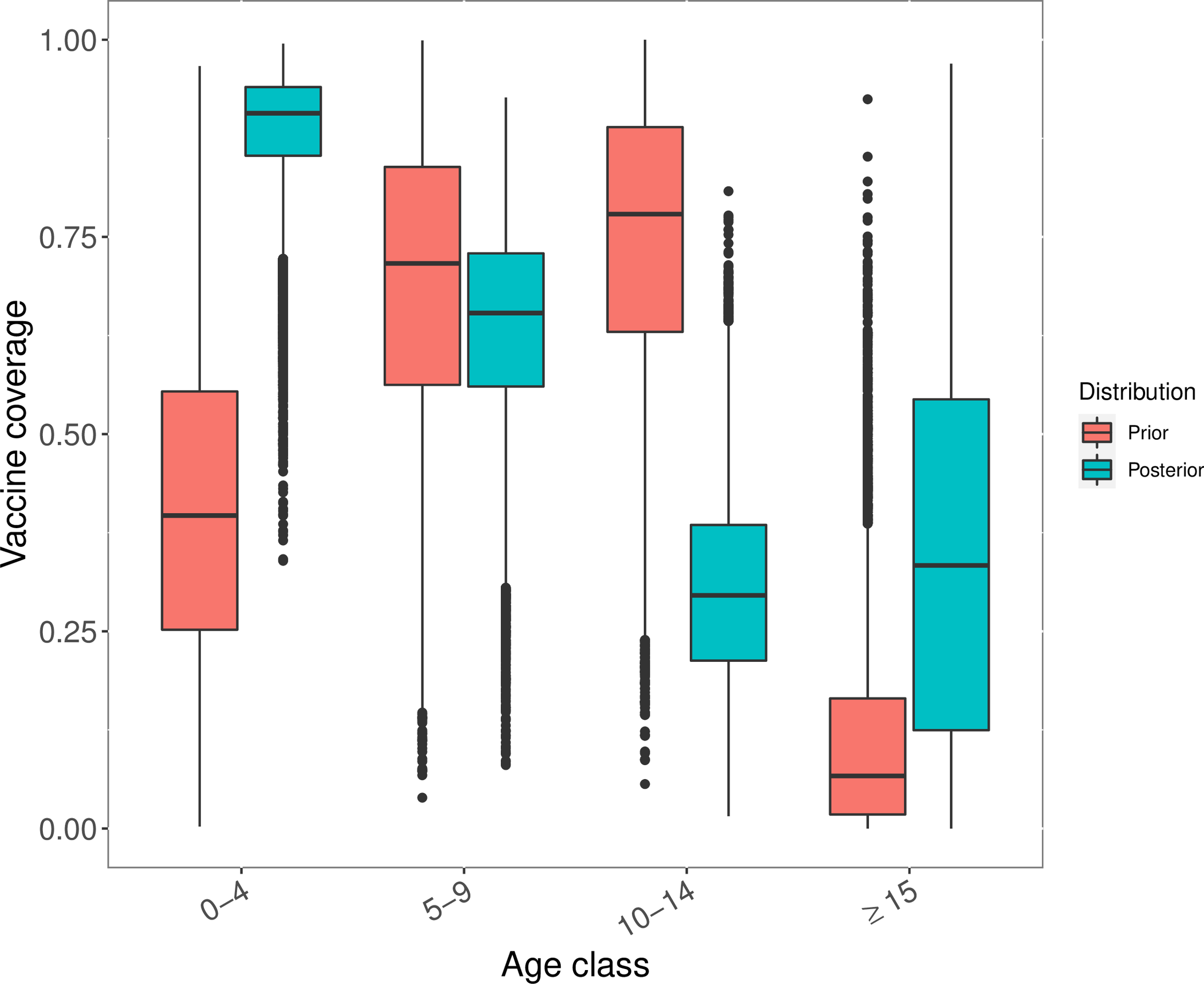

Supplement: S33 Fig — Vaccination coverage data used to generate prior distributions was taken from reference(s) listed in S1 Table. (TIF) [file pntd.0009385.s037.tif]

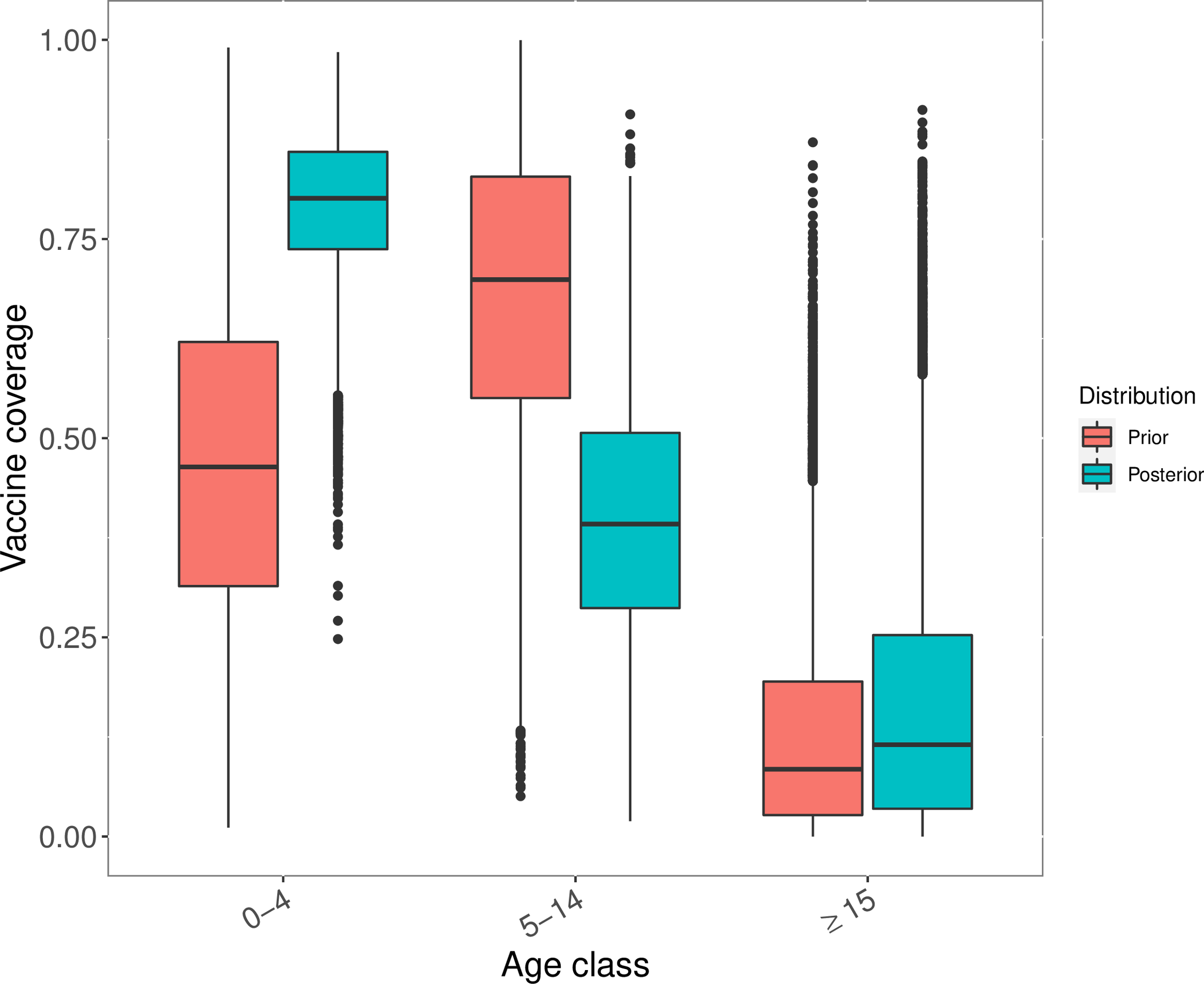

Supplement: S34 Fig — Vaccination coverage data used to generate prior distributions was taken from reference(s) listed in S1 Table. (TIF) [file pntd.0009385.s038.tif]

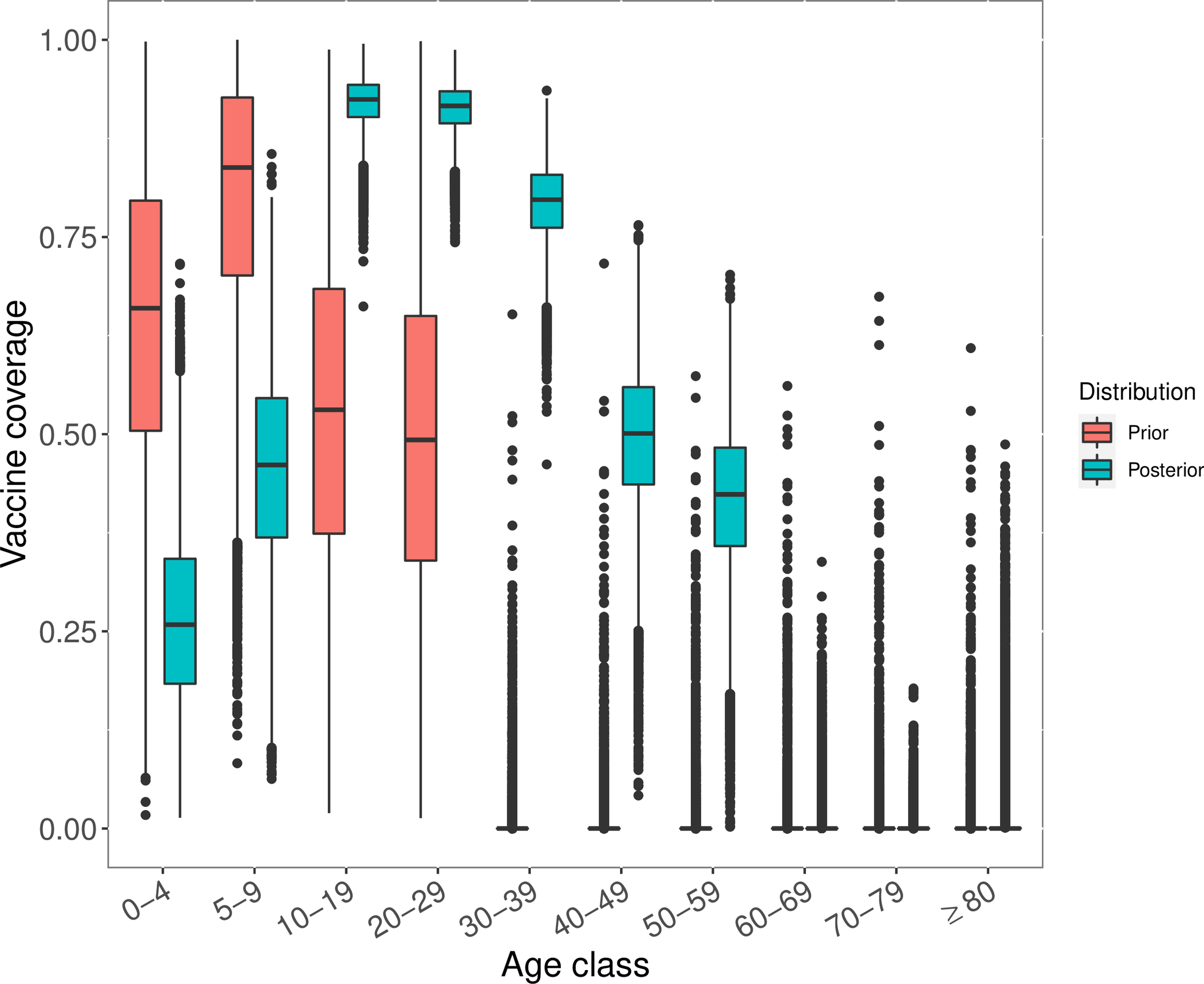

Supplement: S35 Fig — Vaccination coverage data used to generate prior distributions was taken from reference(s) listed in S1 Table. (TIF) [file pntd.0009385.s039.tif]

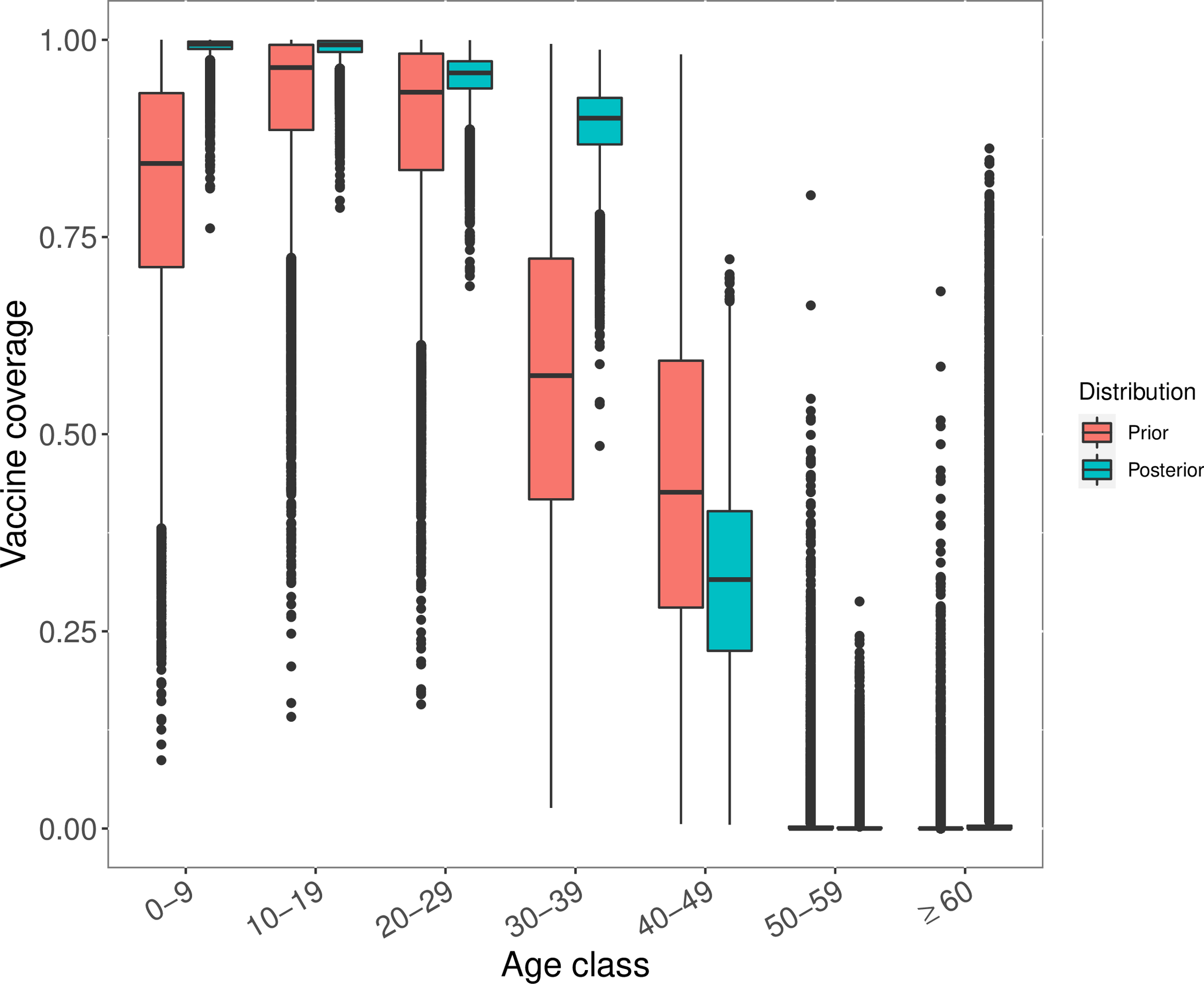

Supplement: S36 Fig — Vaccination coverage data used to generate prior distributions was taken from reference(s) listed in S1 Table. (TIF) [file pntd.0009385.s040.tif]

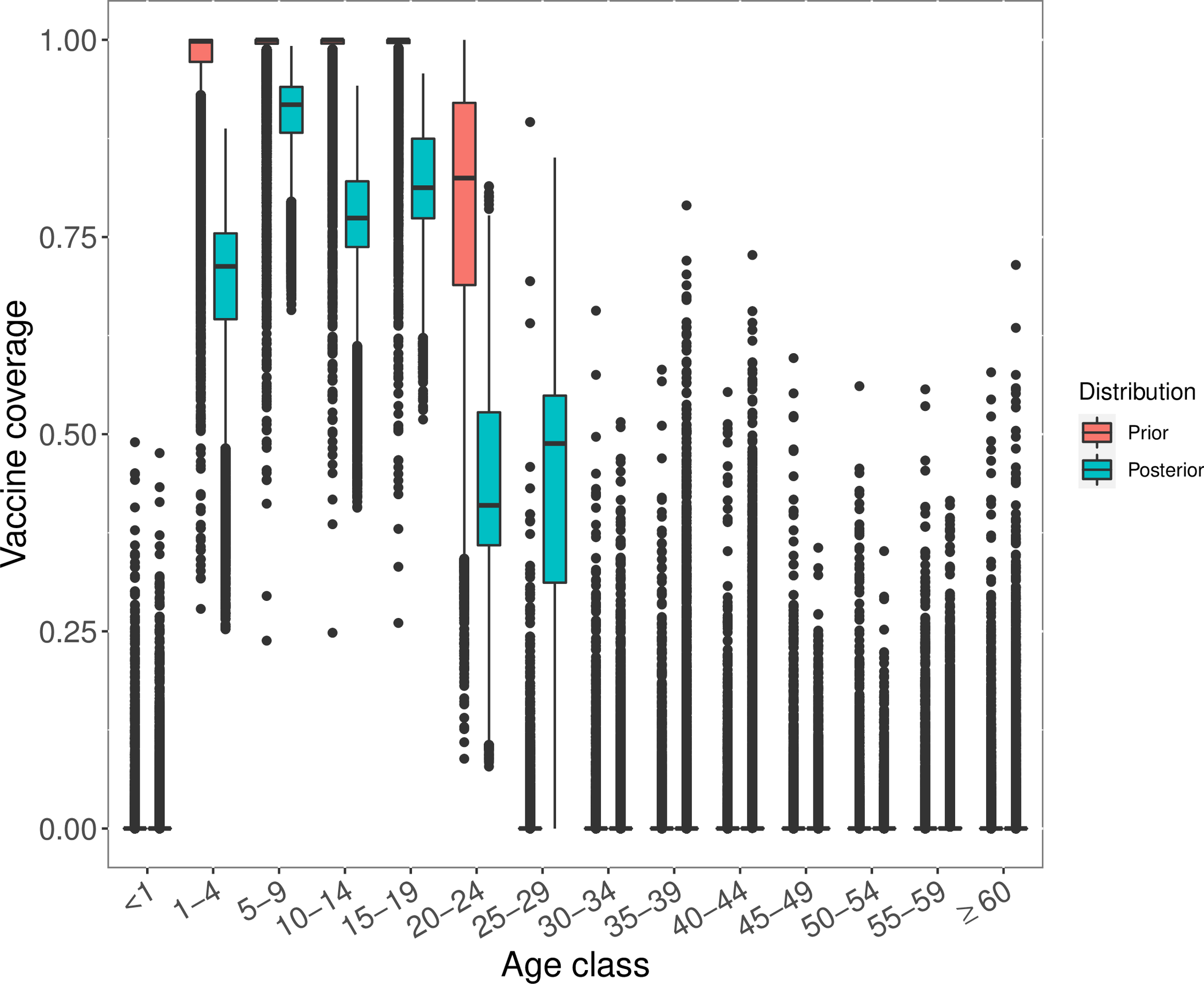

Supplement: S37 Fig — Vaccination coverage data used to generate prior distributions was taken from reference(s) listed in S1 Table. (TIF) [file pntd.0009385.s041.tif]

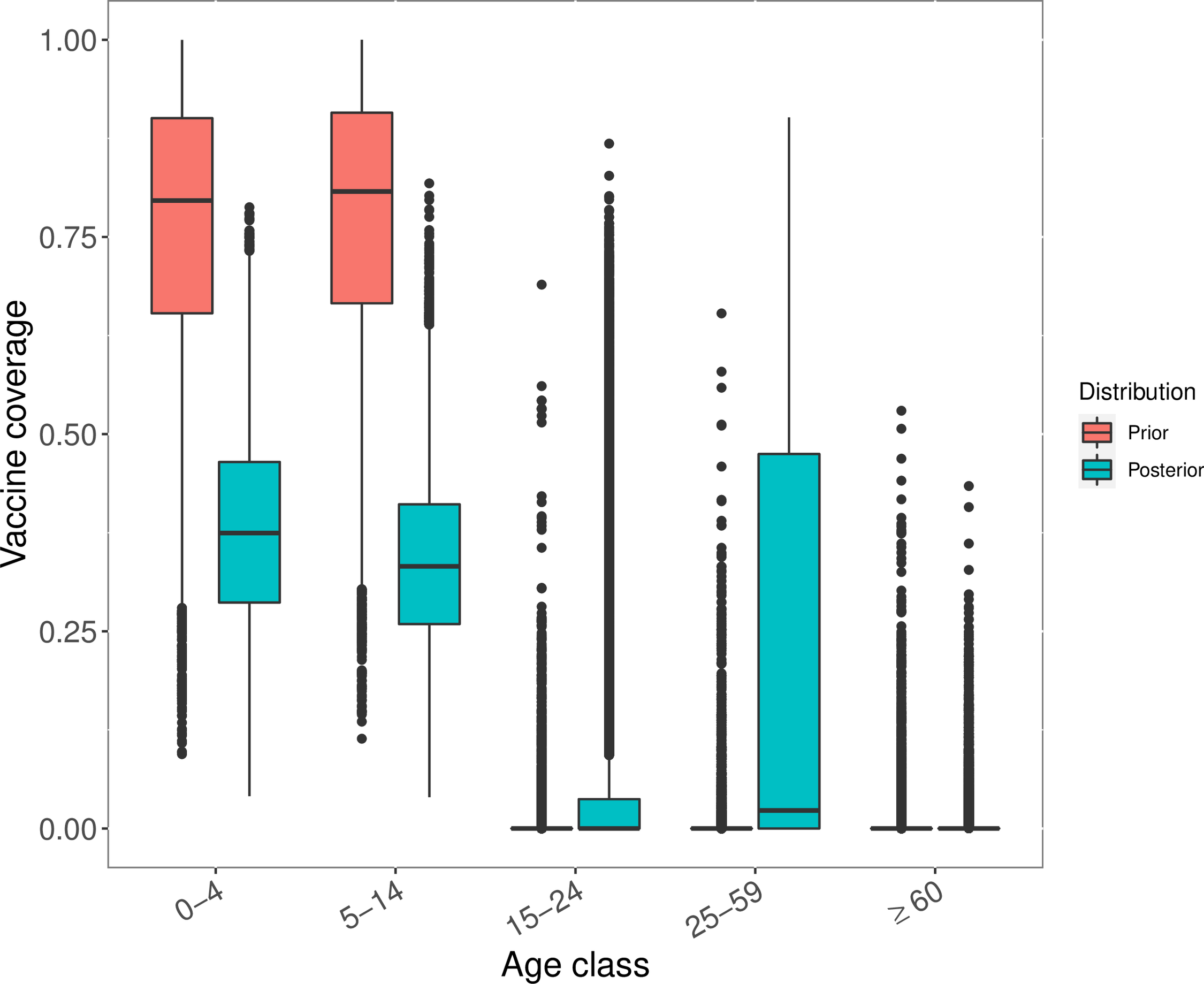

Supplement: S38 Fig — Vaccination coverage data used to generate prior distributions was taken from reference(s) listed in S1 Table. (TIF) [file pntd.0009385.s042.tif]

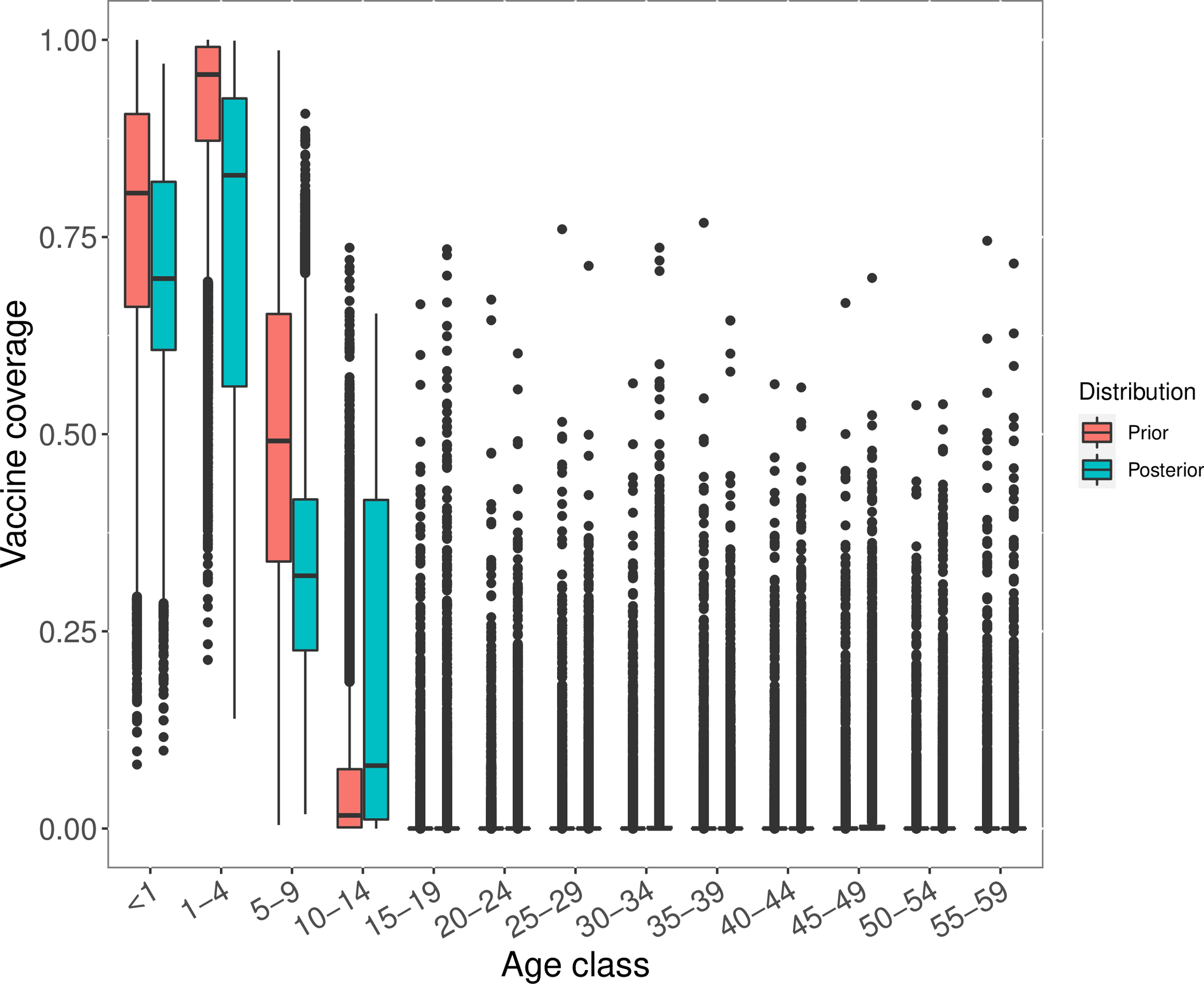

Supplement: S39 Fig — Vaccination coverage data used to generate prior distributions was taken from reference(s) listed in S1 Table. (TIF) [file pntd.0009385.s043.tif]

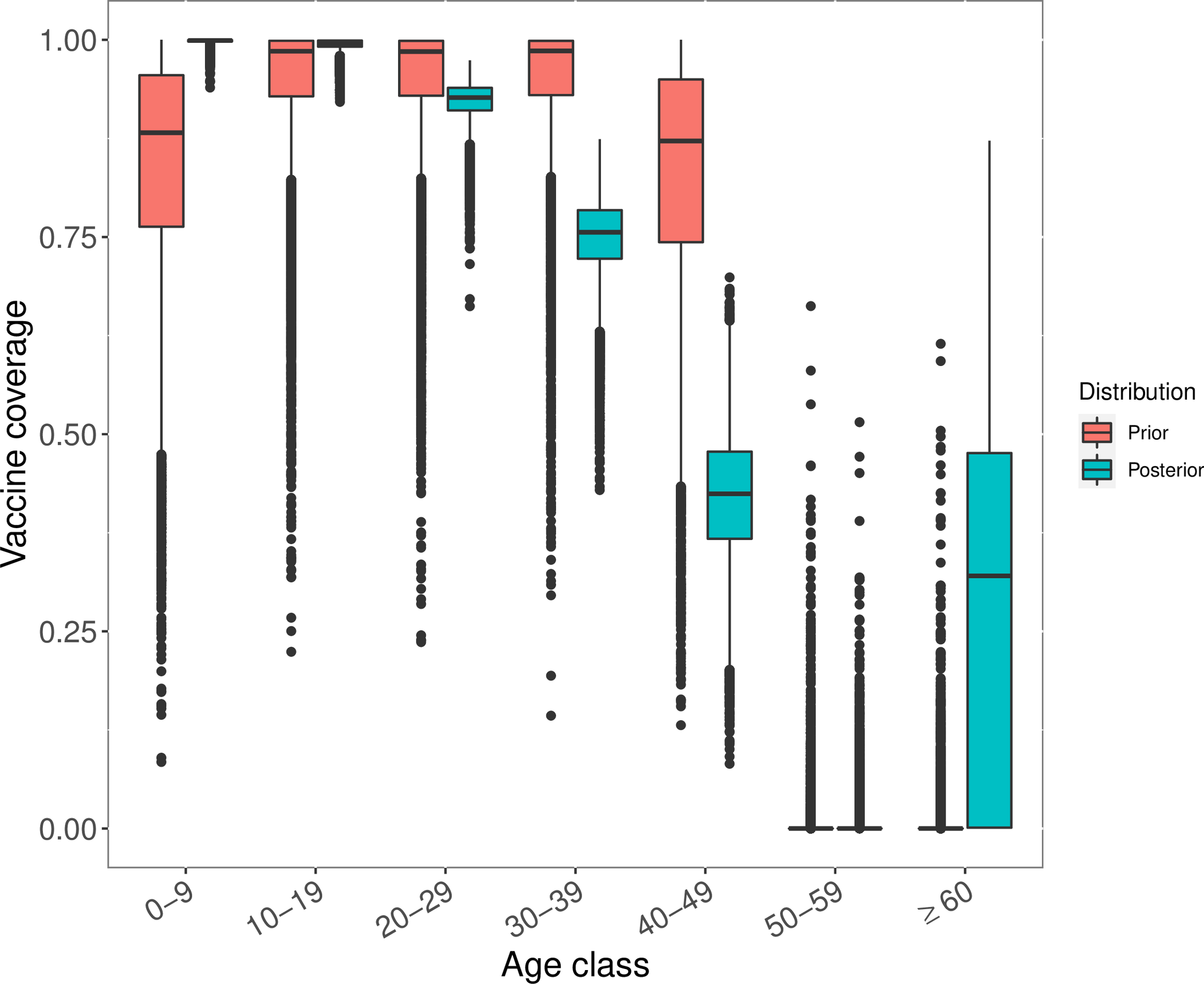

Supplement: S40 Fig — Vaccination coverage data used to generate prior distributions was taken from reference(s) listed in Table 1. (TIF) [file pntd.0009385.s044.tif]

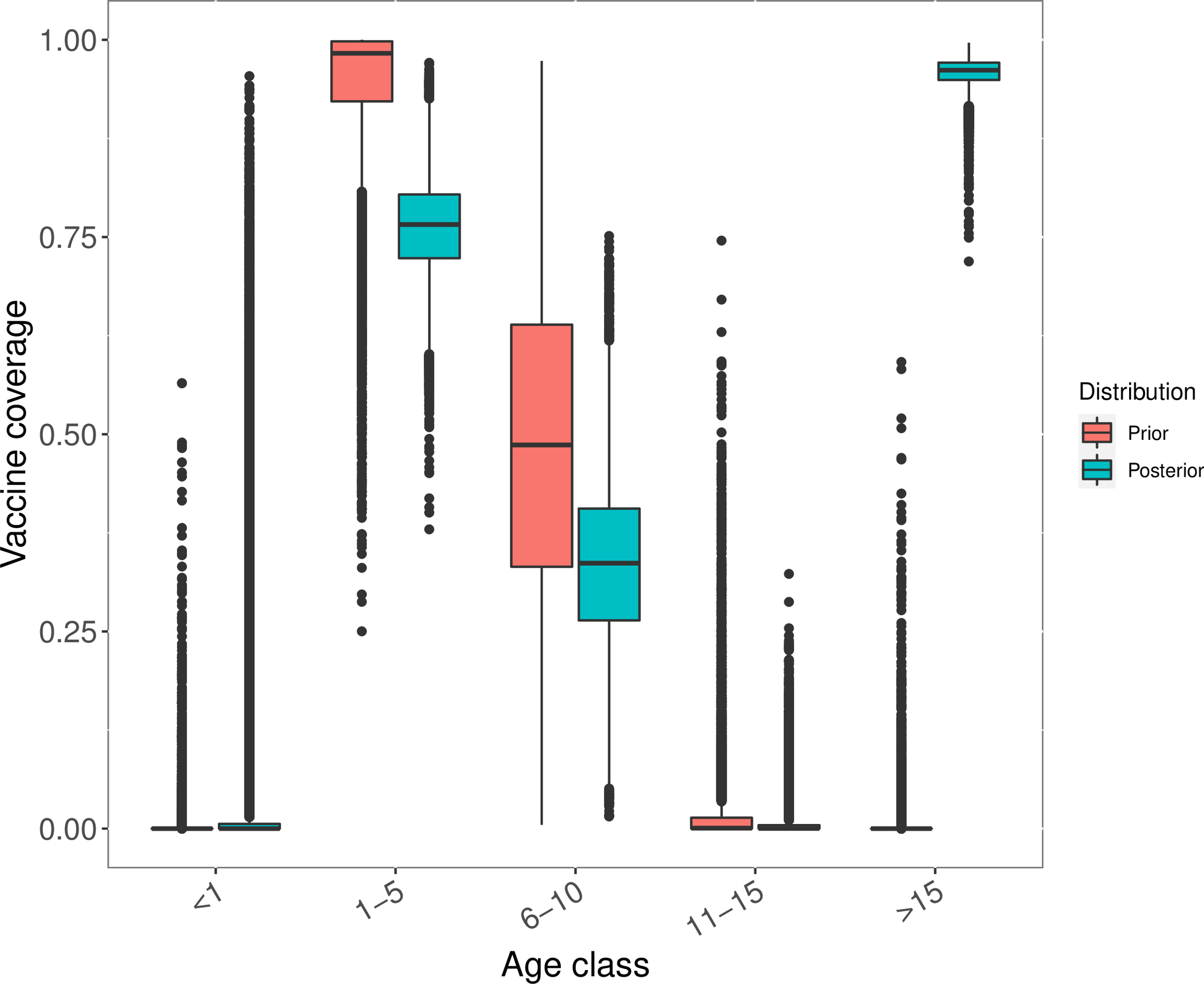

Supplement: S41 Fig — Vaccination coverage data used to generate prior distributions was taken from reference(s) listed in Table 1. (TIF) [file pntd.0009385.s045.tif]

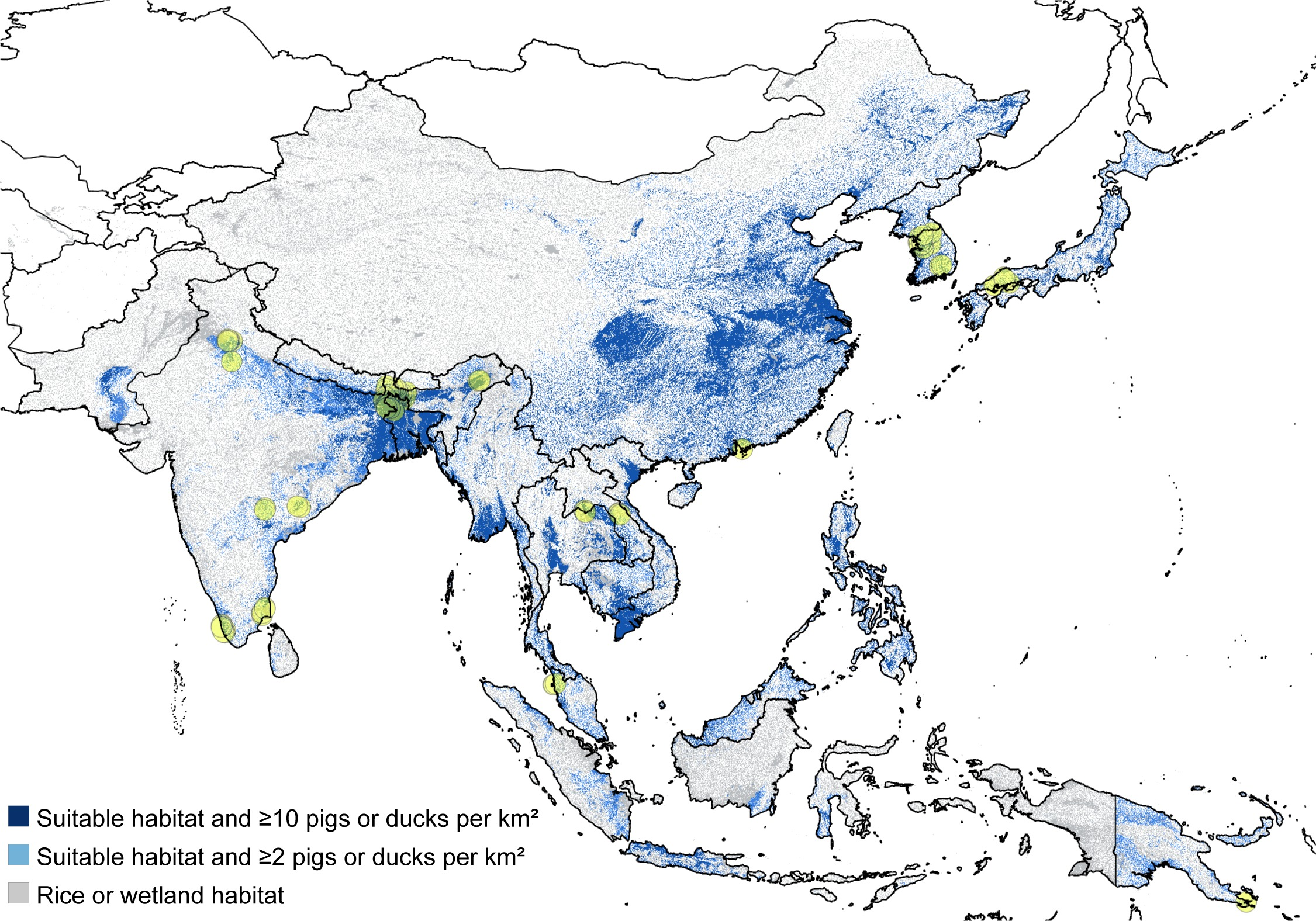

Supplement: S42 Fig — Description of at-risk geographic areas is same as in Fig 1 of main text. Yellow circles are the locations of reported JE occurrence. Information and references for the JE occurrence locations are provided in S4 Table. The base map layer was generated using the geoBoundaries Comprehensive Global Administrative Zones (CGAZ) dataset available at https://github.com/wmgeolab/geoBoundaries/raw/main/releaseData/CGAZ/geoBoundariesCGAZ_ADM0.zip. (TIF) [file pntd.0009385.s046.tif]

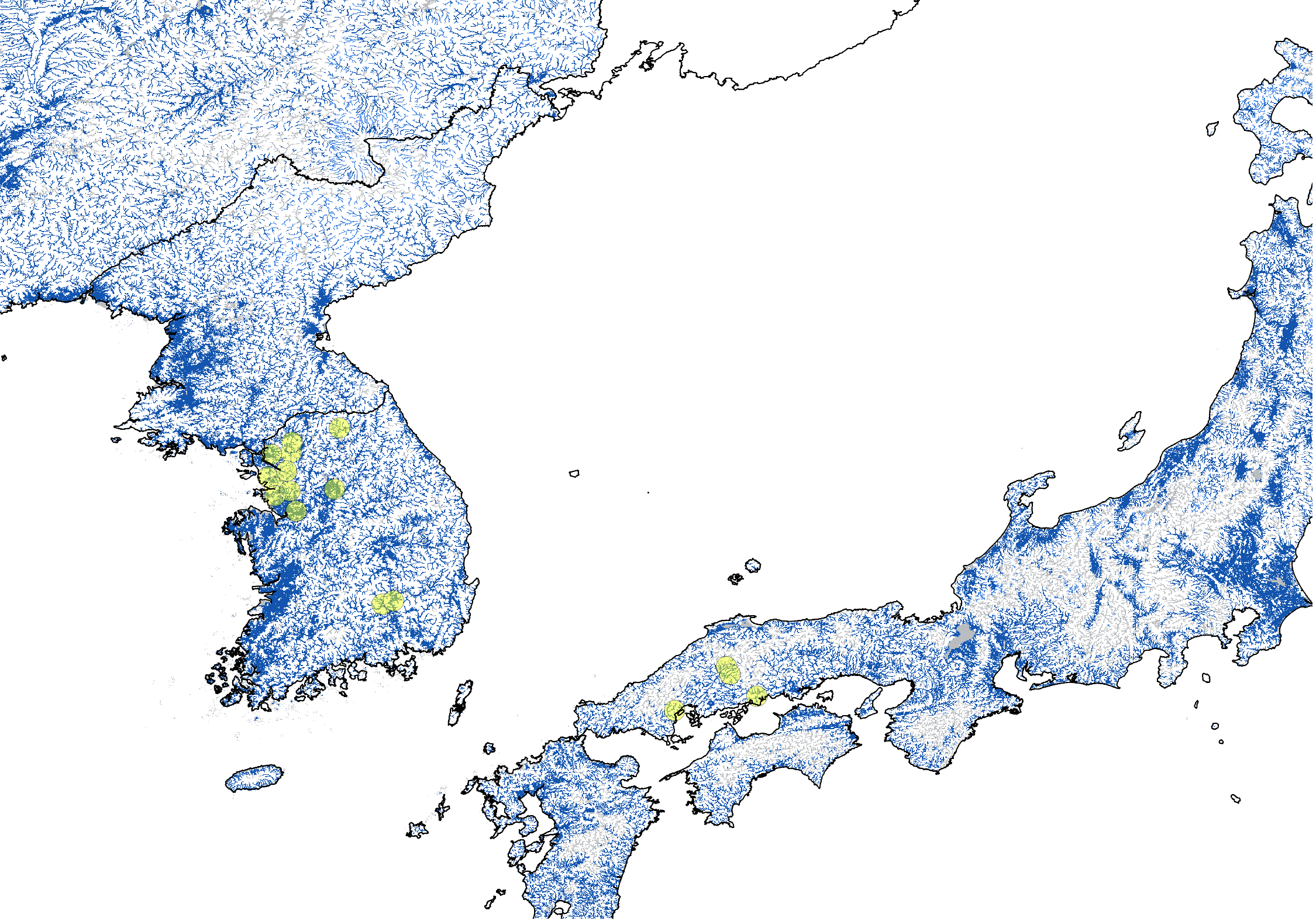

Supplement: S43 Fig — Description of at-risk geographic areas is same as in Fig 1 of main text. Yellow circles are the locations of reported JE occurrence. Information and references for the JE occurrence locations are provided in S4 Table. The base map layer was generated using the geoBoundaries Comprehensive Global Administrative Zones (CGAZ) dataset available at https://github.com/wmgeolab/geoBoundaries/raw/main/releaseData/CGAZ/geoBoundariesCGAZ_ADM0.zip. (TIF) [file pntd.0009385.s047.tif]

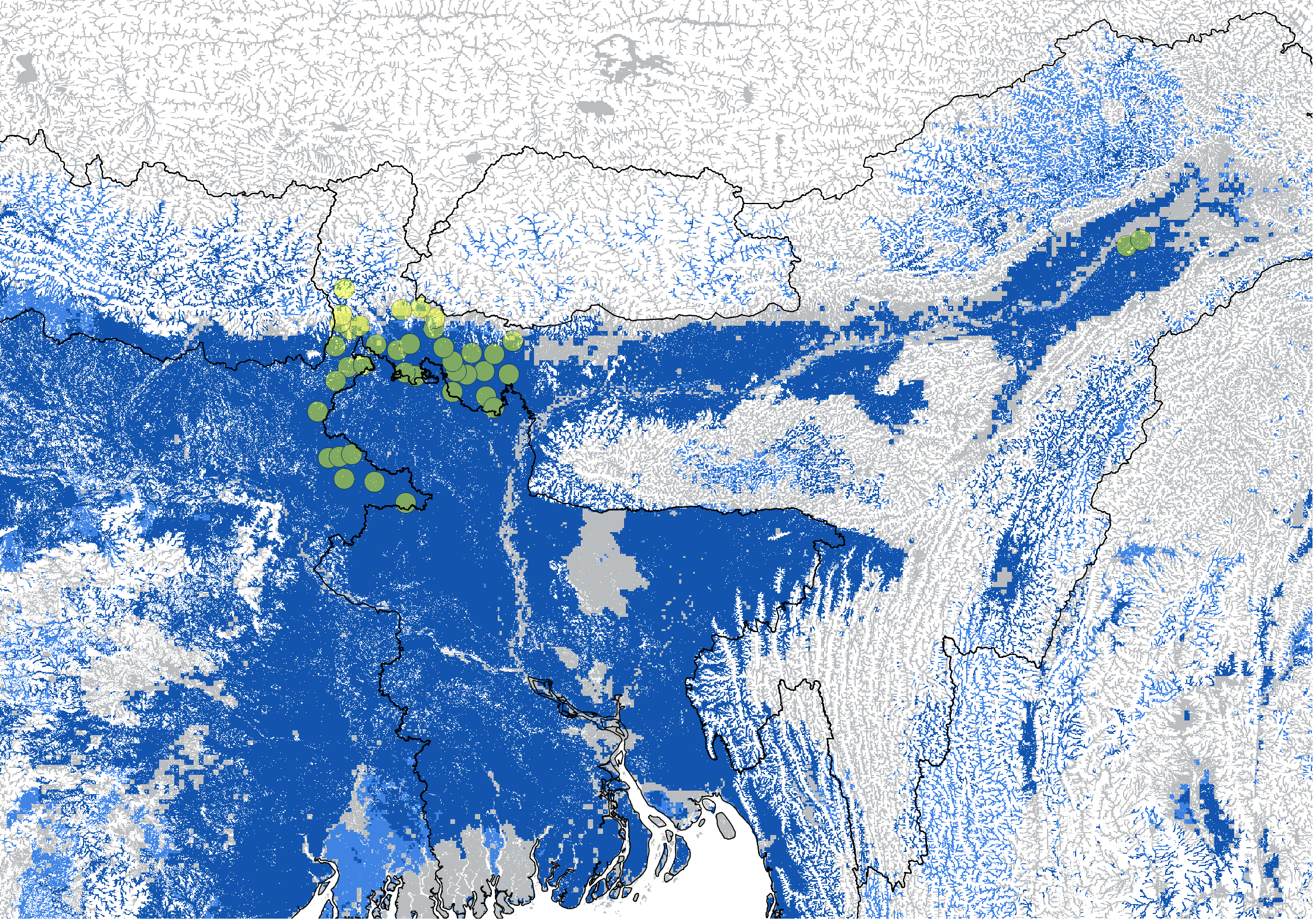

Supplement: S44 Fig — Description of at-risk geographic areas is same as in Fig 1 of main text. Yellow circles are the locations of reported JE occurrence. Information and references for the JE occurrence locations are provided in S4 Table. The base map layer was generated using the geoBoundaries Comprehensive Global Administrative Zones (CGAZ) dataset available at https://github.com/wmgeolab/geoBoundaries/raw/main/releaseData/CGAZ/geoBoundariesCGAZ_ADM0.zip. (TIF) [file pntd.0009385.s048.tif]

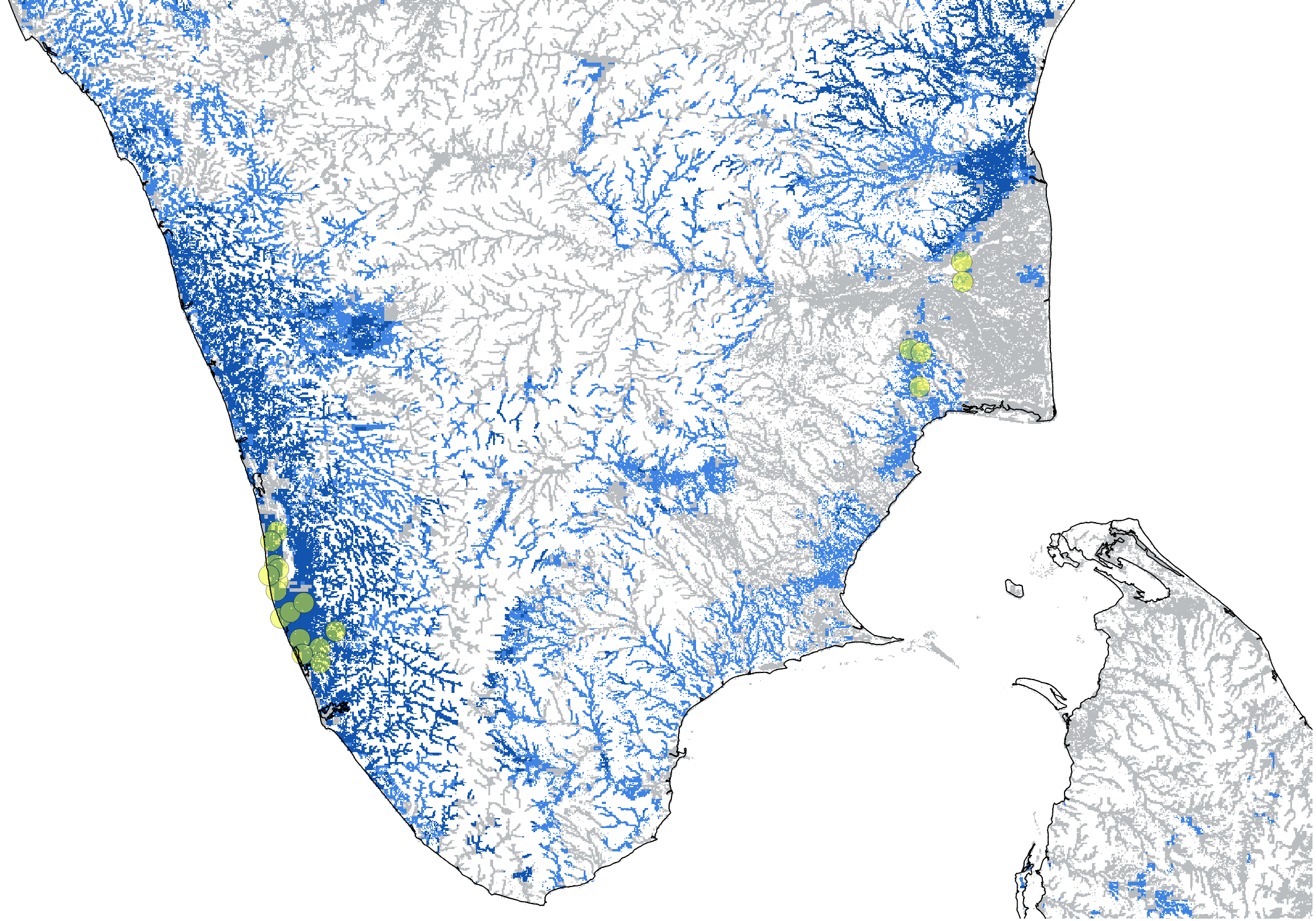

Supplement: S45 Fig — Description of at-risk geographic areas is same as in Fig 1 of main text. Yellow circles are the locations of reported JE occurrence. Information and references for the JE occurrence locations are provided in S4 Table. The base map layer was generated using the geoBoundaries Comprehensive Global Administrative Zones (CGAZ) dataset available at https://github.com/wmgeolab/geoBoundaries/raw/main/releaseData/CGAZ/geoBoundariesCGAZ_ADM0.zip. (TIF) [file pntd.0009385.s049.tif]

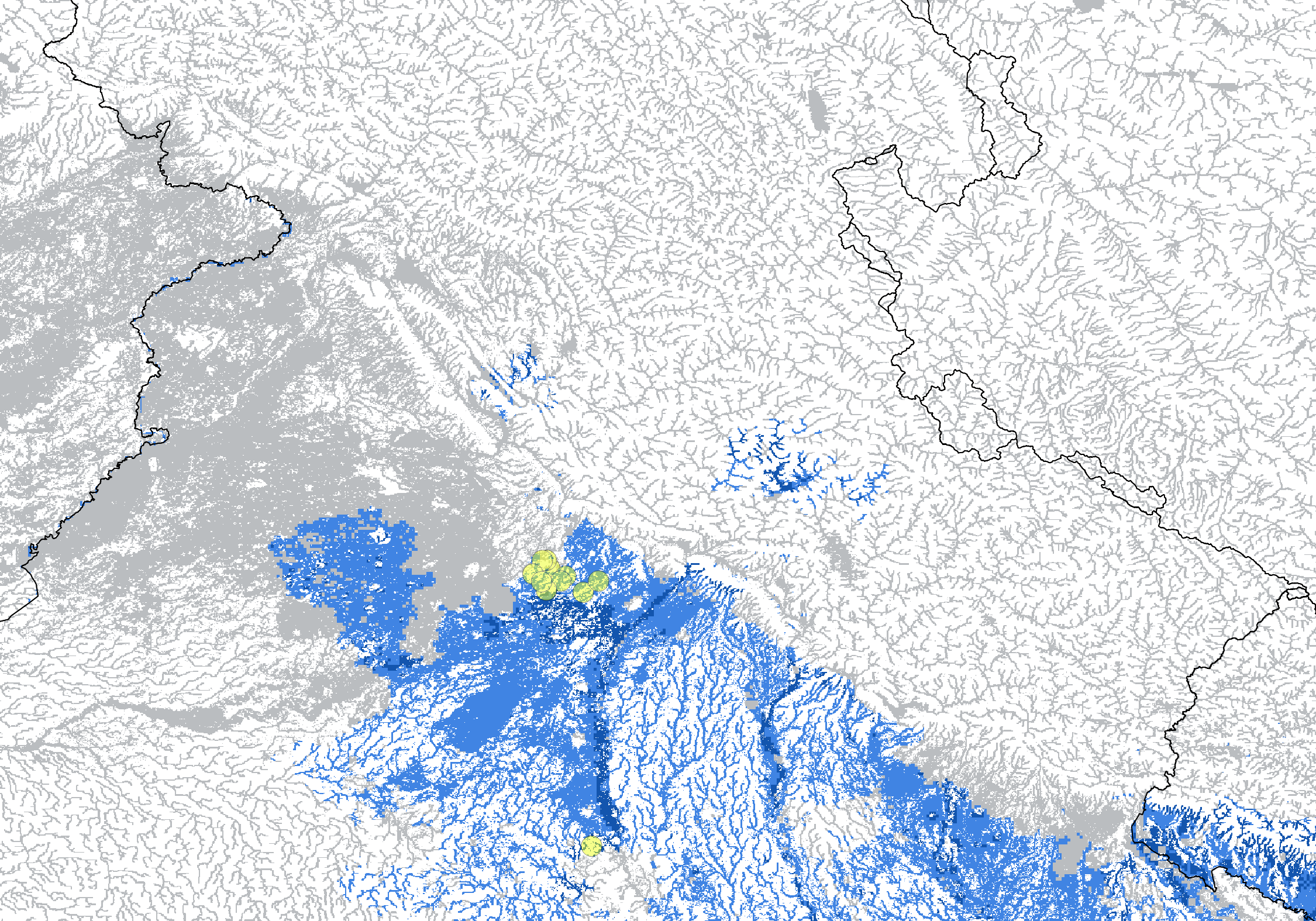

Supplement: S46 Fig — Description of at-risk geographic areas is same as in Fig 1 of main text. Yellow circles are the locations of reported JE occurrence. Information and references for the JE occurrence locations are provided in S4 Table. The base map layer was generated using the geoBoundaries Comprehensive Global Administrative Zones (CGAZ) dataset available at https://github.com/wmgeolab/geoBoundaries/raw/main/releaseData/CGAZ/geoBoundariesCGAZ_ADM0.zip. (TIF) [file pntd.0009385.s050.tif]

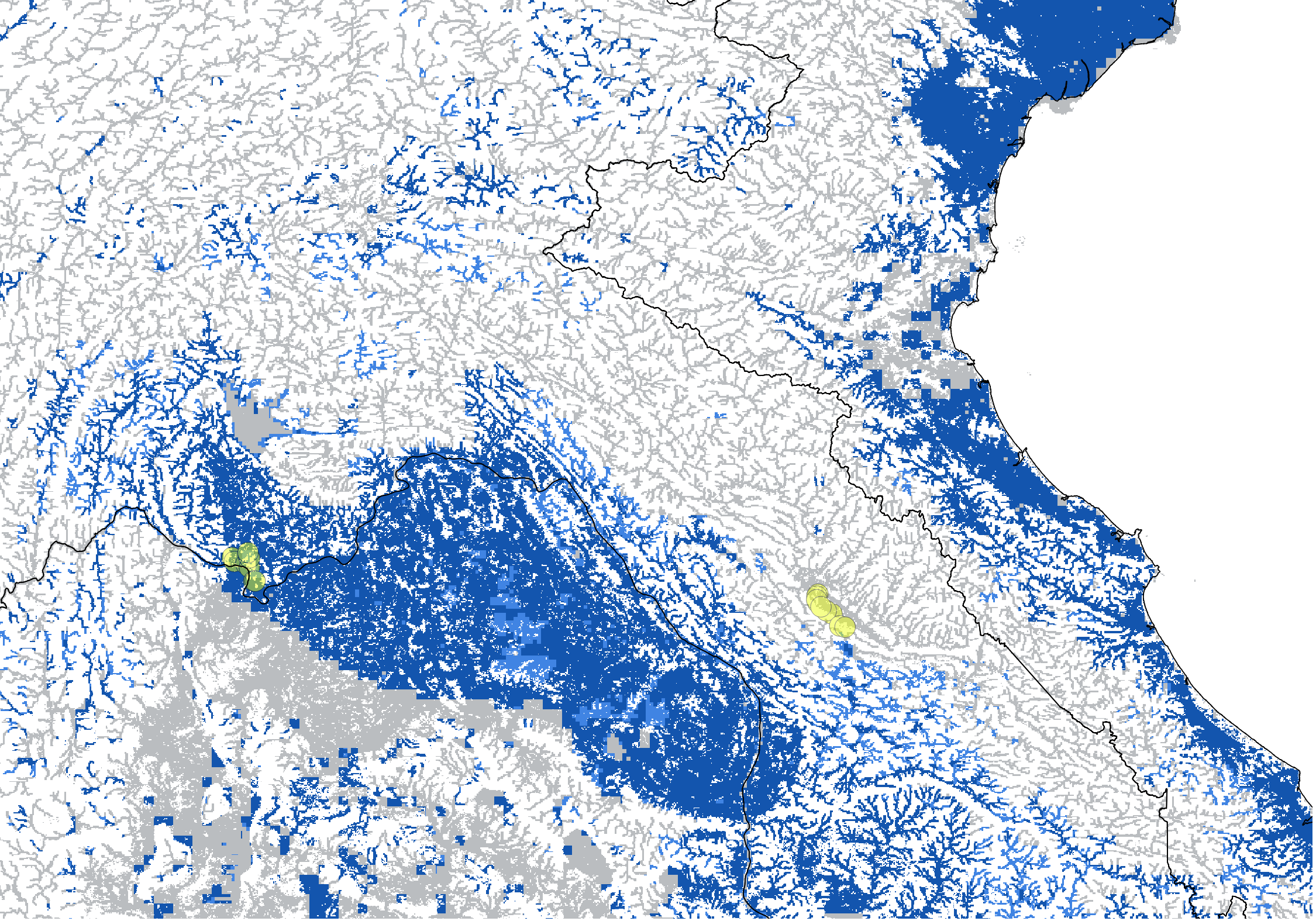

Supplement: S47 Fig — Description of at-risk geographic areas is same as in Fig 1 of main text. Yellow circles are the locations of reported JE occurrence. Information and references for the JE occurrence locations are provided in S4 Table. The base map layer was generated using the geoBoundaries Comprehensive Global Administrative Zones (CGAZ) dataset available at https://github.com/wmgeolab/geoBoundaries/raw/main/releaseData/CGAZ/geoBoundariesCGAZ_ADM0.zip. (TIF) [file pntd.0009385.s051.tif]
